# Supplementary material for: GTPBP8 is required for mitoribosomal biogenesis and mitochondrial translation
Source: Cell Mol Life Sci. 2023 Nov 16;80(12):361. doi: 10.1007/s00018-023-05014-0 (PMC10654211; doi:10.1007/s00018-023-05014-0)
Supplement: Supplementary file 1 — Supplementary file1 (PDF 508 KB) [file 18_2023_5014_MOESM1_ESM.pdf]

GTPBP8 is required for mitoribosomal biogenesis and mitochondrial translation

Supplementary Information

Supplementary figure legends

Figure S1. GTPBP8 is an evolutionarily conserved GTPase. (**A**) Phylogenetic analysis of GTPBP8. Phylogenetic tree was built using Geneious 6.1.8. (**B**) Multiple sequence alignment of GTPBP8 orthologue in prokaryotes (P38424 for *B. subtilis* Ysxc and P0A6P7 for *E.coli* Yiha) and eukaryotes (Q8N3Z3 for human GTPBP8) using clustal omega server. The GTPase domains (G1-G5) were indicated in red boxes. The N-terminal mitochondrial targeting sequence (1-46aa) was indicated by a magenta bar. (**C**) The intrinsic GTPase activity of GTPBP8. Data is an average of three independent measurements with error bars (± SD) indicated.

Figure S2. GTPBP8 is a mitochondrial protein. (**A**) Immunoblotting detection of the expressed GTPBP8-myc in U2OS cells using an anti-myc antibody. (**B**) Immunoblotting detection of the expressed GTPBP8-GFP in U2OS cells using an anti-GFP antibody. Cells expressed only the GFP-vector were used as a negative control. (**C**) Immunoblotting analysis of subcellular localization of GTPBP8-myc. After transiently expressing GTPBP8-myc in U2OS cells for 48 h, cells were fractionated and the expressed GTPBP8-myc was detected in the whole-cell lysate (WCL), cytoplasm (CYT) and isolated mitochondria (MIT) with anti-myc and anti-GTPBP8 antibodies. Antibodies against mitochondrial proteins TOM40 and Cytochrome C, and GAPDH were used as controls. (**D**) Immunoblotting analysis of submitochondrial localization of GTPBP8-myc using proteinase K digestion of isolated mitochondria from GTPBP8-myc expressed cells. Mitochondria were subjected to hypotonic swelling or permeabilization with 1% NP-40, treated with 100 μg/mL proteinase K as indicated.

Figure S3. Effects of GTPBP8 depletion on protein abundance of the oxidative phosphorylation complexes and functional networks by mass spectroscopy analysis. (**A**) Main functionally affected biological processes by GTPBP8 depletion. (**B**) Steady-state protein levels of oxidative phosphorylation complexes after depletion of GTPBP8.

Figure S4. Loss of GTPBP8 caused reduction of mitoribosomal protein abundance. (**A**) Effects of GTPBP8 depletion on the steady-state levels of mitoribosomal proteins and mitoribosomal assembly related factors by label-free quantitative mass spectrometry (GTPBP8 silencing versus WT). Proteins with significant changes are labeled in yellow, and those without significant changes are indicated in cyan. U2OS cells were treated with control or GTPBP8 siRNA for 5 days. The silencing efficiency of GTPBP8 is shown in panel B. (**C**) Effects of GTPBP8 loss on the steady-state levels of mitoribosomal proteins.

Figure S5. Loss of GTPBP8 caused defects in mitochondrial translation. (**A**) Effects of GTPBP8 loss on the steady state levels of mitoribosomal proteins. GTPBP8 was depleted using siRNA #2. (**B**) Metabolic labeling of mitochondrial translation products with [^{35}S]-methionine/cysteine in U2OS cells treated with GTPBP8 siRNA #2 or expression of GTPBP8-myc. (**C**) Quantification of ^{35}S intensity in panel B for all the mtDNA-encoded translation products relative to the corresponding intensity in coomassie staining.

Figure S6. The steady state levels of GTPBP8. Immunoblotting for the steady state levels of GTPBP8 in the wild type, GTPBP8-depleted, and rescue cell samples.

Supplemental Table S1. High confidence interactors (HCIs) of GTPBP8 screened by BioID proximity protein purification and mass spectroscopy analysis. Interactions are assigned as HCIs

based on peptide-spectrum match (PSM) of control samples and further refined by using CRAPome contaminant repository data (frequency cut-off ≥ 20% and average PSM change ≤ 3).

Supplemental Table S2. Label-free quantification (LFQ) intensities derived from MaxQuant for U2OS cells treated with control siRNA or GTPBP8 siRNA. N/A is short for non-applicable.

Figure S1

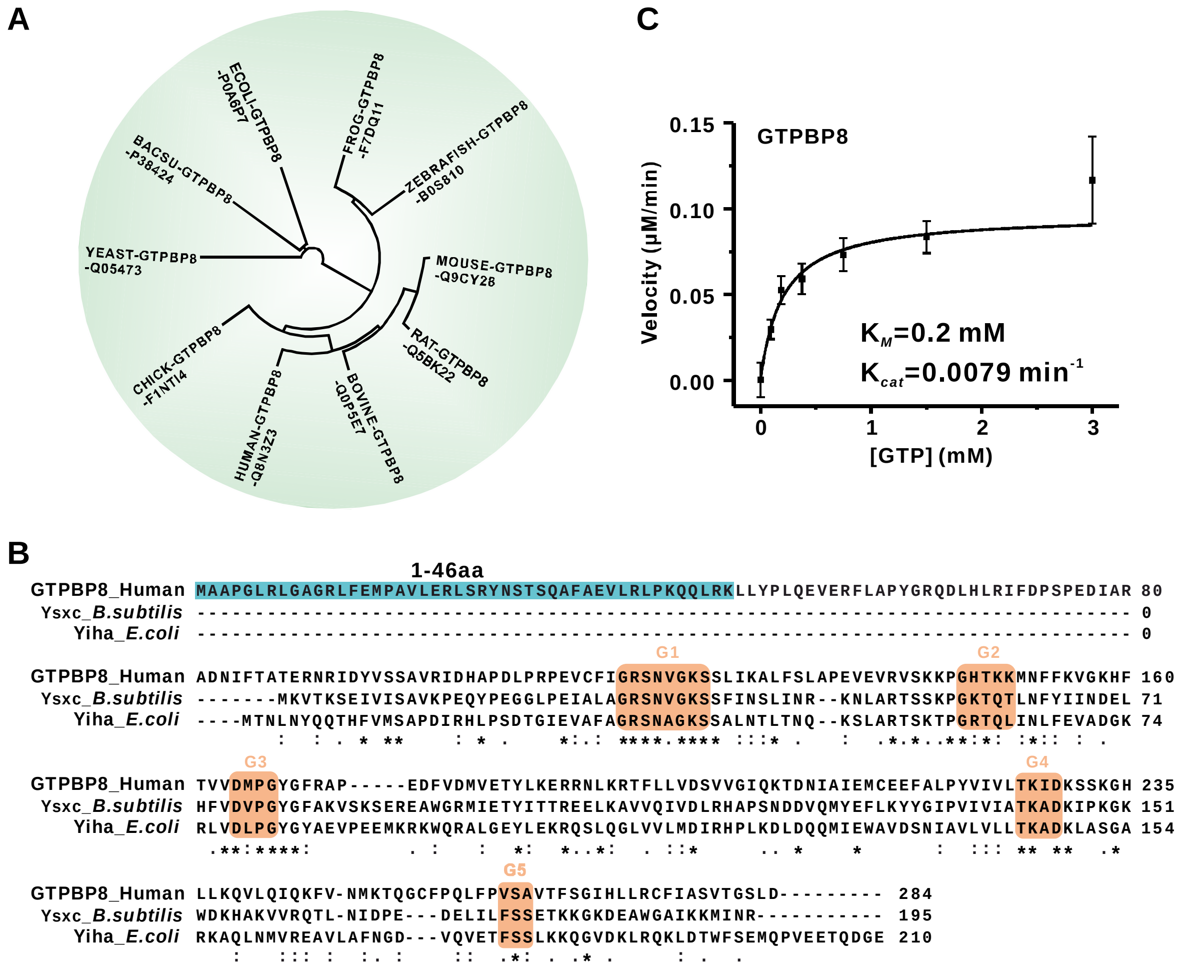
A
ECOLI-GTPBP8
-P0A6P7
FROG-GTPBP8
-F7DQ11
ZEBRAFISH-GTPBP8
-B0S810
BACSU-GTPBP8
-P38424
YEAST-GTPBP8
-Q05473
MOUSE-GTPBP8
-Q9CY28
RAT-GTPBP8
-Q5BK22
CHICK-GTPBP8
-F1NTI4
HUMAN-GTPBP8
-Q8N3Z3
BOVINE-GTPBP8
-Q0P5E7
C
GTPBP8
Velocity (μM/min)
0.15
0.10
0.05
0.00
0
1
2
3
[GTP] (mM)
K_M=0.2 mM
K_{cat}=0.0079 min^{-1}
B
1-46aa
GTPBP8_Human MAAPGLRLGAGRLFEMPAVLERLSRYNSTSQAFAEVLRLPKQQLRKLLYPLQEVERFLAPYGRQDLHLRIFDPSPEDIAR 80
Ysxc_B.subtilis -- 0
Yiha_E.coli -- 0
G1
G2
GTPBP8_Human ADNIFTATERNRIDYVSSAVRIDHAPDLPRPEVCFIGRSNVGKSSLIKALFSLAPEVEVRVSKKPGHTKKMNFFKVGKHF 160
Ysxc_B.subtilis -------MKVTKSEIVISAVKPEQYPEGGLPEIALAGRSNVGKSSFINSLINR--KNLARTSSKPGKTQTLNFYIINDEL 71
Yiha_E.coli ----MTNLNYQQTHFVMSAPDIRHLPSDTGIEVAFAGRSNAGKSSALNTLTNQ--KSLARTSKTPGRTQLINLFEVADGK 74
G3
G4
GTPBP8_Human TVVDMPGYGFRAP-----EDFVDMVETYLKERRNLKRTFLLVDSVVGIQKTDNIAIEMCEEFALPYVIVLTKIDKSSKGH 235
Ysxc_B.subtilis HFVDVPGYGFAKVSKSEREAWGRMIETYITTREELKAVVQIVDLRHAPSNDDVQMYEFLKYYGIPVIVIATKADKIPKGK 151
Yiha_E.coli RLVDLPGYGYAEVPEEMKRKWQRALGEYLEKRQSLQGLVVLMDIRHPLKDLDQQMIEWAVDSNIAVLVLLTKADKLASGA 154
G5
GTPBP8_Human LLKQVLQIQKFV-NMKTQGCFPQLFPVSAVTFSGIHLLRCFIASVTGSLD--------- 284
Ysxc_B.subtilis WDKHAKVVRQTL-NIDPE---DELILFSSETKKGKDEAWGAIKKMINR----------- 195
Yiha_E.coli RKAQLNMVREAVLAFNGD---VQVETFSSLKKQGVDKLRQKLDTWFSEMQPVEETQDGE 210

Figure S2

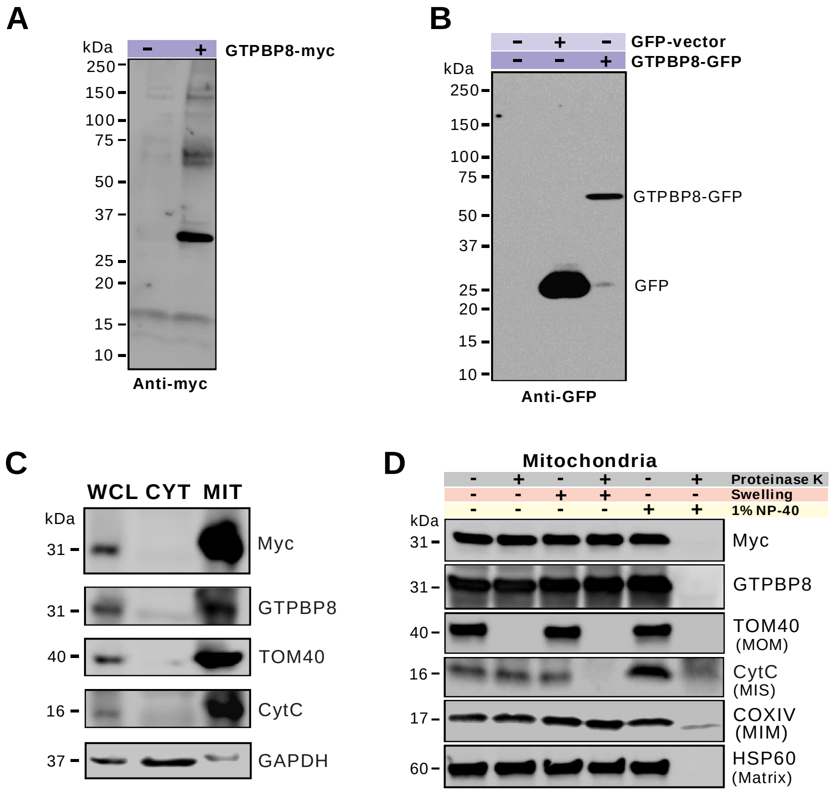

A
kDa
GTPBP8-myc
250
150
100
75
50
37
25
20
15
10
Anti-myc
B
GFP-vector
GTPBP8-GFP
GTPBP8-GFP
GFP
Anti-GFP
C
WCL CYT MIT
Myc
GTPBP8
TOM40
CytC
GAPDH
D
Mitochondria
Proteinase K
Swelling
1% NP-40
Myc
GTPBP8
TOM40
(MOM)
CytC
(MIS)
COXIV
(MIM)
HSP60
(Matrix)

Figure S3

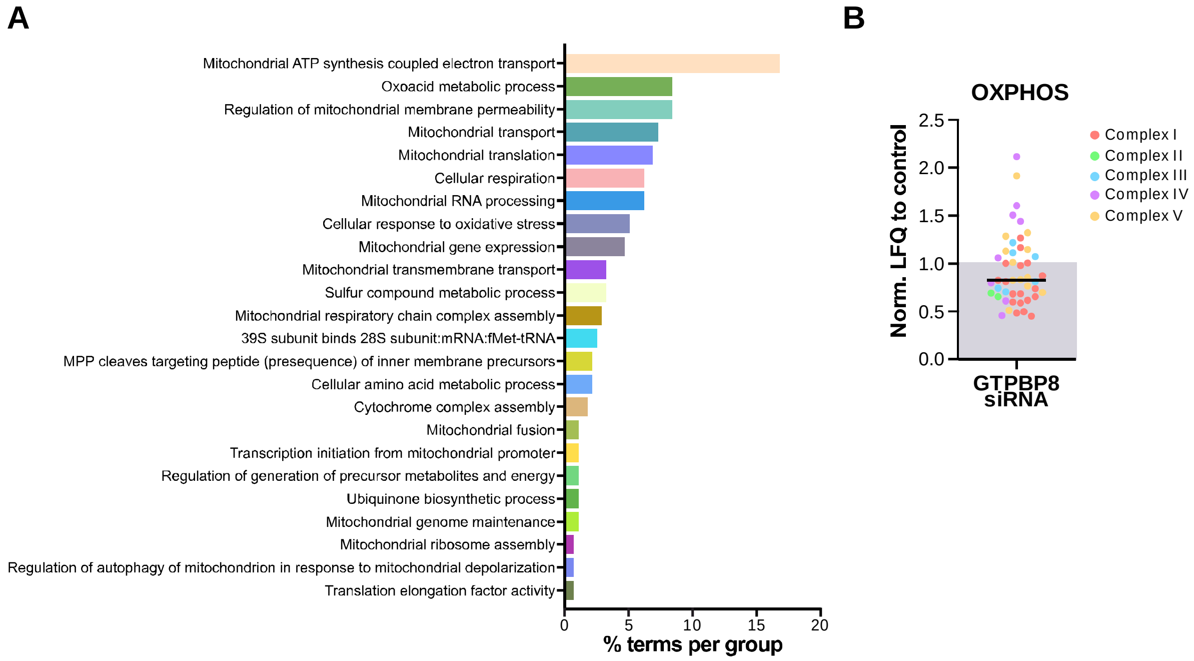
A
Mitochondrial ATP synthesis coupled electron transport
Oxoacid metabolic process
Regulation of mitochondrial membrane permeability
Mitochondrial transport
Mitochondrial translation
Cellular respiration
Mitochondrial RNA processing
Cellular response to oxidative stress
Mitochondrial gene expression
Mitochondrial transmembrane transport
Sulfur compound metabolic process
Mitochondrial respiratory chain complex assembly
39S subunit binds 28S subunit:mRNA:fMet-tRNA
MPP cleaves targeting peptide (presequence) of inner membrane precursors
Cellular amino acid metabolic process
Cytochrome complex assembly
Mitochondrial fusion
Transcription initiation from mitochondrial promoter
Regulation of generation of precursor metabolites and energy
Ubiquinone biosynthetic process
Mitochondrial genome maintenance
Mitochondrial ribosome assembly
Regulation of autophagy of mitochondrion in response to mitochondrial depolarization
Translation elongation factor activity
0
5
10
15
20
% terms per group
B
OXPHOS
Norm. LFQ to control
2.5
2.0
1.5
1.0
0.5
0.0
GTPBP8
siRNA
Complex I
Complex II
Complex III
Complex IV
Complex V

Figure S4

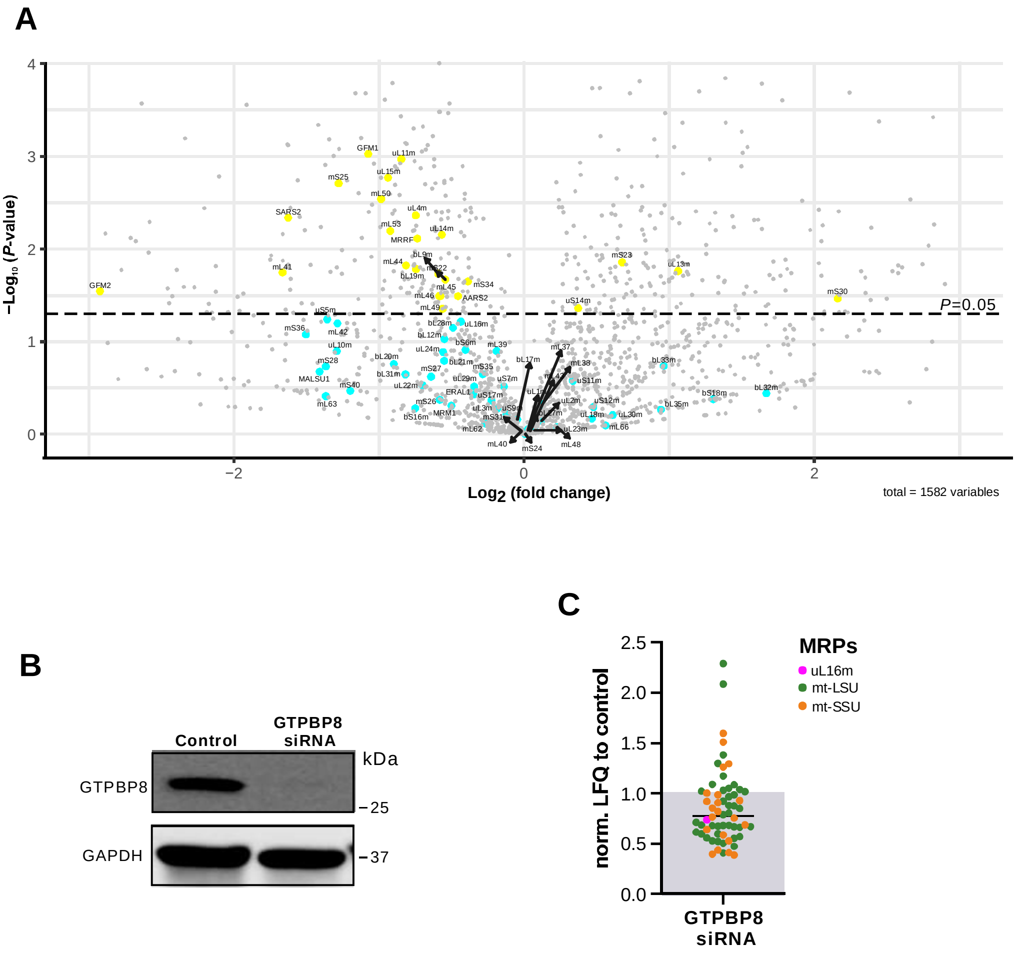
A
−Log10 (P-value)
Log2 (fold change)
P=0.05
total = 1582 variables
B
Control
GTPBP8 siRNA
kDa
GTPBP8
GAPDH
25
37
C
norm. LFQ to control
MRPs
uL16m
mt-LSU
mt-SSU
GTPBP8 siRNA

Figure S5

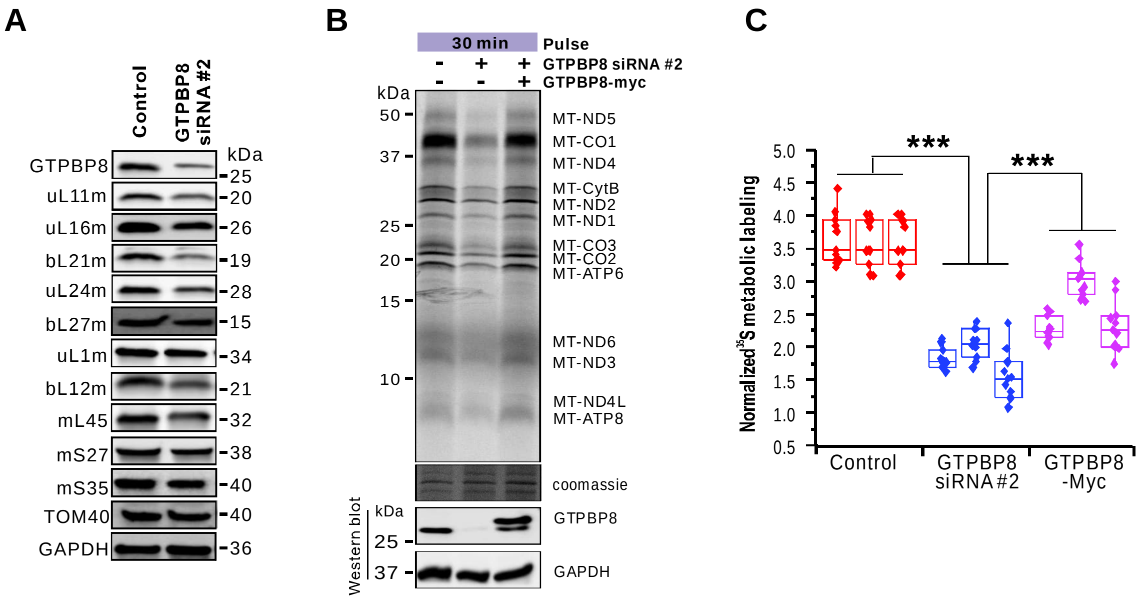

A
Control
GTPBP8 siRNA #2
kDa
GTPBP8
uL11m
uL16m
bL21m
uL24m
bL27m
uL1m
bL12m
mL45
mS27
mS35
TOM40
GAPDH
25
20
26
19
28
15
34
21
32
38
40
40
36
B
30 min
Pulse
GTPBP8 siRNA #2
GTPBP8-myc
kDa
50
37
25
20
15
10
MT-ND5
MT-CO1
MT-ND4
MT-CytB
MT-ND2
MT-ND1
MT-CO3
MT-CO2
MT-ATP6
MT-ND6
MT-ND3
MT-ND4L
MT-ATP8
coomassie
Western blot
GTPBP8
GAPDH
C
***
***
Normalized 35S metabolic labeling
5.0
4.5
4.0
3.5
3.0
2.5
2.0
1.5
1.0
0.5
Control
GTPBP8 siRNA#2
GTPBP8 -Myc

Figure S6

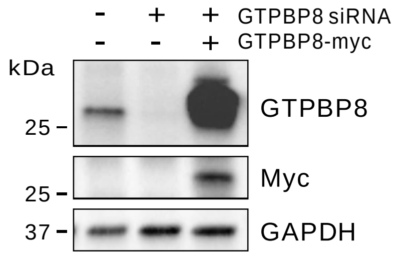

Table S1: The high confidence interactions obtained from GTPBP8 BioID purification. The raw data was analyzed by Proteome Discoverer and the peptide-spectrum matching (PSM) vaules were used for identification of high confidence interactions (HCIs)

Bait_ID	Prey_ID	Gene names (primary)	New MRP Name	Functional Group	Entry name	GTPBP8_Biological replicate 1	GTPBP8_Biological replicate 2	GTPBP8_Biological replicate 3	GTPBP8_Biological replicate 4	GTPBP8_psm_sum	GTPBP8_Frequency	Control_Biological replicate 1	Control_Biological replicate 2	Control_Biological replicate 3	Control_Biological replicate 4	Control_psm_sum	Control_Frequency
Q8N3Z3	O95573	ACSL3		Fatty acid metabolism	ACSL3_HUMAN	**7**	**2**	**3**	**3**	**15**	**4**	0	0	0	0	0	0
Q8N3Z3	P30084	ECHS1		Fatty acid metabolism	ECHM_HUMAN	**3**	**3**	**4**	**2**	**12**	**4**	0	0	0	0	0	0
Q8N3Z3	P40939	HADHA		Fatty acid metabolism	ECHA_HUMAN	**10**	**12**	**13**	**10**	**45**	**4**	0	0	1	1	2	2
Q8N3Z3	P49748	ACADVL		Fatty acid metabolism	ACADV_HUMAN	**8**	**8**	**9**	**7**	**32**	**4**	0	1	1	0	2	2
Q8N3Z3	P49753	ACOT2		Fatty acid metabolism	ACOT2_HUMAN	**57**	**53**	**62**	**55**	**227**	**4**	12	0	0	0	12	1
Q8N3Z3	P55084	HADHB		Fatty acid metabolism	ECHB_HUMAN	**6**	**7**	**5**	**7**	**25**	**4**	0	0	0	0	0	0
Q8N3Z3	Q7Z2W9	MRPL21	bL21m	Mitochondrial translation	RM21_HUMAN	**2**	**2**	**3**	**3**	**10**	**4**	0	0	0	0	0	0
Q8N3Z3	Q7Z7F7	MRPL55	bL31m	Mitochondrial translation	RM55_HUMAN	**2**	**3**	**2**	**2**	**9**	**4**	0	0	0	0	0	0
Q8N3Z3	Q8N983	MRPL43	mL43	Mitochondrial translation	RM43_HUMAN	**3**	**3**	**2**	**3**	**11**	**4**	0	0	0	0	0	0
Q8N3Z3	Q9BRJ2	MRPL45	mL45	Mitochondrial translation	RM45_HUMAN	**2**	**3**	**2**	**2**	**9**	**4**	0	0	0	0	0	0
Q8N3Z3	Q9BYD6	MRPL1	uL1m	Mitochondrial translation	RM01_HUMAN	**2**	**2**	**3**	**3**	**10**	**4**	0	0	0	0	0	0
Q8N3Z3	Q9H2W6	MRPL46	mL46	Mitochondrial translation	RM46_HUMAN	**5**	**5**	**5**	**4**	**19**	**4**	0	0	0	0	0	0
Q8N3Z3	Q9H9J2	MRPL44	mL44	Mitochondrial translation	RM44_HUMAN	**2**	**4**	**2**	**4**	**12**	**4**	0	0	0	0	0	0
Q8N3Z3	Q9NQ50	MRPL40	mL40	Mitochondrial translation	RM40_HUMAN	**4**	**2**	**4**	**2**	**12**	**4**	1	0	1	0	2	2
Q8N3Z3	Q9NSE4	IARS2		Mitochondrial translation	SYIM_HUMAN	**6**	**5**	**6**	**4**	**21**	**4**	0	0	0	0	0	0
Q8N3Z3	Q9Y2Z4	YARS2		Mitochondrial translation	SYYM_HUMAN	**1**	**3**	**2**	**2**	**8**	**4**	0	0	0	0	0	0
Q8N3Z3	O75489	NDUFS3		Respiratory electron transport and the citric acid (TCA) cycle	NDUS3_HUMAN	**5**	**5**	**3**	**3**	**16**	**4**	0	0	0	0	0	0
Q8N3Z3	P07954	FH		Respiratory electron transport and the citric acid (TCA) cycle	FUMH_HUMAN	**6**	**2**	**5**	**2**	**15**	**4**	0	0	0	0	0	0
Q8N3Z3	P10515	DLAT		Respiratory electron transport and the citric acid (TCA) cycle	ODP2_HUMAN	**15**	**17**	**16**	**16**	**64**	**4**	2	2	4	3	11	4
Q8N3Z3	P10606	COX5B		Respiratory electron transport and the citric acid (TCA) cycle	COX5B_HUMAN	**2**	**2**	**2**	**2**	**8**	**4**	0	0	0	0	0	0
Q8N3Z3	P13073	COX4I1		Respiratory electron transport and the citric acid (TCA) cycle	COX41_HUMAN	**2**	**3**	**2**	**3**	**10**	**4**	0	0	0	0	0	0
Q8N3Z3	P48047	ATP5PO		Respiratory electron transport and the citric acid (TCA) cycle	ATPO_HUMAN	**3**	**3**	**3**	**2**	**11**	**4**	0	0	0	0	0	0
Q8N3Z3	P56134	ATP5MF		Respiratory electron transport and the citric acid (TCA) cycle	ATPK_HUMAN	**2**	**2**	**2**	**2**	**8**	**4**	0	0	0	0	0	0
Q8N3Z3	Q16718	NDUFA5		Respiratory electron transport and the citric acid (TCA) cycle	NDUA5_HUMAN	**2**	**3**	**1**	**2**	**8**	**4**	0	0	0	0	0	0
Q8N3Z3	Q9BSH4	TACO1		Respiratory electron transport and the citric acid (TCA) cycle	TACO1_HUMAN	**7**	**4**	**4**	**5**	**20**	**4**	0	0	0	0	0	0
Q8N3Z3	O00429	DNM1L			DNM1L_HUMAN	**15**	**10**	**14**	**12**	**51**	**4**	2	3	3	4	12	4
Q8N3Z3	O43615	TIMM44			TIM44_HUMAN	**3**	**3**	**4**	**3**	**13**	**4**	0	0	0	0	0	0
Q8N3Z3	O76031	CLPX			CLPX_HUMAN	**4**	**2**	**2**	**3**	**11**	**4**	0	0	0	0	0	0
Q8N3Z3	O94925	GLS			GLSK_HUMAN	**10**	**11**	**11**	**10**	**42**	**4**	0	1	0	0	1	1
Q8N3Z3	O95202	LETM1			LETM1_HUMAN	**2**	**4**	**4**	**2**	**12**	**4**	0	0	0	0	0	0
Q8N3Z3	P32322	PYCR1			P5CR1_HUMAN	**20**	**17**	**19**	**18**	**74**	**4**	1	0	2	1	4	3
Q8N3Z3	Q12849	GRSF1			GRSF1_HUMAN	**15**	**14**	**12**	**15**	**56**	**4**	1	1	0	2	4	3
Q8N3Z3	Q8N3Z3	GTPBP8			GTPB8_HUMAN	**59**	**57**	**59**	**59**	**234**	**4**	0	0	0	0	0	0
Q8N3Z3	Q96C36	PYCR2			P5CR2_HUMAN	**4**	**0**	**4**	**5**	**13**	**3**	0	0	0	0	0	0
Q8N3Z3	Q9BVS5	TRMT61B			TR61B_HUMAN	**3**	**5**	**9**	**6**	**23**	**4**	0	0	0	0	0	0
Q8N3Z3	Q9BX68	HINT2			HINT2_HUMAN	**3**	**4**	**4**	**3**	**14**	**4**	0	0	0	0	0	0
Q8N3Z3	Q9HAV7	GRPEL1			GRPE1_HUMAN	**3**	**3**	**5**	**2**	**13**	**4**	0	1	1	1	3	3
Q8N3Z3	Q9Y4W6	AFG3L2			AFG32_HUMAN	**11**	**8**	**9**	**8**	**36**	**4**	0	0	0	0	0	0

Table S2: Proteomics analysis of siRNA-mediated depletion of GTPBP8. The data were processed by Maxquant using relative label-free quantification (LFQ)

Majority protein ID	Gene names	New MRP Name	LFQ intensity Control 1	LFQ intensity Control 2	LFQ intensity Control 3	LFQ intensity siRNA-GTPBP8_1	LFQ intensity siRNA-GTPBP8_2	LFQ intensity siRNA-GTPBP8_3	Average_Control	Average_siRNA-GTPBP8	p-value	Fold_change (siRNA-GTPBP8/Control)	Log2(fold change)
Q9BYD3	MRPL4	uL4m	1221400000	1244000000	1170600000	566980000	759910000	840670000	1212000000	722520000	0.004328022	0.596138614	-0.74628027
Q9BYD2	MRPL9	bL9m	301680000	301850000	231920000	196820000	186180000	171250000	278483333.3	184750000	0.018520594	0.663414926	-0.592016623
Q9Y3B7	MRPL11	uL11m	1237000000	1100700000	1170400000	573140000	735480000	642800000	1169366667	650473333.3	0.001068258	0.556261224	-0.846165553
Q9BYD1	MRPL13	uL13m	203760000	254360000	331790000	454250000	525980000	668370000	263303333.3	549533333.3	0.017320783	2.087073211	1.061481209
Q6P1L8	MRPL14	uL14m	569000000	577150000	597550000	435170000	421140000	320560000	581233333.3	392290000	0.00700306	0.67492688	-0.567196883
Q9P015	MRPL15	uL15m	2056900000	1809800000	1907100000	833930000	1000400000	1179100000	1924600000	1004476667	0.00170071	0.52191451	-0.938114582
P49406	MRPL19	bL19m	690070000	945940000	771960000	419680000	495270000	521960000	802656666.7	478970000	0.01646398	0.596730856	-0.744847716
Q8IXM3	MRPL41	mL41	389490000	376700000	298830000	164320000	171610000	0	355006666.7	111976666.7	0.017978949	0.315421307	-1.664647978
Q9H9J2	MRPL44	mL44	914900000	761090000	724160000	333450000	507420000	524270000	800050000	455046666.7	0.015031472	0.568772785	-0.814075659
Q9BRJ2	MRPL45	mL45	340260000	384600000	389830000	193930000	236380000	313460000	371563333.3	247923333.3	0.032173898	0.667243808	-0.583714083
Q9H2W6	MRPL46	mL46	764050000	544010000	647540000	463300000	396750000	451140000	651866666.7	437063333.3	0.03236917	0.670479648	-0.576734554
Q13405	MRPL49	mL49	1614300000	1919300000	2357700000	1290200000	1397600000	1305600000	1963766667	1331133333	0.044229836	0.677846995	-0.560968433
Q8N5N7	MRPL50	mL50	840890000	787160000	707220000	368180000	331300000	480320000	778423333.3	393266666.7	0.002897387	0.505209248	-0.985047047
Q96EL3	MRPL53	mL53	490670000	474820000	414310000	213580000	311340000	203200000	459933333.3	242706666.7	0.006402104	0.527699667	-0.922211023
Q6P161	MRPL54	mL54	48283000	102660000	99356000	0	0	0	83433000	0	0.009035338	0	N/A
O60783	MRPS14	uS14m	231980000	321570000	311630000	385400000	373210000	361700000	288393333.3	373436666.7	0.043424842	1.294886613	0.372825774
P82650	MRPS22	mS22	682610000	649730000	763700000	480900000	564240000	393620000	698680000	479586666.7	0.021482744	0.686418198	-0.542840293
Q9Y3D9	MRPS23	mS23	818430000	778220000	643480000	1090400000	1109100000	1377200000	746710000	1192233333	0.013961503	1.596648409	0.675046658
P82663	MRPS25	mS25	557260000	456520000	452480000	165350000	203650000	235440000	488753333.3	201480000	0.001956502	0.412232483	-1.278469906
Q9NP92	MRPS30	mS30	0	82371000	0	115500000	105660000	147240000	27457000	122800000	0.034237845	4.472447828	2.161064653
P82930	MRPS34	mS34	582910000	633270000	560950000	446590000	401260000	511570000	592376666.7	453140000	0.022444316	0.76495248	-0.386557968
Q96E11	MRRF		194170000	182330000	222610000	136520000	123440000	99606000	199703333.3	119855333.3	0.007730743	0.600166914	-0.736564306
Q5JTZ9	AARS2		317730000	380700000	366390000	216830000	295750000	264240000	354940000	258940000	0.032303242	0.729531752	-0.454957324
Q96RP9	GFM1		848400000	796420000	810510000	459330000	405400000	300520000	818443333.3	388416666.7	0.000939749	0.474579792	-1.075277425
Q969S9	GFM2		169210000	103840000	81366000	46736000	0	0	118138666.7	15578666.67	0.028548997	0.131867636	-2.922837567
Q9NP81	SARS2		902320000	694570000	629120000	187300000	244000000	290300000	742003333.3	240533333.3	0.004604467	0.324167457	-1.62518883
Q9BYD6	MRPL1	uL1m	1073000000	929590000	1040400000	864540000	931340000	1303300000	1014330000	1033060000	0.902267104	1.018465391	0.026396956
Q5T653	MRPL2	uL2m	293480000	301280000	224920000	395420000	287810000	207310000	273226666.7	296846666.7	0.712317747	1.08644837	0.119619617
P09001	MRPL3	uL3m	671180000	614510000	723970000	737100000	660110000	372780000	669886666.7	589996666.7	0.526425981	0.880741021	-0.183210233
Q7Z7H8	MRPL10	uL10m	552970000	186290000	362910000	212690000	158650000	79051000	367390000	150130333.3	0.126300114	0.408640228	-1.29109686
P52815	MRPL12	bL12m	5001100000	3828300000	4883400000	3073300000	4093000000	2206900000	4570933333	3124400000	0.093652099	0.68353655	-0.548909612
Q9NX20	MRPL16	uL16m	134310000	169170000	135220000	126120000	95485000	102670000	146233333.3	108091666.7	0.060790379	0.739172555	-0.436016903
Q9NRX2	MRPL17	bL17m	433920000	370280000	369210000	340760000	402690000	393060000	391136666.7	378836666.7	0.691034817	0.968553191	-0.046096814
Q9H0U6	MRPL18	uL18m	154500000	0	173910000	244190000	209860000	0	109470000	151350000	0.679257366	1.382570567	0.467353118
Q9BYC9	MRPL20	bL20m	200570000	164530000	192990000	130900000	168480000	0	186030000	99793333.33	0.174047848	0.536436775	-0.89851995
Q7Z2W9	MRPL21	bL21m	359590000	275590000	204880000	138910000	215450000	218840000	280020000	191066666.7	0.160905635	0.682332214	-0.551453764
Q9NWU5	MRPL22	uL22m	84671000	109920000	115630000	97380000	93406000	0	103407000	63595333.33	0.296770179	0.615000274	-0.701341042
Q16540	MRPL23	uL23m	445490000	535820000	511940000	272900000	387810000	909430000	497750000	523380000	0.903113241	1.051491713	0.07243748
Q96A35	MRPL24	uL24m	279200000	351710000	481510000	230140000	292180000	233200000	370806666.7	251840000	0.129839726	0.67916794	-0.558159736
Q9P0M9	MRPL27	bL27m	211720000	163500000	222360000	287220000	206180000	121650000	199193333.3	205016666.7	0.914785425	1.029234579	0.041571834
Q13084	MRPL28	bL28m	1272900000	1029200000	914890000	773910000	878590000	638110000	1072330000	763536666.7	0.071068177	0.712035163	-0.489979606
Q8TCC3	MRPL30	uL30m	0	33038000	49729000	77887000	48520000	0	27589000	42135666.67	0.618686343	1.527263281	0.610948786
Q9BYC8	MRPL32	bL32m	65201000	0	0	140120000	67181000	0	21733666.67	69100333.33	0.360665045	3.179414426	1.668761079
O75394	MRPL33	bL33m	73163000	0	107990000	142180000	122410000	88178000	60384333.33	117589333.3	0.182549826	1.947348374	0.961511
Q9NZE8	MRPL35	bL35m	0	112440000	0	111650000	104540000	0	37480000	72063333.33	0.54262392	1.922714337	0.943144433
Q9BZE1	MRPL37	mL37	660990000	579870000	857640000	704220000	658610000	780970000	699500000	714600000	0.874708227	1.021586848	0.030811857
Q96DV4	MRPL38	mL38	250170000	198760000	199310000	250240000	199850000	257010000	216080000	235700000	0.473350749	1.090799704	0.125386214
Q9NYK5	MRPL39	mL39	464970000	508850000	437570000	369110000	430020000	438280000	470463333.3	412470000	0.126395232	0.876731449	-0.189793095
Q9NQ50	MRPL40	mL40	525010000	446470000	514070000	381520000	553520000	531930000	495183333.3	488990000	0.921990659	0.987492848	-0.018157796
Q9Y6G3	MRPL42	mL42	630490000	448430000	527370000	289180000	369760000	0	535430000	219646666.7	0.063557463	0.410224804	-1.28551337
Q8N983	MRPL43	mL43	744820000	943170000	1148900000	1076800000	1149000000	717720000	945630000	981173333.3	0.850798074	1.037586935	0.053232219

Q9HD33	MRPL47	uL29m	355490000	367860000	426450000	215330000	259810000	430380000	383266666.7	301840000	0.303940263	0.78754566	-0.344564525	
Q96GC5	MRPL48	mL48	254610000	0	291870000	304510000	335450000	0	182160000	213320000	0.835865285	1.17105841	0.227813037	
Q7Z7F7	MRPL55	bL31m	183800000	180090000	131530000	0	148370000	132730000	165140000	93700000	0.226176647	0.56739736	-0.817568657	
Q9BQC6	MRPL57	mL63	349220000	341170000	0	0	0	267660000	230130000	89220000	0.388016587	0.387693912	-1.367010014	
Q14197	MRPL58	mL62	0	611560000	425640000	272490000	300100000	289530000	345733333.3	287373333.3	0.763507207	0.831199383	-0.266733512	
Q9Y399	MRPS2	uS2m	51786000	0	16587000	0	0	0	22791000	0	0.209789008	0	N/A	
P82675	MRPS5	uS5m	118960000	158080000	167400000	0	105150000	68430000	148146666.7	57860000	0.057574024	0.390558906	-1.356387937	
P82932	MRPS6	bS6m	310710000	326440000	374640000	228520000	206880000	328900000	337263333.3	254766666.7	0.122421705	0.755393906	-0.40469895	
Q9Y2R9	MRPS7	uS7m	968460000	1213900000	1097600000	1055200000	1023800000	899960000	1093320000	992986666.7	0.304554415	0.908230588	-0.138869469	
P82933	MRPS9	uS9m	896350000	886050000	721050000	971240000	727430000	603990000	834483333.3	767553333.3	0.612267228	0.919794683	-0.120616236	
P82664	MRPS10	uS10m	0	0	0	55998000	53002000	0	0	36333333.33	0.116416934	N/A	N/A	
P82912	MRPS11	uS11m	180180000	215760000	226160000	293300000	308860000	182920000	207366666.7	261693333.3	0.265628181	1.261983604	0.335693167	
O15235	MRPS12	uS12m	0	89686000	137070000	95115000	118090000	102330000	75585333.33	105178333.3	0.508027898	1.391517755	0.476659317	
Q9Y3D3	MRPS16	bS16m	313750000	329380000	0	199350000	182970000	0	214376666.7	127440000	0.524625625	0.594467681	-0.750329716	
Q9Y2R5	MRPS17	uS17m	858950000	673840000	748840000	477180000	611910000	863280000	760543333.3	650790000	0.430390566	0.855690888	-0.224838368	
Q9NVS2	MRPS18A	mL66	0	0	37370000	55158000	0	0	12456666.67	18386000	0.802679574	1.475996789	0.561689583	
Q9Y676	MRPS18B	mS40	581250000	246670000	95498000	89624000	38419000	274280000	307806000	134107666.7	0.339762362	0.43568893	-1.198629639	
Q9Y3D5	MRPS18C	bS18m	101910000	0	0	0	147490000	104060000	33970000	83850000	0.418813081	2.46835443	1.303549566	
Q96EL2	MRPS24	mS24	27924000	34500000	35927000	51289000	47624000	0	32783666.67	32971000	0.991588176	1.005714228	0.008220423	
Q9BYN8	MRPS26	mS26	81306000	74888000	124140000	97460000	89872000	0	93444666.67	62444000	0.424685468	0.668245735	-0.581549369	
Q92552	MRPS27	mS27	348430000	171940000	458840000	184090000	210750000	232910000	326403333.3	209250000	0.238963169	0.641078012	-0.641428168	
Q9Y2Q9	MRPS28	mS28	518380000	546800000	173090000	0	129320000	350840000	412756666.7	160053333.3	0.184681158	0.387766804	-1.366738794	
Q92665	MRPS31	mS31	517530000	800300000	659730000	703360000	662420000	583800000	659186666.7	649860000	0.921450257	0.985851251	-0.020558111	
P82673	MRPS35	mS35	406820000	504580000	380240000	387470000	395710000	276860000	430546666.7	353346666.7	0.224815408	0.820693072	-0.28508532	
P82909	MRPS36	mS36	220070000	240470000	422160000	171170000	139980000	0	294233333.3	103716666.7	0.083473865	0.352498017	-1.504312951	
A4D1E9	GTPBP10		0	0	0	70904000	47299000	0	0	39401000	0.131731208	N/A	N/A	
Q96EH3	MALSU1		272760000	96853000	124990000	142910000	43343000	0	164867666.7	62084333.33	0.21075968	0.376570704	-1.409007327	
Q6IN84	MRM1		214640000	397100000	313280000	70764000	154390000	428130000	308340000	217761333.3	0.492740697	0.706237703	-0.501774253	
Q9HC36	MRM3		115680000	48854000	153570000	0	0	0	106034666.7	0	0.025725708	0	N/A	
O75616	ERAL1		185710000	139490000	160130000	104990000	86479000	189790000	161776666.7	127086333.3	0.371528871	0.785566522	-0.348194647	
Q9NYY8	FASTKD2		58120000	0	92643000	115940000	54180000	0	50254333.33	56706666.67	0.888087921	1.128393571	0.174270352	
Q96E29	MTERF3		46495000	141980000	0	0	0	0	62825000	0	0.207191311	0	N/A	
Q9BSH4	TACO1		786100000	726350000	709350000	552650000	672970000	578670000	740600000	601430000	0.032533069	0.812084796	-0.300297717	
Q9BVS5	TRMT61B		147300000	106460000	145920000	54082000	0	0	133226666.7	18027333.33	0.006838137	0.135313251	-2.885624972	
Q9HA77	CARS2		0	63087000	0	0	56116000	0	21029000	18705333.33	0.938166089	0.889501799	-0.168930571	
Q96SZ6	CDK5RAP1		181680000	122240000	0	0	0	0	101306666.7	0	0.131103414	0	N/A	
Q6PI48	DARS2		683390000	715360000	718970000	547800000	565840000	760410000	705906666.7	624683333.3	0.304370283	0.884937574	-0.176352407	
Q5JPH6	EARS2		0	0	40572000	0	0	48503000	13524000	16167666.67	0.906240785	1.195479641	0.257589561	
Q969Y2	GTPBP3		38668000	23395000	0	0	9575500	56273000	20687666.67	21949500	0.954322619	1.060994473	0.085417141	
Q8N442	GUF1		58279000	173100000	65877000	142170000	127920000	0	99085333.33	90030000	0.884404525	0.90861076	-0.138265705	
P49590	HARS2		308180000	156050000	272450000	248860000	151750000	160430000	245560000	187013333.3	0.350395676	0.761578976	-0.392934443	
Q15031	LARS2		345630000	280610000	755310000	253450000	262150000	0	460516666.7	171866666.7	0.167964721	0.37320401	-1.421963607	
Q96GW9	MARS2		318540000	247860000	217380000	135440000	242130000	281550000	261260000	219706666.7	0.476365879	0.840950267	-0.249907612	
P46199	MTIF2		109670000	93887000	98694000	0	0	0	100750333.3	0	2.73217E-05	0	N/A	
Q96I59	NARS2		148470000	127430000	83803000	66713000	101720000	120580000	119901000	96337666.67	0.394681192	0.803476757	-0.315671804	
Q7L3T8	PARS2		0	0	0	20620000	17433000	0	0	12684333.33	0.118899056	N/A	N/A	
Q9H0R6	QRSL1		69998000	63972000	0	0	41116000	0	44656666.67	13705333.33	0.303811006	0.306904531	-1.70413815	
Q8N0V3	RBFA		77521000	0	149450000	60846000	12877000	84770000	75657000	52831000	0.659537691	0.698296258	-0.518088853	
Q9BW92	TARS2		316610000	258310000	298330000	346570000	613260000	530680000	291083333.3	496836666.7	0.063294759	1.706853707	0.771339412	
Q7L0Y3	TRMT10C		609870000	571420000	574930000	360900000	496680000	531180000	585406666.7	462920000	0.083521412	0.790766533	-0.338676281	
P43897	TSFM		864190000	747260000	753540000	632830000	563110000	914380000	788330000	703440000	0.497464716	0.892316669	-0.164372304	
P49411	TUFM		8798400000	9533400000	7836700000	10559000000	9557400000	7295000000	8722833333	9137133333	0.721557611	1.047496035	0.066944783	
Q5ST30	VARS2		208500000	179640000	234170000	207300000	242440000	141210000	207436666.7	196983333.3	0.771224442	0.949607109	-0.074597359	
Q9Y2Z4	YARS2		1439900000	1854300000	1423500000	1094600000	1248300000	1670800000	1572566667	1337900000	0.351207963	0.850774742	-0.233150892	
Q9UDR5	AASS		0	0	0	56920000	101220000	0	0	52713333.33	0.146344196	N/A	N/A	
P80404	ABAT		0	0	122840000	55841000	82771000	85445000	40946666.67	74685666.67	0.467052392	1.823974275	0.867085383	
Q9NRK6	ABCB10		108600000	45671000	48813000	53176000	38319000	0	67694666.67	30498333.33	0.224094027	0.45052786	-1.150311771	

O75027	ABCB7		135340000	140360000	134300000	167340000	181670000	47261000	136666666.7	132090333.3	0.919730696	0.966514634	-0.049136518	
Q9NUJ1	ABHD10		130020000	156930000	194110000	142060000	127430000	141600000	160353333.3	137030000	0.291059736	0.854550368	-0.226762567	
P42765	ACAA2		2054300000	2106600000	2005000000	2069700000	1711300000	1660200000	2055300000	1813733333	0.14151012	0.882466469	-0.180386634	
Q9H845	ACAD9		703850000	637860000	744580000	634920000	553530000	605910000	695430000	598120000	0.067881742	0.860072186	-0.217470345	
P11310	ACADM		2851600000	3254600000	4051800000	3464000000	4167500000	2954100000	3386000000	3528533333	0.788964094	1.042094901	0.059486666	
P16219	ACADS		491180000	240450000	381450000	152530000	260980000	172840000	371026666.7	195450000	0.092735203	0.526781543	-0.924723296	
P45954	ACADSB		225330000	161080000	188090000	105870000	186900000	0	191500000	97590000	0.176144311	0.509608355	-0.972539164	
P49748	ACADVL		4016100000	3359300000	3560300000	2943500000	3111900000	3592200000	3645233333	3215866667	0.19322764	0.882211473	-0.180803573	
P24752	ACAT1		4453400000	4306100000	4534000000	3698100000	3553400000	2944100000	4431166667	3398533333	0.01270285	0.766961297	-0.382774318	
Q9Y305	ACOT9		61697000	75601000	73970000	0	93678000	0	70422666.67	31226000	0.28173652	0.443408372	-1.173292084	
Q96CM8	ACSF2		269830000	277490000	232090000	111850000	166570000	146060000	259803333.3	141493333.3	0.005099772	0.544617082	-0.876685861	
Q4G176	ACSF3		0	0	0	14880000	0	89700000	0	34860000	0.277467671	N/A	N/A	
Q9H6R3	ACSS3		0	0	0	29632000	67900000	0	0	32510666.67	0.173436757	N/A	N/A	
Q53H12	AGK		116130000	82546000	0	102500000	25704000	108150000	66225333.33	78784666.67	0.787423943	1.189645453	0.250531675	
P54819	AK2		1775200000	1662600000	1114700000	1390300000	1196700000	1614500000	1517500000	1400500000	0.647479149	0.922899506	-0.115754533	
Q9UIJ7	AK3		661070000	498690000	441250000	296060000	455790000	437570000	533670000	396473333.3	0.173484489	0.742918533	-0.428724079	
P27144	AK4		808650000	721600000	997570000	618380000	703200000	624440000	842606666.7	648673333.3	0.086940402	0.769841207	-0.377367199	
Q92667	AKAP1		138150000	125370000	0	172920000	186280000	108160000	87840000	155786666.7	0.247672379	1.773527626	0.826621803	
P30837	ALDH1B1		3289500000	3096600000	3384800000	1513900000	1670900000	1844800000	3256966667	1676533333	0.000245321	0.514752991	-0.958047787	
P05091	ALDH2		1676800000	1489600000	1688600000	0	0	39348000	1618333333	13116000	1.67295E-05	0.008104634	-6.947037178	
P30038	ALDH4A1		228930000	176370000	201460000	206950000	205230000	175090000	202253333.3	195756666.7	0.741426849	0.967878568	-0.047102039	
P51649	ALDH5A1		220860000	204430000	229100000	236330000	259370000	203800000	218130000	233166666.7	0.442855915	1.068934427	0.096173355	
Q02252	ALDH6A1		1037100000	1059900000	1107100000	938210000	1032900000	1100300000	1068033333	1023803333	0.437473954	0.958587435	-0.061018066	
P27695	APEX1		180130000	233280000	287680000	291120000	333020000	453300000	233696666.7	359146666.7	0.095265323	1.536806972	0.619935969	
Q9BUR5	APOO		0	0	0	165260000	134520000	361110000	0	220296666.7	0.036068439	N/A	N/A	
Q6UXV4	APOOL		0	112210000	0	146130000	111630000	84773000	37403333.33	114177666.7	0.137313536	3.05260672	1.610041733	
P78540	ARG2		643860000	614840000	730100000	0	141700000	0	662933333.3	47233333.33	0.000462664	0.071248994	-3.810986538	
P25705	ATP5F1A		32993000000	34874000000	30987000000	28358000000	23819000000	23702000000	32951333333	25293000000	0.015718586	0.767586542	-0.381598678	
P06576	ATP5F1B		46924000000	40118000000	44169000000	30941000000	38099000000	43170000000	43737000000	37403333333	0.193841627	0.855187446	-0.22568742	
P36542	ATP5F1C		2703800000	2394700000	2016800000	2297200000	2249600000	1376600000	2371766667	1974466667	0.330719709	0.832487738	-0.264499073	
P30049	ATP5F1D		812530000	1007300000	393590000	1116400000	847060000	877340000	737806666.7	946933333.3	0.354960865	1.283443721	0.360020035	
P56381	ATP5F1E		42197000	0	33080000	0	0	0	25092333.33	0	0.12191815	0	N/A	
Q9UII2	ATP5IF1		41159000	0	61692000	36766000	10773000	16171000	34283666.67	21236666.67	0.545767587	0.619439772	-0.690964078	
Q96IX5	ATP5MD		232050000	348280000	408850000	422980000	335810000	244630000	329726666.7	334473333.3	0.951335382	1.014395762	0.020620623	
P56385	ATP5ME		770700000	852540000	1115500000	794180000	913160000	550550000	912913333.3	752630000	0.342667291	0.824426561	-0.278537109	
P56134	ATP5MF		286510000	364110000	441740000	576390000	563640000	954640000	364120000	698223333.3	0.069758443	1.917563807	0.939274584	
O75964	ATP5MG		1174300000	1201400000	1110300000	1267600000	1432200000	1306100000	1162000000	1335300000	0.037534692	1.149139415	0.200553838	
P24539	ATP5PB		1348000000	1655200000	1597800000	2802100000	2003600000	1288900000	1533666667	2031533333	0.32787766	1.324625082	0.405584081	
O75947	ATP5PD		3511300000	3727700000	3620300000	1264100000	1500100000	2811200000	3619766667	1858466667	0.022166482	0.513421675	-0.961783891	
P18859	ATP5PF		1580300000	2210500000	2457200000	2577300000	2268200000	2220200000	2082666667	2355233333	0.391612143	1.13087388	0.177438042	
P48047	ATP5PO		7121400000	7438800000	7656800000	5207100000	4999500000	5337700000	7405666667	5181433333	0.000268662	0.69965792	-0.51527837	
Q8N5M1	ATPAF2		48377000	32677000	58180000	0	31319000	0	46411333.33	10439666.67	0.048434756	0.224937874	-2.152401497	
Q13825	AUH		0	105720000	92271000	0	0	0	65997000	0	0.117948586	0	N/A	
P12694	BCKDHA		0	0	190940000	170670000	0	0	63646666.67	56890000	0.940715373	0.893840997	-0.161909878	
P21953	BCKDHB		31200000	0	114330000	170850000	149290000	187020000	48510000	169053333.3	0.028186244	3.484917199	1.801124378	
Q9Y276	BCS1L		98517000	95582000	35866000	67697000	18780000	53585000	76655000	46687333.33	0.297785851	0.6090579	-0.71534871	
Q07021	C1QBP		4989500000	4441300000	5013900000	2529100000	3575000000	3875000000	4814900000	3326366667	0.029453689	0.690848547	-0.533558629	
Q96ER9	CCDC51		529290000	515960000	547240000	414290000	562250000	468770000	530830000	481770000	0.328712126	0.907578698	-0.139905348	
Q96BP2	CHCHD1		120890000	119330000	0	128570000	135990000	144100000	80073333.33	136220000	0.235884567	1.701190575	0.766544767	
Q9NX63	CHCHD3		288490000	342740000	432500000	546740000	293560000	124380000	354576666.7	321560000	0.811628537	0.906884266	-0.141009645	
Q9BRQ6	CHCHD6		159380000	175230000	174270000	372830000	265840000	168440000	169626666.7	269036666.7	0.168669222	1.586051721	0.665439818	
P0C7P0	CISD3		48513000	48666000	0	0	0	0	32393000	0	0.116117509	0	N/A	
P12532	CKMT1A		865540000	762420000	987800000	351670000	321890000	337860000	871920000	337140000	0.001239851	0.386663914	-1.370847965	
Q16740	CLPP		314990000	284030000	0	228630000	221320000	0	199673333.3	149983333.3	0.711720351	0.751143534	-0.412839479	
O76031	CLPX		971810000	973110000	1044200000	654820000	627440000	561380000	996373333.3	614546666.7	0.00047803	0.616783535	-0.697163842	
Q7Z7K0	CMC1		28531000	31789000	35186000	0	0	0	31835333.33	0	7.76958E-05	0	N/A	
Q96BR5	COA7		134680000	138210000	142260000	151640000	119430000	127460000	138383333.3	132843333.3	0.606469062	0.959966277	-0.058944369	

Q9Y2Z9	COQ6		119330000	149530000	122870000	147500000	127040000	153950000	130576666.7	142830000	0.383004629	1.093840145	0.129401916	
Q96D53	COQ8B		161060000	116270000	98535000	93092000	115520000	152380000	125288333.3	120330666.7	0.854725066	0.960429942	-0.058247714	
O75208	COQ9		138260000	130200000	187090000	289300000	235070000	187150000	151850000	237173333.3	0.0684303	1.561892218	0.643294901	
Q14061	COX17		45114000	51556000	0	0	0	0	32223333.33	0	0.11787987	0	N/A	
Q5RI15	COX20		934260000	1208300000	1007700000	670050000	521640000	725440000	1050086667	639043333.3	0.015747989	0.608562468	-0.716522734	
P13073	COX4I1		617810000	1010000000	1851700000	1473900000	823400000	492340000	1159836667	929880000	0.646404731	0.801733577	-0.318805198	
P20674	COX5A		4302600000	5017700000	5169100000	2277300000	2217400000	2149900000	4829800000	2214866667	0.00063354	0.458583516	-1.124743597	
P10606	COX5B		1934600000	2614000000	3079700000	3659700000	2808400000	1621700000	2542766667	2696600000	0.831649215	1.060498407	0.084742454	
P12074	COX6A1		646860000	613950000	919130000	2015500000	1417000000	1177300000	726646666.7	1536600000	0.038798037	2.114645357	1.080415732	
P14854	COX6B1		951720000	1202000000	1961400000	1750100000	1822800000	2617500000	1371706667	2063466667	0.16804491	1.504306071	0.589098133	
P09669	COX6C		56552000	43309000	85903000	74069000	32642000	15229000	61921333.33	40646666.67	0.378737672	0.65642428	-0.607299492	
P14406	COX7A2		503740000	0	0	650760000	714770000	0	167913333.3	455176666.7	0.368134193	2.710783341	1.43870981	
O14548	COX7A2L		166720000	136150000	541470000	469460000	346740000	400390000	281446666.7	405530000	0.410240719	1.440876898	0.526947083	
P15954	COX7C		193780000	139450000	39677000	152170000	171110000	275330000	124302333.3	199536666.7	0.272531356	1.605252784	0.682800501	
P36551	CPOX		314900000	319870000	357270000	289270000	355470000	177800000	330680000	274180000	0.350798727	0.829139954	-0.270312454	
P31327	CPS1		163910000	0	68440000	0	55999000	0	77450000	18666333.33	0.313795635	0.241011405	-2.052826675	
P23786	CPT2		98522000	83377000	110030000	0	0	0	97309666.67	0	0.000227786	0	N/A	
O75390	CS		2720500000	2756000000	3017000000	672950000	792790000	1273800000	2831166667	913180000	0.000740923	0.322545476	-1.632425513	
P00167	CYB5A		410020000	488810000	642960000	592880000	562850000	341030000	513930000	498920000	0.893056689	0.970793688	-0.042763367	
P00387	CYB5R3		795800000	597130000	797210000	1151700000	1134700000	1099700000	730046666.7	1128700000	0.004271531	1.546065548	0.628601486	
P08574	CYC1		3224800000	3103900000	3098100000	3660700000	4160600000	3736700000	3142266667	3852666667	0.011557231	1.226078839	0.29405175	
Q8N465	D2HGDH		0	0	0	0	48341000	72377000	0	40239333.33	0.131640555	N/A	N/A	
P51398	DAP3		1271900000	1349600000	1386200000	951530000	910720000	926530000	1335900000	929593333.3	0.000341106	0.695855478	-0.52314039	
P11182	DBT		178110000	215290000	170770000	255060000	287710000	176280000	188056666.7	239683333.3	0.223047405	1.274527182	0.349962142	
Q16698	DECR1		476300000	456850000	376810000	0	185620000	243690000	436653333.3	143103333.3	0.021012433	0.327727564	-1.609431076	
Q7Z3D6	DGLUCY		64381000	73662000	0	0	0	0	46014333.33	0	0.11791137	0	N/A	
Q02127	DHODH		237920000	244130000	222270000	81057000	68338000	37789000	234773333.3	62394666.67	0.000278495	0.265765561	-1.911773928	
Q13268	DHRS2		15551000000	14376000000	13670000000	6334100000	6090600000	7069700000	14532333333	6498133333	0.000207919	0.447150033	-1.161169112	
Q9NR28	DIABLO		211010000	200920000	184750000	173310000	237300000	0	198893333.3	136870000	0.433421022	0.688157807	-0.539188657	
P10515	DLAT		1526600000	1231800000	1471800000	715830000	934530000	1093000000	1410066667	914453333.3	0.025090958	0.648517801	-0.624781921	
P09622	DLD		2317400000	2642500000	2926000000	281060000	186340000	207230000	2628633333	224876666.7	0.000174605	0.085548891	-3.547107036	
P36957	DLST		177150000	163220000	0	0	0	0	113456666.7	0	0.116782308	0	N/A	
Q96EY1	DNAJA3		454020000	402090000	489070000	207050000	313860000	277740000	448393333.3	266216666.7	0.01063356	0.593712366	-0.752163934	
Q96DA6	DNAJC19		3077100000	1628100000	1382100000	369740000	679080000	623400000	2029100000	557406666.7	0.051953783	0.274706356	-1.864037805	
O00429	DNM1L		216550000	224950000	217320000	327450000	236240000	432740000	219606666.7	332143333.3	0.118800391	1.512446495	0.596884106	
P33316	DUT		215130000	0	228180000	122880000	160150000	171140000	147770000	151390000	0.964013418	1.02449753	0.034916506	
Q13011	ECH1		29478000	15506000	40334000	50669000	21115000	0	28439333.33	23928000	0.796364781	0.841369934	-0.249187831	
P30084	ECHS1		5409500000	6963400000	5671600000	4442900000	3127700000	2805400000	6014833333	3458666667	0.021134464	0.57502286	-0.798308783	
P42126	ECI1		0	0	200510000	0	54165000	0	66836666.67	18055000	0.519916165	0.270136153	-1.888241364	
Q9BQ95	ECSIT		206650000	296940000	107510000	69316000	69085000	0	203700000	46133666.67	0.056740933	0.226478481	-2.142554114	
P13804	ETFA		5520600000	5047100000	4964200000	2674500000	3145400000	4480300000	5177300000	3433400000	0.037265208	0.663164198	-0.592561973	
O95571	ETHE1		511710000	917850000	1119800000	933530000	868900000	883540000	849786666.7	895323333.3	0.812626288	1.053585998	0.075308078	
Q6P587	FAHD1		205270000	217250000	136540000	144340000	134450000	175770000	186353333.3	151520000	0.282313363	0.813079097	-0.298532389	
Q6P4F2	FDX2		43486000	0	42470000	0	0	0	28652000	0	0.116172091	0	N/A	
P22570	FDXR		2955200000	3080300000	3640800000	4840600000	4387800000	3793400000	3225433333	4340600000	0.039174255	1.345741657	0.428401482	
P22830	FECH		218270000	120520000	226990000	0	0	0	188593333.3	0	0.005238875	0	N/A	
P07954	FH		3031700000	3177600000	3079100000	2179600000	2280400000	2762100000	3096133333	2407366667	0.020349355	0.777539727	-0.363011706	
Q9Y3D6	FIS1		0	0	103880000	0	66598000	84507000	34626666.67	50368333.33	0.733589004	1.45461109	0.54063348	
Q8TAE8	GADD45GIP1		571460000	606160000	602300000	338860000	337310000	355450000	593306666.7	343873333.3	3.62851E-05	0.579587847	-0.786900751	
P0DPI2	GATD3A		507990000	452020000	461230000	562510000	442720000	239000000	473746666.7	414743333.3	0.572076143	0.875453829	-0.191897001	
Q86SX6	GLRX5		2000800000	1982500000	1778100000	266080000	375950000	320600000	1920466667	320876666.7	3.35767E-05	0.167082654	-2.581366134	
O94925	GLS		5598300000	4943700000	5403300000	3923200000	3934000000	4207300000	5315100000	4021500000	0.0038535	0.756617938	-0.402363115	
P00367	GLUD1		5338600000	6295300000	5904600000	3854100000	3676400000	3404700000	5846166667	3645066667	0.002001769	0.623496878	-0.681545758	
Q9H4A6	GOLPH3		208470000	163930000	258140000	153650000	208750000	151130000	210180000	171176666.7	0.303628324	0.814428902	-0.296139335	
P43304	GPD2		2162100000	2332500000	1930700000	1216300000	1196200000	1090300000	2141766667	1167600000	0.001367738	0.545157425	-0.875255199	
Q9HAV7	GRPEL1		2299400000	2131200000	2458600000	2009600000	1768400000	1242300000	2296400000	1673433333	0.064121874	0.728720316	-0.456562883	
Q8TAA5	GRPEL2		193630000	260350000	244390000	0	0	0	232790000	0	0.000318448	0	N/A	

Q16836	HADH	809530000	411850000	667440000	409230000	420960000	405180000	629606666.7	411790000	0.134743726	0.654043265	-0.612542021
P40939	HADHA	10847000000	11373000000	10748000000	10512000000	12413000000	12978000000	10989333333	11967666667	0.273119478	1.089025722	0.12303803
P55084	HADHB	5136200000	5497000000	4821100000	6723100000	6626500000	6878200000	5151433333	6742600000	0.001585398	1.308878435	0.38833111
P31937	HIBADH	1693000000	1461400000	1314200000	992100000	1209500000	1444200000	1489533333	1215266667	0.183737688	0.815870743	-0.293587489
Q6NVY1	HIBCH	201910000	290380000	209490000	116610000	96819000	39691000	233926666.7	84373333.33	0.014904942	0.360682835	-1.471197328
Q9BW72	HIGD2A	1095600000	708180000	497930000	52448000	146000000	462610000	767236666.7	220352666.7	0.06339088	0.287202993	-1.799857313
Q9BX68	HINT2	1457100000	1063800000	1671800000	868120000	1093500000	845850000	1397566667	935823333.3	0.076795049	0.669609082	-0.578608998
P35914	HMGCL	46890000	60343000	0	92188000	107190000	54825000	35744333.33	84734333.33	0.110985381	2.370566896	1.245232106
Q99714	HSD17B10	9046600000	8625900000	8663400000	7041200000	8580600000	9874300000	8778633333	8498700000	0.752820482	0.968111969	-0.04675418
P38646	HSPA9	31444000000	28741000000	28631000000	18474000000	20944000000	21299000000	29605333333	20239000000	0.001849406	0.683626824	-0.548719089
P10809	HSPD1	87661000000	79291000000	71589000000	55788000000	58320000000	65331000000	79513666667	59813000000	0.022438752	0.752235465	-0.41074377
P61604	HSPE1	21309000000	20659000000	23798000000	14659000000	14743000000	12960000000	21922000000	14120666667	0.00222654	0.644132226	-0.634571222
O43464	HTRA2	0	0	183180000	75231000	94193000	139740000	61060000	103054666.7	0.547486315	1.687760673	0.755110342
Q9NSE4	IARS2	2087500000	1933500000	2092500000	3078600000	3016400000	2278700000	2037833333	2791233333	0.045285115	1.369706388	0.453866668
Q5T440	IBA57	684310000	682030000	620270000	491550000	522640000	482810000	662203333.3	499000000	0.002523641	0.753544984	-0.408234457
P48735	IDH2	293000000	235770000	277650000	213580000	149900000	242510000	268806666.7	201996666.7	0.107115799	0.751457057	-0.412237433
P50213	IDH3A	857290000	732990000	704290000	633620000	670070000	789920000	764856666.7	697870000	0.371322614	0.91241932	-0.132231099
O43837	IDH3B	146900000	100170000	143980000	156870000	192470000	144390000	130350000	164576666.7	0.176486764	1.262575118	0.336369225
P51553	IDH3G	0	85738000	0	90118000	80299000	0	28579333.33	56805666.67	0.523171446	1.987648417	0.99106259
Q16891	IMMT	4099900000	4769900000	4897300000	4681600000	4462200000	3329700000	4589033333	4157833333	0.425463496	0.906036856	-0.142358357
Q86U28	ISCA2	365330000	373800000	343410000	156790000	205000000	277580000	360846666.7	213123333.3	0.015164257	0.590620208	-0.759697376
Q9H1K1	ISCU	0	72101000	0	0	0	64127000	24033666.67	21375666.67	0.93810919	0.88940514	-0.169087353
P26440	IVD	95438000	181480000	129270000	290320000	187430000	105700000	135396000	194483333.3	0.373150808	1.436403833	0.522461408
Q9H9P8	L2HGDH	200550000	169740000	123380000	156870000	81821000	196330000	164556666.7	145007000	0.653628405	0.881197966	-0.182461929
P83111	LACTB	1270100000	824260000	639460000	714050000	758510000	642630000	911273333.3	705063333.3	0.33925473	0.773712241	-0.370130995
O95202	LETM1	1551800000	1448500000	1837700000	1115000000	1150700000	1184300000	1612666667	1150000000	0.017286875	0.713104589	-0.487814407
P36776	LONP1	2559400000	2373700000	2193600000	1816600000	2135800000	2333900000	2375566667	2095433333	0.202569189	0.882077259	-0.181023072
P42704	LRPPRC	15714000000	14870000000	14931000000	8381500000	8612700000	9205800000	15171666667	8733333333	6.14638E-05	0.575634406	-0.796775269
Q8WWC4	MAIP1	0	215060000	175930000	0	0	82820000	130330000	27606666.67	0.225053411	0.211821274	-2.239080602
Q9NX47	MARCHF5	0	0	0	55381000	64631000	0	0	40004000	0.118474101	N/A	N/A
Q8IVS2	MCAT	156610000	227400000	144950000	103110000	69796000	173310000	176320000	115405333.3	0.201812577	0.654522081	-0.611486231
Q96RQ3	MCCC1	124670000	0	88954000	40777000	102780000	0	71208000	47852333.33	0.64943396	0.672007827	-0.573450059
Q9HCC0	MCCC2	692490000	528710000	867820000	1010200000	940390000	889690000	696340000	946760000	0.07364385	1.359623173	0.443206856
Q96PE7	MCEE	0	0	0	118650000	74729000	0	0	64459666.67	0.136216592	N/A	N/A
Q8NE86	MCU	186680000	226180000	187200000	81767000	149240000	166380000	200020000	132462333.3	0.079935523	0.662245442	-0.594562086
P40926	MDH2	34905000000	31124000000	34717000000	24425000000	22772000000	24022000000	33582000000	23739666667	0.001763612	0.706916404	-0.500388476
P23368	ME2	2125400000	1865400000	1255100000	698040000	840650000	1122200000	1748633333	886963333.3	0.039626163	0.507232315	-0.979281435
Q9GZY8	MFF	97963000	0	124790000	0	0	0	74251000	0	0.121853662	0	N/A
Q8IWA4	MFN1	302740000	275140000	179030000	142990000	147740000	337110000	252303333.3	209280000	0.592704095	0.829477745	-0.269724821
O95140	MFN2	297850000	261470000	258230000	414710000	362410000	170500000	272516666.7	315873333.3	0.595681922	1.159097303	0.213001682
Q9BQP7	MGME1	330980000	322380000	297960000	175860000	169890000	161060000	317106666.7	168936666.7	0.000162567	0.532743977	-0.908485718
Q5XKP0	MICOS13	0	78087000	53012000	41516000	0	0	43699666.67	13838666.67	0.328527839	0.31667671	-1.658917327
Q9BPX6	MICU1	128730000	90958000	116780000	0	69637000	0	112156000	23212333.33	0.02595588	0.206964704	-2.272543346
Q8IYU8	MICU2	26694000	115140000	195820000	0	0	0	112551333.3	0	0.082528835	0	N/A
Q99797	MIPEP	259080000	198180000	131800000	199120000	65527000	150100000	196353333.3	138249000	0.339339033	0.704082776	-0.506183045
P22033	MMUT	373570000	408860000	242860000	118600000	141870000	307370000	341763333.3	189280000	0.122191015	0.55383355	-0.852475645
Q9NZJ7	MTCH1	0	0	74418000	49459000	0	0	24806000	16486333.33	0.793842814	0.664610712	-0.589418548
Q9Y6C9	MTCH2	784000000	437690000	558160000	2775600000	2825000000	1810500000	593283333.3	2470366667	0.00556829	4.163890215	2.057932031
P00403	MT	1326200000	1216400000	1243500000	489730000	651530000	1165800000	1262033333	769020000	0.075347951	0.609349991	-0.714656992
Q9H019	MTFR1L	172090000	235130000	62838000	139870000	167560000	162980000	156686000	156803333.3	0.998276321	1.000748844	0.001079949
Q6UB35	MTHFD1L	5160500000	5017000000	4084800000	2497800000	2914800000	3757300000	4754100000	3056633333	0.027564816	0.64294679	-0.63722875
P13995	MTHFD2	2774000000	2167900000	2936700000	2254100000	2352500000	3074200000	2626200000	2560266667	0.859212749	0.974894017	-0.036682707
Q9NVV4	MTPAP	249810000	0	187620000	0	72173000	0	145810000	24057666.67	0.197412162	0.164993256	-2.599521038
Q13505	MTX1	239000000	240480000	342850000	328470000	333890000	306510000	274110000	322956666.7	0.239492652	1.178200966	0.23658564
O75431	MTX2	1009500000	1573400000	1599800000	768840000	848580000	1627900000	1394233333	1081773333	0.403636734	0.775891171	-0.366073786
Q4G0N4	NADK2	185730000	164220000	181110000	136020000	143120000	0	177020000	93046666.67	0.148691209	0.525627989	-0.927885995
O95299	NDUFA10	349660000	210120000	222480000	135140000	124610000	0	260753333.3	86583333.33	0.048864729	0.332050725	-1.590524447

Q9UI09	NDUFA12		871680000	710700000	910580000	390720000	460590000	355090000	830986666.7	402133333.3	0.003335042	0.483922726	-1.047151401	
Q9P0J0	NDUFA13		289180000	315900000	292470000	155470000	160540000	219610000	299183333.3	178540000	0.005599333	0.596757841	-0.744782478	
O43678	NDUFA2		571340000	489940000	703680000	336990000	376790000	372190000	588320000	361990000	0.023542019	0.615294398	-0.700651238	
Q16718	NDUFA5		1648800000	1556100000	1285400000	908640000	939330000	791800000	1496766667	879923333.3	0.006379077	0.58788277	-0.766399601	
P56556	NDUFA6		139690000	185610000	174240000	276860000	212290000	94542000	166513333.3	194564000	0.637659802	1.168458982	0.22460709	
O95182	NDUFA7		125070000	174020000	263460000	401420000	200490000	109580000	187516666.7	237163333.3	0.629786262	1.264758688	0.33886215	
P51970	NDUFA8		208420000	0	180330000	93810000	98069000	0	129583333.3	63959666.67	0.417853647	0.493579421	-1.018645849	
Q16795	NDUFA9		909280000	953840000	593260000	873220000	752670000	845860000	818793333.3	823916666.7	0.967781816	1.006257175	0.008999071	
Q8N183	NDUFAF2		901490000	709090000	868810000	645540000	852460000	932850000	826463333.3	810283333.3	0.884117939	0.980422604	-0.028524349	
Q7L592	NDUFAF7		71094000	0	96771000	57833000	110760000	0	55955000	56197666.67	0.995780759	1.004336818	0.006243178	
O96000	NDUFB10		311550000	0	341060000	222120000	317950000	245310000	217536666.7	261793333.3	0.714942213	1.20344463	0.267169766	
O95168	NDUFB4		679690000	541040000	711280000	454090000	485390000	628480000	644003333.3	522653333.3	0.180665464	0.811569298	-0.301213806	
O43674	NDUFB5		32282000	0	167200000	0	0	0	66494000	0	0.263916355	0	N/A	
O95169	NDUFB8		0	0	191770000	180930000	217500000	0	63923333.33	132810000	0.499014417	2.077645096	1.054949233	
Q9Y6M9	NDUFB9		183590000	201000000	0	123570000	225700000	279000000	128196666.7	209423333.3	0.361015627	1.633609818	0.708063442	
P28331	NDUFS1		2900400000	2953400000	3188100000	1886400000	2057300000	2228100000	3013966667	2057266667	0.001948849	0.682577777	-0.550934651	
O75306	NDUFS2		1157100000	1044100000	1117400000	893350000	926050000	911540000	1106200000	910313333.3	0.004709851	0.822919303	-0.28117713	
O75489	NDUFS3		1866800000	1781800000	1679300000	972910000	1114500000	1572100000	1775966667	1219836667	0.042128624	0.686857861	-0.541916517	
O43181	NDUFS4		72510000	145140000	183240000	106880000	0	0	133630000	35626666.67	0.111846818	0.2666068	-1.907214518	
O43920	NDUFS5		125210000	228490000	194600000	208010000	178990000	92683000	182766666.7	159894333.3	0.645682007	0.874855006	-0.192884162	
O75380	NDUFS6		126940000	228920000	214770000	279660000	183800000	109150000	190210000	190870000	0.99157615	1.003469849	0.004997269	
O75251	NDUFS7		559210000	0	0	468150000	425970000	602280000	186403333.3	498800000	0.182325216	2.675917813	1.420033806	
O00217	NDUFS8		565440000	353090000	449080000	151760000	160800000	306880000	455870000	206480000	0.034747391	0.452936144	-1.142620425	
P49821	NDUFV1		670690000	929210000	1041300000	627450000	582550000	520670000	880400000	576890000	0.056273887	0.655258973	-0.60986289	
P19404	NDUFV2		758260000	931380000	1011100000	563040000	562360000	868010000	900246666.7	664470000	0.135103566	0.738097707	-0.438116288	
Q9Y697	NFS1		36675000	53341000	31650000	57937000	42004000	0	40555333.33	33313666.67	0.715165718	0.821437378	-0.283777499	
Q9UMS0	NFU1		470180000	495800000	516630000	247860000	232330000	0	494203333.3	160063333.3	0.014717311	0.323881533	-1.626461883	
Q9BYT8	NLN		211460000	170190000	179050000	136310000	191620000	114520000	186900000	147483333.3	0.206232343	0.789102907	-0.34171464	
Q13423	NNT		2331900000	2307800000	2693300000	2698000000	2309200000	2075600000	2444333333	2360933333	0.724141145	0.965880267	-0.050083735	
P80303	NUCB2		927150000	753910000	578060000	503050000	466020000	218010000	753040000	395693333.3	0.056889404	0.525461242	-0.928343741	
Q9BW91	NUDT9		166210000	204610000	213790000	191720000	183270000	234480000	194870000	203156666.7	0.719928311	1.042524076	0.060080701	
P04181	OAT		5759900000	6858700000	5632400000	4048200000	4016200000	4009500000	6083666667	4024633333	0.006140475	0.661547312	-0.596083756	
Q02218	OGDH		1251800000	1225900000	1348400000	1113900000	976890000	1322400000	1275366667	1137730000	0.268273991	0.892080709	-0.164753855	
O60313	OPA1		1585100000	1491900000	1814500000	1445400000	1524000000	1622000000	1630500000	1530466667	0.409175811	0.938648676	-0.091342818	
Q15070	OXA1L		41913000	40111000	37959000	0	0	0	39994333.33	0	3.97966E-06	0	N/A	
P55809	OXCT1		1449800000	1329900000	855740000	670530000	1218200000	1408200000	1211813333	1098976667	0.713317278	0.906886099	-0.141006729	
Q9Y3D7	PAM16		396170000	476070000	385260000	385850000	411970000	146370000	419166666.7	314730000	0.306819689	0.750846918	-0.413409292	
P11498	PC		136780000	354520000	168640000	168110000	214610000	120340000	219980000	167686666.7	0.514172606	0.76228142	-0.391604384	
P05166	PCCB		143650000	157320000	144910000	0	0	0	148626666.7	0	4.42549E-06	0	N/A	
Q16822	PCK2		961930000	839060000	1017400000	563830000	670400000	859560000	939463333.3	697930000	0.075559365	0.742902863	-0.428754509	
Q6L8Q7	PDE12		218360000	151670000	156430000	103600000	0	0	175486666.7	34533333.33	0.025680149	0.196786081	-2.345299918	
Q9HBH1	PDF		0	334750000	73277000	93268000	111990000	205540000	136009000	136932666.7	0.993547996	1.006791217	0.009764536	
P08559	PDHA1		2535700000	2469900000	2376000000	2180600000	2510300000	2636700000	2460533333	2442533333	0.906317882	0.992684513	-0.01059281	
P11177	PDHB		2124500000	2086000000	2040700000	1532000000	1310600000	2130600000	2083733333	1657733333	0.158514492	0.795559253	-0.32995871	
O00330	PDHX		313890000	336600000	370230000	135160000	169790000	0	340240000	101650000	0.011763398	0.298759699	-1.742942545	
Q9P0J1	PDP1		0	0	65031000	112830000	92688000	0	21677000	68506000	0.316601224	3.160308161	1.660065242	
Q8NCN5	PDPR		137520000	77590000	126640000	77807000	148570000	93228000	113916666.7	106535000	0.807147763	0.93520117	-0.096651359	
Q96HS1	PGAM5		810690000	1000200000	1334400000	1272100000	1297000000	784870000	1048430000	1117990000	0.773935281	1.066346823	0.092676743	
Q9UG56	PISD		213200000	153670000	215190000	113660000	97938000	0	194020000	70532666.67	0.039152599	0.363532969	-1.459841886	
Q5JRX3	PITRM1		178910000	172190000	172860000	183650000	114750000	180100000	174653333.3	159500000	0.537555306	0.913237652	-0.130937753	
Q10713	PMPCA		965430000	835310000	999760000	957040000	892790000	778840000	933500000	876223333.3	0.472419847	0.938643099	-0.091351389	
O75439	PMPCB		448500000	280630000	284880000	404690000	383130000	567690000	338003333.3	451836666.7	0.229280473	1.336781689	0.418763876	
Q8TCS8	PNPT1		978560000	905990000	1113900000	865320000	732360000	643620000	999483333.3	747100000	0.04656459	0.747486201	-0.419881148	
P54098	POLG		0	0	0	0	52754000	62324000	0	38359333.33	0.118860052	N/A	N/A	
Q9UHN1	POLG2		0	0	0	0	35192000	31430000	0	22207333.33	0.117383265	N/A	N/A	
P30405	PPIF		2773900000	2123400000	2105700000	1534200000	1634000000	1624100000	2334333333	1597433333	0.02944634	0.684321005	-0.547254863	
P30048	PRDX3		6423700000	4899600000	5564000000	6029400000	7551500000	7647300000	5629100000	7076066667	0.102231231	1.257051157	0.330043363	

P30044	PRDX5		8044200000	6384100000	5384200000	3330100000	3571000000	5045900000	6604166667	3982333333	0.049812317	0.603003155	-0.729762545	
Q96EY7	PTCD3		1133800000	750950000	883300000	831650000	920520000	854190000	922683333.3	868786666.7	0.664735315	0.941587038	-0.086833635	
Q8WUK0	PTPMT1		247360000	218060000	162040000	32995000	43746000	0	209153333.3	25580333.33	0.002903918	0.122304211	-3.031454022	
Q9Y3E5	PTRH2		826720000	774010000	1052600000	837070000	927270000	760850000	884443333.3	841730000	0.685588152	0.951705969	-0.071412175	
P32322	PYCR1		3330900000	2975500000	3142400000	2980600000	2477200000	1974700000	3149600000	2477500000	0.094510973	0.786607823	-0.34628356	
Q8IXI2	RHOT1		112610000	105590000	0	65571000	80818000	72555000	72733333.33	72981333.33	0.994930365	1.003409716	0.004910813	
Q8IXI1	RHOT2		596280000	508800000	448280000	316020000	463230000	424540000	517786666.7	401263333.3	0.131214531	0.774958799	-0.367808484	
P60602	ROMO1		0	0	0	148870000	0	132800000	0	93890000	0.117409604	N/A	N/A	
O75880	SCO1		0	0	492750000	282580000	0	199340000	164250000	160640000	0.985319154	0.978021309	-0.032062196	
P31040	SDHA		3914900000	4472100000	5007000000	3531500000	3197400000	2546200000	4464666667	3091700000	0.032631906	0.692481708	-0.530152132	
P21912	SDHB		2278600000	2495300000	2508700000	1485500000	1415100000	1870700000	2427533333	1590433333	0.006380237	0.655164364	-0.610071207	
P34897	SHMT2		9112100000	7110000000	7494600000	3447200000	3313400000	3416800000	7905566667	3392466667	0.001832749	0.429123782	-1.220534238	
P53007	SLC25A1		503270000	496590000	458120000	329350000	388920000	352210000	485993333.3	356826666.7	0.00444377	0.73422132	-0.445713088	
Q9UBX3	SLC25A10		1253500000	526080000	746910000	72481000	100320000	291010000	842163333.3	154603666.7	0.038319813	0.183579195	-2.445525528	
Q02978	SLC25A11		79011000	124830000	95207000	0	0	0	99682666.67	0	0.001751096	0	N/A	
O75746	SLC25A12		193100000	194340000	169700000	64857000	159980000	255830000	185713333.3	160222333.3	0.671008798	0.862740065	-0.213002139	
Q9UJS0	SLC25A13		2132200000	2104000000	1677300000	1692500000	2281300000	2305400000	1971166667	2093066667	0.649609759	1.061841549	0.086568499	
Q9H936	SLC25A22		324210000	78720000	171970000	133250000	73552000	272970000	191633333.3	159924000	0.749759745	0.834531223	-0.260962068	
Q6NUK1	SLC25A24		1004900000	941210000	981840000	529420000	523900000	579960000	975983333.3	544426666.7	7.46658E-05	0.557823733	-0.842118778	
Q00325	SLC25A3		385920000	540890000	412450000	822170000	713320000	342370000	446420000	625953333.3	0.305487542	1.402162388	0.487653441	
Q9GZT3	SLIRP		3246000000	3306800000	3778400000	3255800000	3215300000	2342700000	3443733333	2937933333	0.213330312	0.853124516	-0.229171772	
Q04837	SSBP1		206800000	464180000	491440000	345990000	491970000	521070000	387473333.3	453010000	0.568552603	1.169138521	0.225445873	
Q9UJZ1	STOML2		4685800000	5196300000	4594300000	4317500000	4277600000	5797600000	4825466667	4797566667	0.960841701	0.994218176	-0.008365617	
Q9P2R7	SUCLA2		260370000	233320000	272340000	286040000	343360000	173410000	255343333.3	267603333.3	0.822644205	1.048013785	0.067657694	
P53597	SUCLG1		1123900000	1129900000	1225800000	984570000	1208700000	805890000	1159866667	999720000	0.256613935	0.861926658	-0.21436298	
Q96I99	SUCLG2		2956500000	2986600000	2715100000	1923200000	2325100000	2159100000	2886066667	2135800000	0.00660663	0.740038345	-0.434328069	
P51687	SUOX		112630000	81026000	21531000	0	0	0	71729000	0	0.054890784	0	N/A	
Q8IYB8	SUPV3L1		123550000	135740000	198850000	185080000	192650000	202480000	152713333.3	193403333.3	0.163497955	1.266446938	0.340786631	
P57105	SYNJ2BP		1047800000	848020000	786900000	1426600000	1135200000	755480000	894240000	1105760000	0.370105859	1.236536053	0.306304304	
Q00059	TFAM		1229400000	1970300000	1807200000	1281500000	1107900000	673050000	1668966667	1020816667	0.088021404	0.611645929	-0.709231352	
P62072	TIMM10		46375000	57105000	0	70209000	42744000	0	34493333.33	37651000	0.912254489	1.09154426	0.12637063	
Q9Y5J6	TIMM10B		192560000	0	138320000	0	0	0	110293333.3	0	0.126693793	0	N/A	
Q9Y5L4	TIMM13		989060000	0	1126600000	441460000	412340000	439790000	705220000	431196666.7	0.483205258	0.611435675	-0.709727365	
Q99595	TIMM17A		42813000	0	177010000	51239000	125480000	0	73274333.33	58906333.33	0.834821148	0.803914968	-0.314885183	
O60830	TIMM17B		123340000	0	0	130500000	140990000	240250000	41113333.33	170580000	0.074454443	4.149018972	2.052770254	
Q9BVV7	TIMM21		122620000	110680000	0	113500000	154610000	122410000	77766666.67	130173333.3	0.270149654	1.673896271	0.743210129	
Q9Y584	TIMM22		238210000	307340000	233950000	152610000	259660000	261340000	259833333.3	224536666.7	0.458994285	0.864156511	-0.210635467	
O14925	TIMM23		113980000	0	80075000	0	0	0	64685000	0	0.128114686	0	N/A	
O43615	TIMM44		1567800000	1569500000	1521500000	1036500000	851720000	679320000	1552933333	855846666.7	0.002607872	0.551116167	-0.859571645	
Q3ZCQ8	TIMM50		1052800000	1310000000	1072600000	774190000	650150000	826030000	1145133333	750123333.3	0.015578508	0.655053269	-0.610315864	
O60220	TIMM8A		728590000	674920000	693020000	321450000	363570000	446210000	698843333.3	377076666.7	0.001282939	0.539572532	-0.890111189	
Q9Y5J7	TIMM9		384450000	469510000	619780000	220630000	280580000	249160000	491246666.7	250123333.3	0.027306366	0.509160368	-0.973807969	
Q9NS69	TOMM22		533310000	461330000	590370000	191120000	276410000	561930000	528336666.7	343153333.3	0.192181109	0.64949748	-0.622604168	
Q15785	TOMM34		74216000	91449000	180040000	355860000	489250000	586130000	115235000	477080000	0.008245289	4.140061613	2.049652238	
O96008	TOMM40		1219200000	1025500000	959110000	900690000	638980000	720390000	1067936667	753353333.3	0.045754495	0.705428849	-0.503427518	
Q96B49	TOMM6		3757400000	2276100000	3312000000	1037600000	213830000	2292600000	3115166667	1181343333	0.060732419	0.379223156	-1.398881036	
O94826	TOMM70		3418200000	3339900000	3659900000	2893400000	3569100000	3403300000	3472666667	3288600000	0.459191191	0.946995585	-0.078570396	
Q12931	TRAP1		5529400000	5321800000	5169700000	3499100000	3996400000	5561100000	5340300000	4352200000	0.191832692	0.814972942	-0.295175935	
Q96Q11	TRNT1		118460000	0	119590000	0	0	82242000	79350000	27414000	0.342128892	0.345482042	-1.533317375	
P30536	TSPO		0	0	29531000	29892000	82218000	277100000	9843666.667	129736666.7	0.189135998	13.17970946	3.720246662	
Q99757	TXN2		177180000	0	0	138570000	122170000	117470000	59060000	126070000	0.322391585	2.134608872	1.093971747	
Q9BRT2	UQCC2		0	152500000	176970000	126550000	130130000	0	109823333.3	85560000	0.746254765	0.779069415	-0.360176218	
Q9UDW1	UQCR10		113580000	121780000	0	523730000	553390000	583480000	78453333.33	553533333.3	0.000378726	7.055574439	2.818763547	
P14927	UQCRB		2008000000	1994200000	2763900000	1730000000	1803500000	1496800000	2255366667	1676766667	0.099271229	0.743456349	-0.427680057	
P31930	UQCRC1		8950100000	9843900000	11414000000	7925300000	8430100000	8246200000	10069333333	8200533333	0.063827335	0.81440678	-0.296178523	
P22695	UQCRC2		7888900000	7270500000	8851800000	9476400000	6727300000	9557500000	8003733333	8587066667	0.604041053	1.072882655	0.101492292	
P47985	UQCRFS1		4146200000	3054600000	2944000000	2110200000	2160300000	2877500000	3381600000	2382666667	0.093988526	0.704597429	-0.505128884	

P07919	UQCRH		926520000	1128600000	749430000	812370000	1107200000	1203100000	934850000	1040890000	0.545366973	1.113429962	0.155010811	
O14949	UQCRQ		606230000	0	564550000	701760000	812360000	623510000	390260000	712543333.3	0.187622705	1.825816977	0.868542154	
P21796	VDAC1		630650000	731850000	896710000	8791700000	10817000000	8131800000	753070000	9246833333	0.000471072	12.27884969	3.618103507	
P45880	VDAC2		3636900000	3500300000	3084400000	10293000000	10448000000	8295500000	3407200000	9678833333	0.000920183	2.840700086	1.506246523	
Q9Y277	VDAC3		1869000000	1741900000	1917800000	2317500000	2292600000	1475700000	1842900000	2028600000	0.545465037	1.100765098	0.138506633	
Q9NRG9	AAAS		114770000	76845000	133410000	98505000	105080000	63526000	108341666.7	89037000	0.411068854	0.821816783	-0.283111301	
P49588	AARS1		84513000	13493000	0	91121000	85120000	0	32668666.67	58747000	0.544300286	1.798267453	0.846607606	
P08183	ABCB1		61641000	61136000	68210000	0	61112000	118650000	63662333.33	59920666.67	0.918463438	0.941226366	-0.08738636	
P33527	ABCC1		326490000	143880000	176050000	141160000	152970000	0	215473333.3	98043333.33	0.191112196	0.455013768	-1.136017895	
P28288	ABCD3		208830000	208230000	311800000	75476000	116010000	113530000	242953333.3	101672000	0.018535967	0.418483659	-1.256756804	
P61221	ABCE1		0	0	0	188320000	134350000	70689000	0	131119666.7	0.018194198	N/A	N/A	
Q8NE71	ABCF1		80245000	54679000	53487000	186140000	155170000	191090000	62803666.67	177466666.7	0.001288138	2.825737351	1.498627375	
Q8NFV4	ABHD11		97896000	96465000	110040000	114570000	56915000	153090000	101467000	108191666.7	0.823702666	1.066274421	0.092578784	
Q8N2K0	ABHD12		0	0	0	0	68185000	55772000	0	41319000	0.120089929	N/A	N/A	
Q8IZP0	ABI1		153440000	190700000	271100000	172250000	121010000	159870000	205080000	151043333.3	0.228035573	0.73650933	-0.441224294	
O14639	ABLIM1		149490000	136930000	298350000	129200000	82065000	61293000	194923333.3	90852666.67	0.134537734	0.466094362	-1.101306033	
P09110	ACAA1		248060000	371410000	164210000	541870000	498640000	466170000	261226666.7	502226666.7	0.019723841	1.922570437	0.943036455	
Q6JQN1	ACAD10		48028000	45393000	34264000	0	0	37831000	42561666.67	12610333.33	0.087405092	0.296283823	-1.754948235	
P53396	ACLY		487840000	286040000	363620000	150450000	149820000	150920000	379166666.7	150396666.7	0.017653031	0.396650549	-1.334059547	
Q86TX2	ACOT1		1457200000	1418900000	1367500000	1034700000	1004400000	1124800000	1414533333	1054633333	0.001273026	0.745569799	-0.423584673	
O14734	ACOT8		0	157370000	0	25925000	41157000	141660000	52456666.67	69580666.67	0.801651255	1.326440872	0.407560367	
Q15067	ACOX1		412590000	240220000	255130000	0	123550000	113730000	302646666.7	79093333.33	0.030167731	0.261338855	-1.936006459	
P33121	ACSL1		336140000	142870000	153120000	237090000	234450000	235130000	210710000	235556666.7	0.712515793	1.117918783	0.160815379	
O95573	ACSL3		1228900000	1672800000	1352200000	1331200000	1758000000	1099900000	1417966667	1396366667	0.930828183	0.98476692	-0.022145795	
O60488	ACSL4		163190000	125990000	188740000	120370000	46249000	0	159306666.7	55539666.67	0.058401605	0.348633663	-1.520216218	
P68133	ACTA1		1961500000	1803800000	2847500000	2534500000	4074500000	6439700000	2204266667	4349566667	0.143516705	1.973248851	0.980572909	
P63261	ACTG1		60745000000	59727000000	61586000000	55073000000	56735000000	57747000000	60686000000	56518333333	0.011674917	0.931324084	-0.102644809	
O96019	ACTL6A		62657000	4571500	0	0	0	0	22409500	0	0.32877159	0	N/A	
P12814	ACTN1		1398600000	1313600000	1306000000	1133500000	1151800000	947860000	1339400000	1077720000	0.021668351	0.804628938	-0.31360447	
O43707	ACTN4		359500000	175350000	274360000	171960000	115110000	0	269736666.7	95690000	0.076760193	0.354753401	-1.495111578	
P61163	ACTR1A		270510000	220870000	211840000	151340000	233900000	207090000	234406666.7	197443333.3	0.290820884	0.842311083	-0.247574945	
P61160	ACTR2		183820000	212480000	188580000	197640000	183780000	84266000	194960000	155228666.7	0.340941724	0.796207769	-0.328783146	
P61158	ACTR3		1508100000	1308400000	1408900000	1179500000	1201200000	1305700000	1408466667	1228800000	0.061181848	0.872438112	-0.1968753	
O14672	ADAM10		124710000	139840000	161620000	229760000	170570000	102610000	142056666.7	167646666.7	0.540258394	1.180139381	0.23895726	
P78536	ADAM17		155280000	140600000	178780000	221460000	186140000	138190000	158220000	181930000	0.422610309	1.149854633	0.201451484	
P35611	ADD1		117810000	161080000	203290000	105960000	87884000	96451000	160726666.7	96765000	0.064262116	0.602046953	-0.732052088	
Q9UEY8	ADD3		139940000	174850000	157110000	0	0	0	157300000	0	9.83916E-05	0	N/A	
Q9Y653	ADGRG1		64646000	63026000	82617000	0	0	0	70096333.33	0	0.000366202	0	N/A	
Q9NX46	ADPRS		0	0	25691000	30491000	0	0	8563666.667	10163666.67	0.909981305	1.186835857	0.24712042	
P30566	ADSL		125140000	75570000	82114000	146850000	186470000	147040000	94274666.67	160120000	0.031945933	1.698441434	0.764211471	
P55196	AFDN		281010000	264180000	216440000	0	144810000	0	253876666.7	48270000	0.016761776	0.190131691	-2.394929074	
Q9Y4W6	AFG3L2		1070900000	927940000	819540000	777900000	831130000	1023800000	939460000	877610000	0.585064006	0.934164307	-0.098251772	
Q99943	AGPAT1		0	50157000	55878000	0	35754000	0	35345000	11918000	0.334726328	0.33719055	-1.568363989	
O00116	AGPS		2298500000	2183600000	2493300000	2764100000	3578400000	3344100000	2325133333	3228866667	0.024940402	1.388680219	0.473714418	
O00468	AGRN		164770000	69243000	98543000	634000000	383410000	359870000	110852000	459093333.3	0.019455034	4.141497973	2.050152683	
Q6RW13	AGTRAP		0	0	198150000	155190000	229420000	299260000	66050000	227956666.7	0.106718409	3.451274287	1.787129135	
P23526	AHCY		0	0	0	0	137560000	187520000	0	108360000	0.125425537	N/A	N/A	
Q09666	AHNAK		36344000000	34276000000	36873000000	42093000000	43447000000	41361000000	35831000000	42300333333	0.00294764	1.180551292	0.239460724	
Q8IVF2	AHNAK2		237810000	177580000	231380000	251070000	340570000	206110000	215590000	265916666.7	0.315456151	1.233436925	0.302683942	
Q96BJ3	AIDA		0	29413000	0	0	0	44946000	9804333.333	14982000	0.786815241	1.52809982	0.611738787	
Q12904	AIMP1		477800000	480900000	370590000	377150000	463930000	389090000	443096666.7	410056666.7	0.506241381	0.925433878	-0.111798181	
Q13155	AIMP2		141590000	294590000	184540000	192940000	256130000	172930000	206906666.7	207333333.3	0.993846766	1.002062121	0.002971949	
Q02952	AKAP12		0	0	40796000	683350000	290600000	112530000	13598666.67	362160000	0.108399061	26.63202275	4.735090103	
Q9Y2D5	AKAP2		69367000	75767000	46319000	224130000	158270000	59082000	63817666.67	147160666.7	0.162812909	2.305954986	1.205364351	
Q13740	ALCAM		157950000	0	224430000	119100000	145200000	152160000	127460000	138820000	0.874172975	1.089126	0.123170868	
Q8IZ83	ALDH16A1		154540000	125350000	160160000	101750000	105180000	173810000	146683333.3	126913333.3	0.486713379	0.865219861	-0.208861312	
P54886	ALDH18A1		6735600000	6554600000	5844500000	7914000000	8737000000	6674200000	6378233333	7775066667	0.101130584	1.219000037	0.285698169	

P49419	ALDH7A1		2293000000	1743500000	1976400000	1608500000	2076300000	1790300000	2004300000	1825033333	0.440423397	0.910558965	-0.13517565	
P49189	ALDH9A1		329200000	312170000	241230000	342360000	344450000	294020000	294200000	326943333.3	0.358306143	1.11129617	0.152243359	
P04075	ALDOA		11132000000	11189000000	9771400000	8710900000	8583700000	6652400000	10697466667	7982333333	0.028657518	0.746189129	-0.422386753	
P09972	ALDOC		0	0	0	70976000	76114000	79093000	0	75394333.33	5.82646E-06	N/A	N/A	
Q86V81	ALYREF		263930000	235170000	140050000	159300000	168420000	220670000	213050000	182796666.7	0.511481304	0.857998905	-0.220952289	
Q9UKV5	AMFR		0	56197000	0	68593000	0	0	18732333.33	22864333.33	0.895580119	1.22058117	0.287568239	
Q86SJ2	AMIGO2		38662000	0	117480000	103380000	306050000	364520000	52047333.33	257983333.3	0.075572983	4.956706075	2.309381712	
Q01433	AMPD2		0	0	0	28078000	0	44567000	0	24215000	0.136191764	N/A	N/A	
Q9NQW6	ANLN		104700000	79646000	0	0	0	0	61448666.67	0	0.123411276	0	N/A	
P39687	ANP32A		65311000	54388000	49291000	79306000	63219000	55635000	56330000	66053333.33	0.312867154	1.172613764	0.229727896	
P04083	ANXA1		246630000	425500000	493600000	860250000	848960000	1323200000	388576666.7	1010803333	0.02270725	2.601297041	1.37923115	
P07355	ANXA2		44032000000	40393000000	41499000000	54556000000	59182000000	68571000000	41974666667	60769666667	0.011594908	1.447770084	0.53383251	
P08758	ANXA5		203200000	167250000	191720000	352680000	738400000	605180000	187390000	565420000	0.029170762	3.017343508	1.593278949	
P08133	ANXA6		80342000	74680000	82139000	105420000	137080000	127420000	79053666.67	123306666.7	0.0100802	1.559784282	0.641346518	
P20073	ANXA7		0	147160000	0	155280000	193550000	162250000	49053333.33	170360000	0.073976783	3.472954607	1.796163554	
O43747	AP1G1		0	0	0	17731000	65733000	0	0	27821333.33	0.229457434	N/A	N/A	
Q9BXS5	AP1M1		0	0	0	59813000	0	100290000	0	53367666.67	0.140892071	N/A	N/A	
O95782	AP2A1		1500700000	1962700000	1305500000	1242000000	1681200000	1733400000	1589633333	1552200000	0.888002013	0.976451593	-0.03437957	
P63010	AP2B1		501280000	526860000	619100000	784920000	716430000	572170000	549080000	691173333.3	0.120405284	1.258784391	0.332031194	
Q96CW1	AP2M1		216430000	175450000	268430000	186370000	281540000	172600000	220103333.3	213503333.3	0.886885793	0.970014084	-0.0439224	
O14617	AP3D1		0	0	0	32186000	25920000	0	0	19368666.67	0.120721442	N/A	N/A	
O43299	AP5Z1		0	0	0	35262000	29594000	0	0	21618666.67	0.119145596	N/A	N/A	
Q9BZZ5	API5		105780000	79681000	0	76229000	82239000	0	61820333.33	52822666.67	0.838528195	0.854454575	-0.226924298	
Q06481	APLP2		33293000	24524000	52715000	106230000	89643000	47246000	36844000	81039666.67	0.085373378	2.199534976	1.137198543	
Q9HDC9	APMAP		1028800000	1200100000	1334000000	476100000	788010000	825360000	1187633333	696490000	0.025637611	0.586452047	-0.769914947	
Q9BQE5	APOL2		64418000	115310000	118500000	107740000	153780000	88194000	99409333.33	116571333.3	0.547774274	1.172639725	0.229759837	
P05067	APP		112150000	76825000	70931000	100420000	149080000	150400000	86635333.33	133300000	0.089147531	1.538633198	0.621649342	
P48444	ARCN1		53069000	0	0	50848000	0	70260000	17689666.67	40369333.33	0.45462542	2.282085587	1.190352899	
P84077	ARF1		0	0	0	40219000	0	76793000	0	39004000	0.153432533	N/A	N/A	
P18085	ARF4		0	0	0	0	25194000	66714000	0	30636000	0.190349565	N/A	N/A	
P62330	ARF6		43398000	136340000	122530000	109910000	185070000	135160000	100756000	143380000	0.306769653	1.423041804	0.508978044	
Q8N6T3	ARFGAP1		208540000	198410000	264260000	443710000	554510000	630610000	223736666.7	542943333.3	0.005314267	2.426706992	1.278999924	
P53365	ARFIP2		0	0	0	74475000	0	66451000	0	46975333.33	0.117404407	N/A	N/A	
Q07960	ARHGAP1		0	57269000	77505000	48798000	58569000	220840000	44924666.67	109402333.3	0.346065433	2.435239735	1.284063804	
P52565	ARHGDIA		0	0	164110000	326280000	580140000	553890000	54703333.33	486770000	0.011373942	8.898360856	3.153539605	
Q9NXU5	ARL15		102890000	123140000	288190000	0	0	0	171406666.7	0	0.04320614	0	N/A	
Q15041	ARL6IP1		149980000	153590000	214290000	207510000	108560000	0	172620000	105356666.7	0.34887869	0.610338702	-0.71231802	
O75915	ARL6IP5		250780000	295210000	278650000	261920000	368710000	523900000	274880000	384843333.3	0.227211851	1.40004123	0.485469314	
Q96BM9	ARL8A		0	0	0	0	60125000	67539000	0	42554666.67	0.117456274	N/A	N/A	
Q9NVJ2	ARL8B		183930000	230360000	223360000	755590000	780010000	651660000	212550000	729086666.7	0.000249227	3.430188975	1.778288059	
Q8N2F6	ARMC10		161520000	0	0	0	0	109160000	53840000	36386666.67	0.801532507	0.675829619	-0.565268516	
Q9UH62	ARMCX3		0	0	0	40080000	0	37575000	0	25885000	0.116530251	N/A	N/A	
Q92747	ARPC1A		0	0	106880000	0	97833000	0	35626666.67	32611000	0.953209334	0.915353668	-0.127598826	
O15143	ARPC1B		161490000	209070000	172370000	227750000	210850000	161890000	180976666.7	200163333.3	0.476268868	1.10601735	0.145374018	
O15144	ARPC2		100140000	137080000	142480000	223050000	174340000	89850000	126566666.7	162413333.3	0.432571815	1.283223598	0.359772577	
P59998	ARPC4		702390000	861960000	799580000	369650000	527940000	695370000	787976666.7	530986666.7	0.070413725	0.673860901	-0.569477275	
Q9BPX5	ARPC5L		64694000	196020000	112020000	108120000	122910000	0	124244666.7	77010000	0.43537469	0.619825398	-0.690066222	
O00192	ARVCF		291980000	332070000	443550000	210610000	204210000	202440000	355866666.7	205753333.3	0.029775686	0.578175347	-0.790421001	
Q12797	ASPH		1247800000	1174000000	1224400000	518900000	707750000	910720000	1215400000	712456666.7	0.012010606	0.586191103	-0.770557023	
Q8NBU5	ATAD1		205650000	275930000	226200000	292980000	217200000	219190000	235926666.7	243123333.3	0.835656016	1.030503829	0.043349865	
Q9NVI7	ATAD3A		412710000	479820000	625140000	492140000	475380000	708290000	505890000	558603333.3	0.618323935	1.1041992	0.143000461	
Q5T9A4	ATAD3B		800600000	1103200000	838300000	1233300000	1005100000	867030000	914033333.3	1035143333	0.444946792	1.132500638	0.179511863	
P31939	ATIC		597850000	373970000	418140000	676290000	721940000	864120000	463320000	754116666.7	0.030655525	1.627636767	0.702778775	
Q8NHH9	ATL2		277000000	176080000	168510000	123120000	46671000	143760000	207196666.7	104517000	0.088292684	0.504433791	-0.987263173	
Q6DD88	ATL3		115180000	142010000	132970000	0	81937000	127310000	130053333.3	69749000	0.188429056	0.536310744	-0.898858939	
P05023	ATP1A1		7437900000	7194300000	8869300000	8236800000	7418500000	5223700000	7833833333	6959666667	0.448025952	0.888411378	-0.170700225	
P05026	ATP1B1		603250000	637250000	887890000	458030000	743930000	988420000	709463333.3	730126666.7	0.912990035	1.029125301	0.041418648	

P54709	ATP1B3		125620000	206160000	296010000	483640000	388420000	400450000	209263333.3	424170000	0.020281606	2.026967617	1.01932304	
P16615	ATP2A2		1668400000	1597600000	1723300000	382660000	468200000	494770000	1663100000	448543333.3	1.66051E-05	0.269703165	-1.890555644	
P20020	ATP2B1		0	86537000	66203000	104960000	99581000	127200000	50913333.33	110580333.3	0.095489224	2.171932696	1.118979398	
P23634	ATP2B4		312830000	389830000	292590000	227620000	203100000	241510000	331750000	224076666.7	0.027304615	0.675438332	-0.566104038	
Q93050	ATP6V0A1		179120000	180340000	135550000	205410000	266490000	382140000	165003333.3	284680000	0.090473402	1.725298479	0.786845972	
P61421	ATP6V0D1		340770000	343600000	382120000	420240000	672250000	688730000	355496666.7	593740000	0.053512294	1.670170372	0.739995278	
P38606	ATP6V1A		579460000	670580000	773530000	903960000	837850000	1100900000	674523333.3	947570000	0.047894825	1.404799439	0.490364173	
P21281	ATP6V1B2		660190000	637090000	576360000	780830000	955770000	805800000	624546666.7	847466666.7	0.020644391	1.356930894	0.440347249	
P21283	ATP6V1C1		0	153830000	0	96230000	113950000	211110000	51276666.67	140430000	0.226800658	2.738672561	1.453476786	
Q9Y5K8	ATP6V1D		124180000	149100000	0	135690000	93485000	0	91093333.33	76391666.67	0.821691071	0.838608753	-0.253930207	
P36543	ATP6V1E1		209400000	177200000	148270000	350700000	221250000	105990000	178290000	225980000	0.548451558	1.267485557	0.341969308	
O75348	ATP6V1G1		555990000	446810000	474910000	514110000	401410000	332220000	492570000	415913333.3	0.2859219	0.844374065	-0.244045828	
Q9UI12	ATP6V1H		0	0	0	100280000	123320000	85981000	0	103193666.7	0.000688733	N/A	N/A	
Q9Y679	AUP1		360920000	289530000	240200000	163470000	210740000	0	296883333.3	124736666.7	0.077347627	0.42015382	-1.251010493	
P30530	AXL		0	36575000	59550000	0	0	0	32041666.67	0	0.138323882	0	N/A	
O94766	B3GAT3		0	18675000	47285000	80226000	83421000	0	21986666.67	54549000	0.346661017	2.481003639	1.310923851	
Q92934	BAD		41784000	0	30356000	0	0	0	24046666.67	0	0.126001418	0	N/A	
O95816	BAG2		1188500000	959790000	1020700000	782150000	1094600000	805730000	1056330000	894160000	0.252931185	0.8464779	-0.240455693	
Q9UL15	BAG5		0	0	0	37396000	0	56205000	0	31200333.33	0.131924025	N/A	N/A	
Q9UQB8	BAIAP2		486230000	509420000	636510000	277160000	241410000	258740000	544053333.3	259103333.3	0.00398706	0.476246201	-1.070220509	
Q9UHR4	BAIAP2L1		250840000	285570000	322350000	98048000	183080000	0	286253333.3	93709333.33	0.027505726	0.327365038	-1.611027842	
Q16611	BAK1		138360000	122520000	0	0	0	0	86960000	0	0.117580833	0	N/A	
O75531	BANF1		0	0	104080000	0	0	151170000	34693333.33	50390000	0.810164789	1.45244043	0.538478995	
P50895	BCAM		470050000	542200000	817510000	306120000	0	279650000	609920000	195256666.7	0.045222509	0.320134881	-1.643248217	
P51572	BCAP31		471370000	412470000	269760000	458610000	581120000	223450000	384533333.3	421060000	0.777459539	1.094989598	0.130917165	
O75934	BCAS2		267360000	189220000	0	0	22808000	138650000	152193333.3	53819333.33	0.336889319	0.353624776	-1.499708739	
Q9BXK5	BCL2L13		0	0	0	168520000	142660000	194290000	0	168490000	0.000348963	N/A	N/A	
P55957	BID		0	0	184440000	0	216770000	0	61480000	72256666.67	0.915035068	1.175287356	0.233013537	
Q6QNY1	BLOC1S2		247010000	126850000	219460000	191700000	0	228830000	197773333.3	140176666.7	0.509750325	0.708774354	-0.496601691	
O60238	BNIP3L		0	0	0	224270000	135080000	73906000	0	144418666.7	0.029708674	N/A	N/A	
Q8TDN6	BRIX1		0	0	0	56534000	54223000	0	0	36919000	0.116289662	N/A	N/A	
P35613	BSG		0	373860000	273230000	186470000	207930000	226020000	215696666.7	206806666.7	0.940692282	0.958784713	-0.060721188	
Q7L1Q6	BZW1		255040000	38725000	0	167410000	45038000	279850000	97921666.67	164099333.3	0.560500782	1.675822511	0.744869359	
Q9Y6E2	BZW2		0	0	0	58618000	52323000	0	0	36980333.33	0.117395575	N/A	N/A	
Q99622	C12orf57		38447000	72558000	77149000	0	0	0	62718000	0	0.006802596	0	N/A	
Q9UFG5	C19orf25		61095000	0	49852000	0	0	47662000	36982333.33	15887333.33	0.439395014	0.429592508	-1.218959261	
Q6P1X6	C8orf82		1259300000	889050000	596730000	615710000	750000000	1110800000	915026666.7	825503333.3	0.730273227	0.902163143	-0.148539748	
Q9HB71	CACYBP		584310000	569130000	0	513290000	397050000	474970000	384480000	461770000	0.712500051	1.201024761	0.264265894	
P27708	CAD		1298300000	1276600000	1535400000	1397700000	1396800000	1514600000	1370100000	1436366667	0.509689247	1.048366299	0.068142883	
Q9BY67	CADM1		0	0	110510000	74723000	102700000	0	36836666.67	59141000	0.665838735	1.605492716	0.683016119	
Q8N126	CADM3		0	95821000	132330000	0	48382000	109180000	76050333.33	52520666.67	0.665765737	0.69060403	-0.534069342	
Q05682	CALD1		700310000	852520000	1052500000	305320000	350810000	166330000	868443333.3	274153333.3	0.006891598	0.315683618	-1.6634487	
P0DP25	CALM3		9408800000	8111100000	7881100000	5606400000	5717700000	5360100000	8467000000	5561400000	0.00396798	0.656832408	-0.606402783	
P27797	CALR		10851000000	11167000000	12957000000	15350000000	15040000000	14214000000	11658333333	14868000000	0.012174747	1.275310936	0.350849037	
O43852	CALU		1434000000	883550000	1346600000	1519800000	1427600000	1576100000	1221383333	1507833333	0.179318345	1.234529154	0.303960907	
Q86VP6	CAND1		0	0	0	242700000	289260000	0	0	177320000	0.11915471	N/A	N/A	
Q8WVQ1	CANT1		158200000	153820000	138510000	169880000	250360000	204710000	150176666.7	208316666.7	0.073000646	1.38714403	0.472117594	
P27824	CANX		1852300000	1973600000	2374800000	2976400000	3334000000	3507800000	2066900000	3272733333	0.00559674	1.583401874	0.663027464	
Q01518	CAP1		329840000	209060000	72134000	1175400000	1117800000	1039500000	203678000	1110900000	0.000421074	5.454197311	2.447366892	
P17655	CAPN2		28001000	167220000	21667000	181370000	172980000	182640000	72296000	178996666.7	0.088435133	2.475886172	1.307944988	
P04632	CAPNS1		92508000	88663000	99455000	82177000	126890000	142970000	93542000	117345666.7	0.266717393	1.254470363	0.327078387	
Q14444	CAPRIN1		388700000	155620000	319610000	295250000	204380000	266120000	287976666.7	255250000	0.681676624	0.886356534	-0.174040961	
P52907	CAPZA1		872530000	827390000	950760000	0	0	0	883560000	0	1.64235E-05	0	N/A	
P47755	CAPZA2		0	0	141490000	0	226050000	0	47163333.33	75350000	0.767039866	1.597639409	0.675941825	
P47756	CAPZB		792440000	632170000	1120700000	311540000	360400000	667580000	848436666.7	446506666.7	0.091671832	0.526269885	-0.926125255	
Q86X55	CARM1		0	118920000	0	98481000	169220000	148990000	39640000	138897000	0.091438844	3.503960646	1.808986572	
O14936	CASK		0	129050000	125350000	116270000	109800000	96294000	84800000	107454666.7	0.624755167	1.267154088	0.341591969	

Q03135	CAV1		513840000	413410000	561220000	185600000	153210000	165830000	496156666.7	168213333.3	0.001819462	0.339032698	-1.560503674	
Q6NZI2	CAVIN1		2750200000	2036900000	2109500000	529200000	635860000	442120000	2298866667	535726666.7	0.001647093	0.233039469	-2.101353777	
Q13185	CBX3		0	0	45273000	0	29897000	0	15091000	9965666.667	0.79092686	0.660371524	-0.598650184	
Q8N163	CCAR2		64615000	107010000	35646000	135780000	102270000	150490000	69090333.33	129513333.3	0.074226104	1.874550709	0.906544853	
Q96CT7	CCDC124		0	69210000	0	91782000	93953000	0	23070000	61911666.67	0.371354145	2.683643982	1.424193293	
O60826	CCDC22		84315000	0	45875000	75459000	68484000	88739000	43396666.67	77560666.67	0.24487192	1.787249405	0.837740972	
Q96A33	CCDC47		163460000	236180000	73948000	677860000	507170000	600620000	157862666.7	595216666.7	0.003020338	3.77047138	1.914744899	
O00622	CCN1		0	0	26928000	0	41570000	0	8976000	13856666.67	0.782230349	1.543746286	0.626435666	
P78371	CCT2		13528000000	12360000000	15091000000	6434500000	8258500000	6884300000	13659666667	7192433333	0.002556887	0.526545304	-0.92537043	
P49368	CCT3		10931000000	10844000000	11538000000	6966200000	6515300000	5407100000	11104333333	6296200000	0.000716825	0.567003872	-0.818569507	
P50991	CCT4		9957500000	8574200000	8716900000	5256500000	5456500000	5438400000	9082866667	5383800000	0.001132999	0.592742379	-0.754522885	
P48643	CCT5		8639800000	10344000000	7542400000	4398200000	4585100000	4355100000	8842066667	4446133333	0.005794395	0.502838703	-0.991832398	
P40227	CCT6A		10429000000	9973700000	8549500000	4555700000	4602700000	5459500000	9650733333	4872633333	0.001697723	0.504897728	-0.98593691	
Q99832	CCT7		8728500000	8787200000	9474900000	5528200000	4770600000	4688600000	8996866667	4995800000	0.000368631	0.555282209	-0.848706921	
P50990	CCT8		11255000000	11516000000	11369000000	7941300000	8038400000	6375000000	11380000000	7451566667	0.001954051	0.654794962	-0.610884873	
P86791	CCZ1		0	0	0	291740000	341700000	486090000	0	373176666.7	0.003053341	N/A	N/A	
Q6YHK3	CD109		253320000	86990000	170500000	190380000	209760000	474060000	170270000	291400000	0.306161023	1.711399542	0.77517661	
Q9Y5K6	CD2AP		0	0	103490000	204650000	173620000	149790000	34496666.67	176020000	0.020358098	5.102521983	2.351210493	
P25942	CD40		203050000	138150000	185360000	0	197370000	198920000	175520000	132096666.7	0.562379573	0.752601793	-0.410041369	
P16070	CD44		919510000	1003400000	846140000	2155700000	1909800000	1339600000	923016666.7	1801700000	0.023318503	1.951969087	0.964930205	
P13987	CD59		211350000	391900000	574510000	1332200000	825350000	361100000	392586666.7	839550000	0.209734286	2.138508694	1.096605073	
P21926	CD9		379800000	695540000	372900000	219850000	434340000	476590000	482746666.7	376926666.7	0.470229392	0.780796001	-0.356982432	
Q16543	CDC37		118510000	90227000	25673000	117670000	34527000	102470000	78136666.67	84889000	0.865945078	1.086416962	0.119577909	
P60953	CDC42		644460000	877150000	694880000	197950000	410640000	320820000	738830000	309803333.3	0.010224146	0.419316126	-1.253889781	
P55290	CDH13		97299000	109340000	94174000	231850000	171570000	207730000	100271000	203716666.7	0.004651974	2.031660866	1.022659601	
P19022	CDH2		1413800000	1249100000	1370300000	753340000	800660000	824680000	1344400000	792893333.3	0.000501318	0.589774869	-0.761763747	
Q96JB5	CDK5RAP3		99626000	116600000	110000000	173770000	190720000	131920000	108742000	165470000	0.03539073	1.521675158	0.60566041	
Q5VV42	CDKAL1		76083000	23027000	85689000	35347000	0	0	61599666.67	11782333.33	0.093917902	0.19127268	-2.38629727	
Q9UKY7	CDV3		0	0	37758000	91396000	41462000	43788000	12586000	58882000	0.087587531	4.678372795	2.226006827	
P23528	CFL1		4199600000	3579200000	2971000000	3956000000	2681900000	3145900000	3583266667	3261266667	0.565131008	0.910137863	-0.135843001	
Q9Y6H1	CHCHD2		426420000	428030000	622300000	1597300000	1357700000	892240000	492250000	1282413333	0.021926034	2.605207381	1.381398219	
Q9BWS9	CHID1		0	0	0	68746000	46328000	0	0	38358000	0.130988369	N/A	N/A	
Q9UHD1	CHORDC1		179030000	162430000	144830000	231520000	249860000	333450000	162096666.7	271610000	0.029106762	1.675605092	0.744682173	
Q99653	CHP1		213340000	203280000	172210000	309480000	305200000	309790000	196276666.7	308156666.7	0.000853254	1.570011718	0.650775327	
Q14011	CIRBP		109070000	51742000	0	0	0	0	53604000	0	0.164020527	0	N/A	
Q9NZ45	CISD1		486430000	489980000	555330000	230240000	475990000	388890000	510580000	365040000	0.125571864	0.714951624	-0.484082468	
Q8N5K1	CISD2		489450000	414100000	443260000	440560000	0	403990000	448936666.7	281516666.7	0.306233098	0.627074346	-0.673291596	
O14578	CIT		86598000	41257000	0	0	0	0	42618333.33	0	0.163549804	0	N/A	
Q07065	CKAP4		14254000000	13203000000	14246000000	13147000000	12289000000	12124000000	13901000000	12520000000	0.042871387	0.900654629	-0.150954108	
Q14008	CKAP5		500230000	466430000	528720000	300990000	395320000	422420000	498460000	372910000	0.037492502	0.748124223	-0.418650252	
Q7Z460	CLASP1		0	37939000	0	44726000	88263000	0	12646333.33	44329666.67	0.327772006	3.505337515	1.809553362	
P51797	CLCN6		0	0	0	42452000	48783000	29901000	0	40378666.67	0.001894321	N/A	N/A	
P51798	CLCN7		0	0	0	127960000	133240000	136690000	0	132630000	8.03246E-07	N/A	N/A	
Q9Y240	CLEC11A		0	76789000	0	123020000	120670000	0	25596333.33	81230000	0.311030911	3.173501413	1.666075485	
O00299	CLIC1		218100000	294340000	265350000	615890000	722600000	698930000	259263333.3	679140000	0.000432599	2.619498836	1.389290821	
Q9Y696	CLIC4		174320000	0	112270000	103340000	112520000	139050000	95530000	118303333.3	0.684733284	1.238389337	0.308464954	
Q9H078	CLPB		586530000	610180000	649000000	329290000	394330000	328860000	615236666.7	350826666.7	0.000737479	0.570230426	-0.810383075	
O96005	CLPTM1		105550000	75658000	112130000	0	0	0	97779333.33	0	0.000955708	0	N/A	
P09496	CLTA		179330000	224350000	204910000	199620000	233720000	148910000	202863333.3	194083333.3	0.768524202	0.956719631	-0.063831894	
Q00610	CLTC		5097400000	5528800000	5337200000	6828400000	7109000000	6314500000	5321133333	6750633333	0.005635356	1.268645777	0.343289306	
P10909	CLU		1127000000	1141100000	1014800000	2731800000	2433100000	2416500000	1094300000	2527133333	0.000200208	2.309360626	1.20749348	
P62633	CNBP		49557000	0	84429000	152150000	115600000	46088000	44662000	104612666.7	0.204548016	2.342319347	1.227937783	
Q99439	CNN2		321810000	263080000	248900000	209280000	195650000	155280000	277930000	186736666.7	0.02977135	0.671883808	-0.573716333	
Q15417	CNN3		0	142260000	0	101700000	193120000	273440000	47420000	189420000	0.107340678	3.994517081	1.998021099	
Q6P4Q7	CNNM4		0	0	0	54653000	0	65450000	0	40034333.33	0.119321132	N/A	N/A	
P09543	CNP		691450000	665900000	762310000	506440000	523170000	443470000	706553333.3	491026666.7	0.004626027	0.694960513	-0.524997088	
Q9Y2B0	CNPY2		833670000	617790000	714050000	1041800000	1207500000	1349300000	721836666.7	1199533333	0.011703942	1.661779442	0.732728915	

Q9BT09	CNPY3		258790000	335210000	380280000	273090000	300170000	252240000	324760000	275166666.7	0.262684258	0.847292359	-0.239068235	
Q96JB2	COG3		0	0	0	0	90361000	264880000	0	118413666.7	0.202377261	N/A	N/A	
P08572	COL4A2		0	0	0	264840000	209270000	0	0	158036666.7	0.121552159	N/A	N/A	
P12109	COL6A1		286710000	353940000	292410000	1787000000	1752400000	1911900000	311020000	1817100000	9.13104E-06	5.842389557	2.546558557	
P12110	COL6A2		106240000	111650000	0	0	198840000	219740000	72630000	139526666.7	0.444244485	1.921061086	0.941903395	
Q8NBJ5	COLGALT1		859620000	995360000	845020000	516860000	621550000	836360000	900000000	658256666.7	0.083769347	0.731396296	-0.451274774	
Q9Y6G5	COMMD10		107270000	70201000	87346000	62418000	0	124390000	88272333.33	62269333.33	0.525915471	0.70542299	-0.5034395	
Q9H0A8	COMMD4		41628000	0	0	0	37286000	0	13876000	12428666.67	0.941801744	0.895695205	-0.158920212	
Q9P000	COMMD9		145020000	229090000	63331000	22604000	259670000	135060000	145813666.7	139111333.3	0.939901724	0.954034944	-0.067885985	
P21964	COMT		977710000	880010000	892150000	599230000	622580000	788120000	916623333.3	669976666.7	0.021111863	0.730918189	-0.452218159	
Q86VU5	COMTD1		371860000	226400000	177700000	41819000	102630000	0	258653333.3	48149666.67	0.03246091	0.186155214	-2.425422068	
P53621	COPA		250140000	226280000	318910000	424480000	419220000	357850000	265110000	400516666.7	0.018096387	1.510756541	0.595271189	
P53618	COPB1		98067000	0	100030000	87007000	100560000	0	66032333.33	62522333.33	0.942390555	0.946844223	-0.078801006	
O14579	COPE		0	0	80519000	0	0	96772000	26839666.67	32257333.33	0.903505701	1.201852979	0.265260424	
Q9Y678	COPG1		259380000	232630000	185000000	169210000	237760000	225320000	225670000	210763333.3	0.648451975	0.933944846	-0.098590741	
P61923	COPZ1		178200000	0	0	88914000	140290000	170770000	59400000	133324666.7	0.312511002	2.244523008	1.166408885	
Q9BR76	CORO1B		352340000	372300000	468970000	183820000	159070000	327820000	397870000	223570000	0.052239037	0.561917209	-0.83157051	
Q9ULV4	CORO1C		1280200000	1574800000	1470300000	815410000	710360000	812200000	1441766667	779323333.3	0.002043079	0.540533605	-0.887543783	
Q9UI42	CPA4		54888000	44285000	0	300390000	324350000	325380000	33057666.67	316706666.7	0.000109839	9.580430157	3.260090434	
O75976	CPD		284890000	218860000	246680000	197850000	188670000	313910000	250143333.3	233476666.7	0.727698222	0.933371534	-0.099476627	
O75131	CPNE3		34823000	0	0	0	48826000	0	11607666.67	16275333.33	0.826841237	1.402119289	0.487609096	
Q16630	CPSF6		0	0	102040000	93419000	161600000	0	34013333.33	85006333.33	0.428111746	2.499206194	1.321469934	
Q6UXH1	CRELD2		63730000	111350000	86239000	120270000	92771000	0	87106333.33	71013666.67	0.700289873	0.815252622	-0.294680918	
O75718	CRTAP		323110000	337390000	253160000	330600000	378620000	332120000	304553333.3	347113333.3	0.234417391	1.139745638	0.188711888	
Q08257	CRYZ		643630000	471010000	489170000	487930000	460930000	630700000	534603333.3	526520000	0.92039921	0.984879755	-0.0219805	
O75534	CSDE1		118400000	161640000	188980000	414910000	92536000	224580000	156340000	244008666.7	0.411887277	1.560756471	0.642245447	
P55060	CSE1L		377750000	385560000	538510000	719050000	782990000	1195800000	433940000	899280000	0.042409725	2.072360234	1.051274806	
P67870	CSNK2B		0	0	0	80187000	0	46227000	0	42138000	0.143991357	N/A	N/A	
Q6UVK1	CSPG4		2205600000	2339800000	2611400000	2386600000	2525700000	2146800000	2385600000	2353033333	0.851176184	0.986348647	-0.019830405	
P21291	CSRP1		250510000	226290000	368820000	342150000	310730000	270630000	281873333.3	307836666.7	0.621867283	1.092109931	0.127118084	
Q16527	CSRP2		0	108970000	124790000	0	0	62731000	77920000	20910333.33	0.268944689	0.268356434	-1.897777617	
Q8NHU0	CT45A3		52593000	0	55481000	0	0	77834000	36024666.67	25944666.67	0.765665969	0.72019172	-0.473547081	
P35221	CTNNA1		3765900000	4255800000	4028100000	2260400000	2562700000	2573000000	4016600000	2465366667	0.00089009	0.61379442	-0.704172565	
P35222	CTNNB1		2526900000	2555800000	2098000000	1647700000	1490400000	1549500000	2393566667	1562533333	0.005835594	0.652805437	-0.615275022	
O60716	CTNND1		1880900000	1697800000	2106800000	1492100000	1468100000	1300500000	1895166667	1420233333	0.023217222	0.74939759	-0.416196756	
P17812	CTPS1		0	0	0	57427000	45836000	96634000	0	66632333.33	0.012297665	N/A	N/A	
P10619	CTSA		209020000	143960000	156620000	324490000	308070000	457230000	169866666.7	363263333.3	0.019542947	2.138520408	1.096612975	
P07858	CTSB		1316000000	1020100000	1171300000	2628200000	2634300000	3475700000	1169133333	2912733333	0.004059842	2.491361122	1.316934155	
P53634	CTSC		581840000	534550000	522080000	342220000	413740000	385220000	546156666.7	380393333.3	0.003883501	0.696491239	-0.52182289	
P07339	CTSD		843690000	1152100000	924550000	1213000000	1153100000	992520000	973446666.7	1119540000	0.267065463	1.150078416	0.201732231	
P07711	CTSL		0	0	167670000	251580000	180430000	322710000	55890000	251573333.3	0.04776428	4.50122264	2.170316925	
Q9UBR2	CTSZ		353450000	284510000	350880000	613780000	517330000	258270000	329613333.3	463126666.7	0.285975003	1.405060475	0.490632227	
Q14247	CTTN		70908000	74320000	85563000	99367000	62288000	57974000	76930333.33	73209666.67	0.801683752	0.951635896	-0.071518404	
Q13948	CUX1		98489000	220980000	215960000	63601000	92418000	301780000	178476333.3	152599666.7	0.776121734	0.855013456	-0.225980969	
P78310	CXADR		0	0	45240000	114480000	0	0	15080000	38160000	0.603809024	2.530503979	1.339424743	
Q7L576	CYFIP1		537910000	563480000	746560000	343860000	294170000	405040000	615983333.3	347690000	0.021396346	0.56444709	-0.825089742	
Q96F07	CYFIP2		0	70760000	24400000	36013000	37160000	0	31720000	24391000	0.775963041	0.768947037	-0.379043863	
Q9NUQ9	CYRIB		381780000	416320000	475960000	445980000	563810000	392490000	424686666.7	467426666.7	0.499269895	1.100638902	0.138341227	
Q14118	DAG1		620740000	593670000	563850000	303640000	257830000	0	592753333.3	187156666.7	0.013388832	0.31574123	-1.663185432	
Q8NCG7	DAGLB		0	0	0	43084000	55781000	58226000	0	52363666.67	0.000367286	N/A	N/A	
P14868	DARS1		265660000	222010000	237810000	232730000	276870000	333340000	241826666.7	280980000	0.285537602	1.1619066	0.216494102	
Q16643	DBN1		2033800000	1694500000	2050000000	748510000	890230000	748010000	1926100000	795583333.3	0.00083281	0.413054012	-1.275597649	
Q8N8Z6	DCBLD1		0	69008000	73952000	148920000	106150000	0	47653333.33	85023333.33	0.498727186	1.784205372	0.835281687	
Q96PD2	DCBLD2		74204000	0	111400000	0	0	212910000	61868000	70970000	0.912905551	1.147119674	0.198015909	
Q14203	DCTN1		0	0	90054000	139220000	114830000	71257000	30018000	108435666.7	0.094947358	3.612354809	1.852939603	
Q13561	DCTN2		366240000	250210000	368770000	217510000	297460000	448200000	328406666.7	321056666.7	0.929568206	0.977619212	-0.032655458	
Q7Z4W1	DCXR		648380000	514530000	308820000	155190000	201570000	297280000	490576666.7	218013333.3	0.063882152	0.444402166	-1.170062246	

P39656	DDOST		2285200000	1530600000	1639300000	1080900000	1204100000	1665800000	1818366667	1316933333	0.164652051	0.724239702	-0.465460828	
Q96HY6	DDRGK1		437220000	357710000	321930000	236480000	352000000	351200000	372286666.7	313226666.7	0.313888645	0.84135881	-0.249206905	
Q92499	DDX1		1244200000	872800000	943800000	555700000	704630000	839570000	1020266667	699966666.7	0.084472727	0.686062467	-0.543588152	
Q92841	DDX17		170610000	161960000	199600000	147070000	180700000	166600000	177390000	164790000	0.447832188	0.928970066	-0.106295985	
Q9NVP1	DDX18		0	0	0	50001000	27783000	0	0	25928000	0.147501418	N/A	N/A	
Q9NUU7	DDX19A		0	0	89652000	0	88213000	90093000	29884000	59435333.33	0.521867922	1.988868068	0.991947578	
Q9NR30	DDX21		135580000	272820000	251390000	111290000	139350000	196380000	219930000	149006666.7	0.224685102	0.677518604	-0.561667532	
Q13838	DDX39B		285050000	205840000	226420000	239070000	317330000	322100000	239103333.3	292833333.3	0.208648123	1.224714559	0.292445543	
O00571	DDX3X		903460000	710640000	672490000	625160000	548490000	846810000	762196666.7	673486666.7	0.481730661	0.883612716	-0.178513915	
Q9H0S4	DDX47		97992000	108200000	121740000	55036000	42424000	0	109310666.7	32486666.67	0.012998401	0.29719576	-1.750514561	
Q9Y6V7	DDX49		232770000	168870000	82746000	0	20270000	0	161462000	6756666.667	0.024517156	0.041846792	-4.578739174	
P17844	DDX5		1517800000	1268300000	1332000000	834610000	1066200000	1231100000	1372700000	1043970000	0.07468922	0.760523057	-0.394936108	
Q9BUN8	DERL1		207940000	143230000	301570000	110700000	139630000	392640000	217580000	214323333.3	0.975739297	0.985032325	-0.021757025	
Q9GZP9	DERL2		0	0	126690000	55814000	112910000	0	42230000	56241333.33	0.80579355	1.33178625	0.41336255	
Q9BTZ2	DHRS4		111720000	125700000	75679000	404770000	317300000	146530000	104366333.3	289533333.3	0.074673776	2.774202409	1.472073052	
Q9Y394	DHRS7		373350000	301740000	443360000	477620000	649250000	1038100000	372816666.7	721656666.7	0.110588298	1.935687782	0.952846271	
Q7Z478	DHX29		0	0	0	10960000	8630000	0	0	6530000	0.121712833	N/A	N/A	
Q7L2E3	DHX30		883400000	1015800000	969240000	849550000	747490000	1024300000	956146666.7	873780000	0.410174738	0.913855615	-0.129961851	
Q08211	DHX9		501360000	424650000	471080000	406750000	287430000	252450000	465696666.7	315543333.3	0.044079324	0.677572669	-0.561552413	
O60610	DIAPH1		0	0	53242000	0	37281000	142890000	17747333.33	60057000	0.41275445	3.384001352	1.758730145	
Q12959	DLG1		483820000	502410000	449430000	298900000	304730000	323910000	478553333.3	309180000	0.000604611	0.64607219	-0.630232719	
Q9NW81	DMAC2		0	0	52595000	52835000	0	0	17531666.67	17611666.67	0.997585533	1.004563171	0.00656829	
P31689	DNAJA1		494330000	599490000	477390000	677400000	677510000	492330000	523736666.7	615746666.7	0.273621027	1.17567989	0.233495302	
O60884	DNAJA2		134300000	194690000	139130000	180990000	312050000	217130000	156040000	236723333.3	0.138044534	1.517068273	0.601286013	
P25685	DNAJB1		26327000	75412000	0	188530000	208880000	246460000	33913000	214623333.3	0.002912772	6.328644866	2.661896613	
Q9UBS4	DNAJB11		365830000	456850000	439520000	533710000	671920000	434150000	420733333.3	546593333.3	0.165848418	1.299144351	0.377561741	
Q9NXW2	DNAJB12		164890000	175900000	160200000	0	0	0	166996666.7	0	3.59754E-06	0	N/A	
P25686	DNAJB2		115360000	70766000	84433000	77227000	70716000	84219000	90186333.33	77387333.33	0.404779623	0.85808271	-0.22081138	
Q96KC8	DNAJC1		0	0	0	0	54134000	45029000	0	33054333.33	0.119459081	N/A	N/A	
Q8IXB1	DNAJC10		161560000	311060000	316810000	554920000	588010000	854250000	263143333.3	665726666.7	0.020042434	2.529901321	1.339081114	
Q9NVH1	DNAJC11		689190000	558270000	552990000	320630000	458880000	334820000	600150000	371443333.3	0.021648563	0.618917493	-0.692180997	
O75165	DNAJC13		73946000	0	177780000	0	0	0	83908666.67	0	0.178994196	0	N/A	
Q13217	DNAJC3		165370000	109950000	155660000	155450000	188360000	126020000	143660000	156610000	0.629426952	1.090143394	0.124517915	
O75937	DNAJC8		76497000	0	0	86232000	86441000	0	25499000	57557666.67	0.45130565	2.257251918	1.174567438	
Q8WXX5	DNAJC9		738370000	627430000	547190000	432850000	550720000	558240000	637663333.3	513936666.7	0.14609867	0.805968667	-0.311204342	
O60762	DPM1		0	0	63905000	57785000	41511000	96258000	21301666.67	65184666.67	0.176641256	3.060073547	1.613566327	
Q9C005	DPY30		106020000	42420000	64570000	0	0	0	71003333.33	0	0.018951274	0	N/A	
Q16555	DPYSL2		0	0	0	338200000	346460000	349100000	0	344586666.7	4.94066E-08	N/A	N/A	
Q9Y295	DRG1		0	0	75398000	0	0	128450000	25132666.67	42816666.67	0.739692446	1.703626091	0.76860873	
Q14126	DSG2		460930000	462840000	546170000	298350000	339710000	407310000	489980000	348456666.7	0.028903391	0.711165082	-0.491743606	
Q03001	DST		265580000	151200000	159130000	274250000	286890000	204440000	191970000	255193333.3	0.231979673	1.329339654	0.410709768	
P60981	DSTN		0	0	0	423320000	281380000	152420000	0	285706666.7	0.021732251	N/A	N/A	
P23919	DTYMK		67216000	89419000	0	109510000	79432000	0	52211666.67	62980666.67	0.811612699	1.206256584	0.270536817	
Q14204	DYNC1H1		2033900000	1764700000	1428300000	2902800000	2890600000	4328500000	1742300000	3373966667	0.032609642	1.936501559	0.953452663	
Q13409	DYNC1I2		73431000	94017000	78670000	0	0	0	82039333.33	0	0.000185734	0	N/A	
Q9Y6G9	DYNC1LI1		0	96491000	0	142250000	0	0	32163666.67	47416666.67	0.803233842	1.474230757	0.559962363	
O43237	DYNC1LI2		0	0	0	40756000	78174000	0	0	39643333.33	0.153903374	N/A	N/A	
P63167	DYNLL1		105990000	151360000	252470000	275080000	304280000	0	169940000	193120000	0.837839149	1.136401083	0.184472111	
Q15125	EBP		321580000	412800000	345490000	0	0	164760000	359956666.7	54920000	0.007633518	0.152573921	-2.712419708	
P42892	ECE1		0	0	90362000	111310000	106420000	71962000	30120666.67	96564000	0.110912078	3.205905137	1.680731737	
Q9NTX5	ECHDC1		224640000	331950000	94511000	0	132270000	0	217033666.7	44090000	0.101372223	0.203148206	-2.299395472	
O75521	ECI2		985210000	1025000000	976560000	548570000	654010000	776680000	995590000	659753333.3	0.007653917	0.662675733	-0.593625005	
Q6P2E9	EDC4		113140000	124480000	76502000	56670000	111210000	196840000	104707333.3	121573333.3	0.716663697	1.161077543	0.215464327	
O60869	EDF1		184810000	0	215100000	225620000	238250000	284070000	133303333.3	249313333.3	0.170535967	1.870270811	0.903247184	
O43854	EDIL3		391010000	362100000	413430000	392600000	509890000	435180000	388846666.7	445890000	0.201512136	1.146698784	0.197486473	
Q15075	EEA1		276960000	303290000	193400000	553890000	633830000	228200000	257883333.3	471973333.3	0.170764384	1.830181607	0.871986812	
P68104	EEF1A1		3664500000	3643100000	3891800000	7753900000	7463500000	6728000000	3733133333	7315133333	0.000343177	1.959515688	0.970497124	

Q05639	EEF1A2		40388000	0	72716000	132790000	137560000	239840000	37701333.33	170063333.3	0.031458894	4.510804216	2.173384669	
P24534	EEF1B2		720730000	493260000	417690000	469200000	153800000	627160000	543893333.3	416720000	0.487053751	0.766179643	-0.3842454	
P29692	EEF1D		1360600000	1251400000	1485200000	1754900000	2144300000	1637100000	1365733333	1845433333	0.045731779	1.351239871	0.434283804	
O43324	EEF1E1		374950000	0	310760000	150510000	218690000	357740000	228570000	242313333.3	0.921409184	1.060127459	0.08423773	
P26641	EEF1G		2492200000	2575900000	2479200000	3635500000	3731600000	3469900000	2515766667	3612333333	0.0001827	1.435877731	0.521932905	
P13639	EEF2		3921100000	2866000000	3173700000	3526900000	3608100000	3729800000	3320266667	3621600000	0.398036019	1.090755763	0.125328096	
Q96C19	EFHD2		87050000	80074000	75830000	135550000	90512000	83233000	80984666.67	103098333.3	0.255684629	1.273059921	0.348300326	
Q15029	EFTUD2		0	52807000	0	30418000	52826000	33447000	17602333.33	38897000	0.324011502	2.209763857	1.143892207	
Q8N3D4	EHBP1L1		224410000	78651000	169980000	89272000	104900000	92626000	157680333.3	95599333.33	0.220433466	0.606285713	-0.721930269	
Q9H4M9	EHD1		270540000	264880000	431590000	494660000	576770000	445300000	322336666.7	505576666.7	0.0516521	1.568473956	0.649361574	
Q9NZN4	EHD2		35818000	139110000	0	0	0	0	58309333.33	0	0.234593252	0	N/A	
Q9H223	EHD4		226220000	208830000	200470000	0	0	282280000	211840000	94093333.33	0.280316994	0.444171702	-1.170810613	
P41567	EIF1		0	150240000	51906000	90909000	58750000	58543000	67382000	69400666.67	0.966628297	1.029958545	0.042586271	
Q9BY44	EIF2A		0	87976000	73043000	77555000	0	63314000	53673000	46956333.33	0.861647602	0.874859489	-0.192876771	
P19525	EIF2AK2		156020000	96374000	69343000	84561000	109250000	221890000	107245666.7	138567000	0.560638418	1.292052204	0.369664362	
P05198	EIF2S1		211050000	139310000	155490000	186800000	188250000	135800000	168616666.7	170283333.3	0.954969816	1.009884353	0.014190092	
P20042	EIF2S2		0	0	187420000	142200000	249500000	0	62473333.33	130566666.7	0.515310322	2.089958382	1.063474214	
P41091	EIF2S3		581530000	581490000	565110000	585750000	540710000	485300000	576043333.3	537253333.3	0.259655931	0.932661316	-0.100574815	
Q14152	EIF3A		263670000	281360000	190670000	171270000	175730000	197820000	245233333.3	181606666.7	0.092830134	0.740546418	-0.433337927	
Q99613	EIF3C		288500000	350220000	231440000	169160000	227650000	312280000	290053333.3	236363333.3	0.375358273	0.814896111	-0.295311949	
P60228	EIF3E		52775000	46960000	0	0	0	0	33245000	0	0.117466902	0	N/A	
O00303	EIF3F		200420000	159870000	166170000	0	0	168500000	175486666.7	56166666.67	0.106880436	0.320062303	-1.643575329	
O75821	EIF3G		0	94012000	80742000	94235000	0	73071000	58251333.33	55768666.67	0.954577391	0.957380089	-0.062836293	
Q13347	EIF3I		0	146420000	0	165520000	165140000	102330000	48806666.67	144330000	0.146609896	2.957177981	1.564221076	
O75822	EIF3J		533440000	456940000	724380000	317280000	365260000	816220000	571586666.7	499586666.7	0.706093791	0.87403485	-0.194237289	
Q9Y262	EIF3L		59067000	56019000	91854000	0	0	0	68980000	0	0.003850605	0	N/A	
Q7L2H7	EIF3M		135850000	134210000	43114000	0	0	184710000	104391333.3	61570000	0.567258484	0.58979992	-0.761702469	
P60842	EIF4A1		749660000	507880000	656570000	1062700000	921050000	1074500000	638036666.7	1019416667	0.01135967	1.597739942	0.676032605	
Q14240	EIF4A2		85179000	0	0	0	203080000	0	28393000	67693333.33	0.62076864	2.384155719	1.253478467	
P38919	EIF4A3		0	0	0	0	38704000	38990000	0	25898000	0.116121913	N/A	N/A	
P23588	EIF4B		149810000	41626000	114870000	95418000	77785000	209360000	102102000	127521000	0.65127888	1.248956925	0.320723722	
P06730	EIF4E		168060000	150690000	147430000	271980000	246400000	139380000	155393333.3	219253333.3	0.195336414	1.410957141	0.496674166	
Q04637	EIF4G1		194720000	219210000	267310000	348800000	308150000	403410000	227080000	353453333.3	0.022284195	1.556514591	0.638319101	
Q15056	EIF4H		333110000	252630000	282190000	250440000	156350000	162220000	289310000	189670000	0.060601345	0.655594345	-0.609124684	
P55010	EIF5		82158000	0	27667000	68222000	88473000	89366000	36608333.33	82020333.33	0.144707548	2.240482586	1.163809513	
P63241	EIF5A		998740000	609140000	620750000	783700000	769320000	1076500000	742876666.7	876506666.7	0.456983768	1.179881811	0.238642352	
O60841	EIF5B		0	0	94701000	163670000	100530000	132880000	31567000	132360000	0.050585848	4.192986347	2.067978132	
Q9BQ52	ELAC2		207720000	133440000	129750000	236530000	89835000	118450000	156970000	148271666.7	0.874268946	0.944586014	-0.08224592	
Q15717	ELAVL1		0	0	0	0	81737000	52171000	0	44636000	0.135134262	N/A	N/A	
P0C7U0	ELFN1		87265000	0	0	0	65043000	0	29088333.33	21681000	0.848184664	0.74535037	-0.424009337	
Q8IZ81	ELMOD2		0	0	0	31953000	29887000	0	0	20613333.33	0.116560282	N/A	N/A	
Q8N766	EMC1		38541000	66166000	0	83096000	222480000	185720000	34902333.33	163765333.3	0.048468387	4.692102725	2.230234599	
Q5UCC4	EMC10		104690000	130210000	156050000	119940000	116340000	0	130316666.7	78760000	0.287820656	0.604373961	-0.726486591	
Q15006	EMC2		67227000	111570000	151010000	117020000	136280000	0	109935666.7	84433333.33	0.63007328	0.768024936	-0.380774943	
Q5J8M3	EMC4		173320000	171690000	0	0	0	185020000	115003333.3	61673333.33	0.561439119	0.536274311	-0.898956949	
Q9NPA0	EMC7		358890000	0	277750000	296140000	306920000	269560000	212213333.3	290873333.3	0.511267371	1.37066474	0.454875736	
O43402	EMC8		60862000	76083000	44909000	84360000	92622000	0	60618000	58994000	0.960645334	0.973209278	-0.039178021	
P50402	EMD		189970000	197470000	297720000	246210000	240480000	244420000	228386666.7	243703333.3	0.682370277	1.067064627	0.093647556	
Q8N8S7	ENAH		85003000	136930000	172680000	227000000	165670000	106330000	131537666.7	166333333.3	0.465174259	1.264530059	0.338601331	
P06733	ENO1		1786500000	1891500000	1757200000	3450800000	3754500000	3507300000	1811733333	3570866667	6.57542E-05	1.97096703	0.978903643	
P11171	EPB41		85876000	0	69576000	0	78131000	52239000	51817333.33	43456666.67	0.822685646	0.838651159	-0.253857255	
O43491	EPB41L2		1548800000	1489100000	1669000000	725840000	573490000	460810000	1568966667	586713333.3	0.000459432	0.373948884	-1.419087019	
Q9Y2J2	EPB41L3		2218600000	2032100000	2109800000	2050700000	2042900000	1773000000	2120166667	1955533333	0.195731306	0.922348872	-0.116615552	
Q9HCM4	EPB41L5		117200000	53170000	0	0	0	0	56790000	0	0.169015652	0	N/A	
P16422	EPCAM		0	0	53345000	0	0	54518000	17781666.67	18172666.67	0.98846667	1.02198894	0.031379583	
P29317	EPHA2		192880000	252240000	379850000	117590000	67863000	186460000	274990000	123971000	0.080833095	0.45082003	-1.149376479	
P29323	EPHB2		208080000	391760000	338220000	241640000	291940000	356010000	312686666.7	296530000	0.812552283	0.948329531	-0.076539632	

P54760	EPHB4		111690000	107230000	94145000	0	0	0	104355000	0	3.82216E-05	0	N/A	
Q9Y6I3	EPN1		78452000	75484000	122390000	56399000	64309000	84899000	92108666.67	68535666.67	0.246542204	0.744074029	-0.426481932	
P07814	EPRS1		1290400000	1144500000	1015100000	1064800000	1262100000	1408500000	1150000000	1245133333	0.49683604	1.082724638	0.114666378	
Q9UBC2	EPS15L1		111030000	56987000	161580000	109960000	226970000	159440000	109865666.7	165456666.7	0.288038615	1.505990649	0.590712812	
Q12929	EPS8		65563000	92220000	90168000	0	0	0	82650333.33	0	0.000644839	0	N/A	
Q9H6S3	EPS8L2		0	0	0	70438000	0	101480000	0	57306000	0.128920027	N/A	N/A	
Q96RT1	ERBIN		497250000	216560000	322620000	0	11774000	0	345476666.7	3924666.667	0.01404118	0.01136015	-6.459874338	
Q8IUD2	ERC1		159570000	139930000	195350000	104070000	108100000	0	164950000	70723333.33	0.072695422	0.428756189	-1.221770599	
Q96RQ1	ERGIC2		113620000	131190000	117620000	155860000	114790000	116270000	120810000	128973333.3	0.602607098	1.067571669	0.094332925	
P84090	ERH		178310000	278410000	278810000	407890000	373490000	217370000	245176666.7	332916666.7	0.263405514	1.357864397	0.441339412	
Q96DZ1	ERLEC1		202990000	241060000	199280000	245580000	261910000	265000000	214443333.3	257496666.7	0.04242088	1.20076788	0.263957291	
O75477	ERLIN1		942920000	913520000	869700000	507330000	422490000	576850000	908713333.3	502223333.3	0.001193209	0.552675211	-0.855496189	
O94905	ERLIN2		47985000	0	0	0	35481000	12076000	15995000	15852333.33	0.994394156	0.991080546	-0.012925784	
Q96HE7	ERO1A		528770000	669760000	792220000	652760000	785220000	997850000	663583333.3	811943333.3	0.304548403	1.22357403	0.291101391	
P30040	ERP29		4697900000	4721100000	6592300000	4641300000	5216300000	5707100000	5337100000	5188233333	0.84179251	0.972107199	-0.040812679	
Q9BS26	ERP44		432710000	432960000	172650000	247370000	352500000	767120000	346106666.7	455663333.3	0.577278664	1.31654018	0.396751553	
Q14674	ESPL1		0	17830000	0	5065300	0	0	5943333.333	1688433.333	0.528898796	0.284088615	-1.815587081	
Q9BSJ8	ESYT1		4580100000	4205800000	4393500000	2857900000	2885700000	3071000000	4393133333	2938200000	0.0003321	0.668816486	-0.580317685	
P62495	ETF1		617590000	537940000	470280000	376680000	452140000	531840000	541936666.7	453553333.3	0.22589377	0.836912062	-0.256852054	
P38117	ETFB		3551500000	3424400000	3427400000	2698600000	2893500000	2428100000	3467766667	2673400000	0.004920627	0.770928455	-0.375331115	
Q01844	EWSR1		108830000	146020000	119220000	91101000	0	112600000	124690000	67900333.33	0.19224307	0.544553158	-0.876855205	
Q9NVH0	EXD2		55175000	82999000	52949000	110690000	154290000	134030000	63707666.67	133003333.3	0.012023437	2.087713148	1.061923499	
Q9NV70	EXOC1		0	148680000	222790000	143170000	0	182780000	123823333.3	108650000	0.868320684	0.877459822	-0.188595028	
Q96KP1	EXOC2		0	41836000	37752000	31135000	38944000	0	26529333.33	23359666.67	0.867733283	0.880522189	-0.183568734	
O00471	EXOC5		97304000	378840000	101600000	57353000	129340000	0	192581333.3	62231000	0.263866338	0.323141391	-1.629762537	
Q9Y2D4	EXOC6B		116640000	173550000	128490000	81950000	103950000	122820000	139560000	102906666.7	0.155491674	0.737365052	-0.439549054	
Q9UPT5	EXOC7		344750000	270100000	196640000	201460000	229350000	348540000	270496666.7	259783333.3	0.871505581	0.960393843	-0.05830194	
Q8IYI6	EXOC8		80797000	86832000	0	42139000	0	112940000	55876333.33	51693000	0.927574301	0.925132286	-0.112268421	
P15311	EZR		2006800000	1810800000	1817600000	2554100000	3427000000	2723700000	1878400000	2901600000	0.020421519	1.54471891	0.627344337	
O95864	FADS2		469750000	487980000	416080000	229460000	0	214070000	457936666.7	147843333.3	0.015862753	0.322846682	-1.631078895	
Q96CS3	FAF2		787810000	754500000	707720000	561180000	697160000	672170000	750010000	643503333.3	0.089844413	0.857993005	-0.22096221	
Q96GK7	FAHD2A		178800000	316560000	255400000	174060000	211640000	265880000	250253333.3	217193333.3	0.528372593	0.867893868	-0.204409465	
Q9NZB2	FAM120A		0	0	0	59297000	43356000	0	0	34217666.67	0.1256191	N/A	N/A	
Q96C01	FAM136A		160980000	154090000	243400000	0	101700000	0	186156666.7	33900000	0.026576298	0.182104679	-2.457160105	
Q96A26	FAM162A		136910000	168960000	221530000	509780000	363700000	205680000	175800000	359720000	0.1139506	2.046188851	1.032939303	
O75063	FAM20B		97198000	58705000	18988000	23944000	0	0	58297000	7981333.333	0.103525503	0.136908131	-2.86871996	
Q92520	FAM3C		172780000	32102000	61241000	71332000	59534000	108030000	88707666.67	79632000	0.850945331	0.897690166	-0.155710504	
Q8NCA5	FAM98A		143880000	123290000	81223000	79368000	87952000	86866000	116131000	84728666.67	0.167241459	0.7295956	-0.454831065	
Q52LJ0	FAM98B		39055000	0	37199000	0	0	0	25418000	0	0.116352105	0	N/A	
Q8WVX9	FAR1		0	308110000	239880000	0	0	0	182663333.3	0	0.122246158	0	N/A	
Q9Y285	FARSA		208420000	318370000	0	242820000	178740000	0	175596666.7	140520000	0.781599642	0.800242981	-0.321489977	
Q9NSD9	FARSB		245050000	268050000	268510000	391870000	457410000	548080000	260536666.7	465786666.7	0.011098093	1.787796983	0.838182918	
P25445	FAS		0	0	42953000	0	73550000	65174000	14317666.67	46241333.33	0.30721771	3.229669639	1.6913866	
P49327	FASN		1493600000	1399800000	1405100000	955850000	962050000	950540000	1432833333	956146666.7	9.91839E-05	0.667311853	-0.583566965	
P62861	FAU		0	0	0	247810000	199850000	247540000	0	231733333.3	0.000130249	N/A	N/A	
P35556	FBN2		348780000	293920000	414520000	0	0	0	352406666.7	0	0.000538939	0	N/A	
P37268	FDFT1		0	0	235190000	138400000	146960000	0	78396666.67	95120000	0.864203447	1.213316893	0.278956401	
P39748	FEN1		1194100000	1040200000	1463400000	551230000	522500000	326490000	1232566667	466740000	0.005778056	0.378673229	-1.400974663	
Q96AC1	FERMT2		170490000	257520000	129460000	183200000	125290000	142220000	185823333.3	150236666.7	0.439340464	0.808491937	-0.306694709	
Q9Y613	FHOD1		0	0	0	56592000	66578000	0	0	41056666.67	0.11872448	N/A	N/A	
Q96AY3	FKBP10		242280000	165750000	719410000	989410000	930760000	933190000	375813333.3	951120000	0.029898313	2.530830909	1.339611121	
Q9NWM8	FKBP14		133260000	113180000	96950000	253170000	201740000	170180000	114463333.3	208363333.3	0.02355776	1.820350039	0.864215896	
P26885	FKBP2		1197700000	1331500000	1237500000	817170000	751070000	880100000	1255566667	816113333.3	0.001277262	0.649996018	-0.621497216	
Q00688	FKBP3		520560000	672680000	244570000	1591700000	1509300000	699910000	479270000	1266970000	0.064412826	2.643541219	1.402471822	
Q02790	FKBP4		0	0	0	219320000	303880000	321160000	0	281453333.3	0.000863911	N/A	N/A	
Q14318	FKBP8		151770000	72109000	115410000	180050000	189180000	0	113096333.3	123076666.7	0.886712208	1.088246303	0.122005119	
O95302	FKBP9		179100000	54742000	54194000	407730000	428120000	341450000	96012000	392433333.3	0.003794587	4.087336305	2.031160953	

Q13045	FLII		76950000	62930000	43784000	45577000	21378000	79927000	61221333.33	48960666.67	0.563958924	0.79973212	-0.322411263	
P21333	FLNA		23205000000	24304000000	25038000000	23510000000	22755000000	19664000000	24182333333	21976333333	0.162798673	0.908776379	-0.138002758	
O75369	FLNB		3374900000	3346200000	3915500000	2435400000	2506200000	2212600000	3545533333	2384733333	0.004813641	0.672602147	-0.57217471	
Q14315	FLNC		3112100000	3386300000	3385100000	6422600000	6195500000	6106900000	3294500000	6241666667	2.31028E-05	1.894571761	0.921871786	
O75955	FLOT1		790510000	768990000	730630000	1276700000	912840000	745510000	763376666.7	978350000	0.244702646	1.281608468	0.357955585	
Q14254	FLOT2		864120000	877930000	581090000	709560000	1022500000	1365200000	774380000	1032420000	0.291630061	1.333221416	0.414916397	
P02751	FN1		0	0	0	339940000	250550000	204600000	0	265030000	0.002625493	N/A	N/A	
Q5T0N5	FNBP1L		26072000	15273000	57422000	54958000	45760000	181500000	32922333.33	94072666.67	0.25084286	2.857411889	1.514709012	
Q53EP0	FNDC3B		181050000	129090000	123410000	147680000	193910000	186610000	144516666.7	176066666.7	0.246924878	1.21831392	0.284885917	
Q9BZ67	FRMD8		74701000	100880000	77200000	87429000	116910000	116510000	84260333.33	106949666.7	0.15194221	1.269276567	0.344006458	
Q16658	FSCN1		1539000000	1575600000	1640400000	969470000	842380000	1090700000	1585000000	967516666.7	0.001349726	0.61042061	-0.712124422	
Q12841	FSTL1		390870000	299120000	356750000	59341000	174740000	132930000	348913333.3	122337000	0.006249493	0.350622886	-1.512007926	
Q8IY81	FTSJ3		0	0	0	0	29076000	41497000	0	23524333.33	0.12828942	N/A	N/A	
Q96AE4	FUBP1		76857000	81959000	0	160350000	159110000	98150000	52938666.67	139203333.3	0.061798629	2.629520955	1.394799994	
Q9BWH2	FUNDC2		100800000	164430000	131620000	0	221970000	0	132283333.3	73990000	0.487106727	0.559329722	-0.838229101	
P35637	FUS		320320000	298230000	243150000	214120000	261170000	173570000	287233333.3	216286666.7	0.106422886	0.752999884	-0.409278452	
P51114	FXR1		317830000	375820000	406560000	534090000	728540000	432860000	366736666.7	565163333.3	0.093611807	1.541060343	0.623923354	
Q13283	G3BP1		407360000	447710000	448830000	543160000	643080000	686990000	434633333.3	624410000	0.013193974	1.436636245	0.522694819	
P11413	G6PD		708660000	722100000	745630000	1128900000	901850000	822870000	725463333.3	951206666.7	0.07088821	1.311171251	0.390856128	
P10253	GAA		0	108070000	148940000	242610000	207080000	0	85670000	149896666.7	0.504693338	1.749698455	0.807106308	
P54803	GALC		0	0	47270000	0	0	65124000	15756666.67	21708000	0.835283264	1.37770256	0.46226445	
Q8N428	GALNT16		0	38771000	0	28687000	75299000	0	12923666.67	34662000	0.441394278	2.682056176	1.423339455	
Q10471	GALNT2		886480000	1568400000	1360200000	1449200000	1790200000	1883400000	1271693333	1707600000	0.144877145	1.34277656	0.425219259	
Q14697	GANAB		7768200000	7761400000	7877300000	9166300000	9853700000	9052800000	7802300000	9357600000	0.003551747	1.199338657	0.262239089	
P04406	GAPDH		67507000000	61777000000	58236000000	66194000000	69991000000	86972000000	62506666667	74385666667	0.161912204	1.190043729	0.251014587	
P41250	GARS1		494250000	323890000	434080000	247940000	187150000	469190000	417406666.7	301426666.7	0.307078937	0.722141477	-0.469646588	
P22102	GART		207460000	249120000	60947000	169260000	171360000	371080000	172509000	237233333.3	0.502603421	1.375193951	0.459635104	
P04062	GBA		919020000	858010000	670040000	703370000	550080000	765480000	815690000	672976666.7	0.221130128	0.825039741	-0.277464481	
Q92538	GBF1		68211000	76616000	104470000	80430000	64843000	65604000	83099000	70292333.33	0.34867189	0.845886633	-0.24146377	
Q8IWJ2	GCC2		0	0	0	48540000	77489000	63914000	0	63314333.33	0.001631333	N/A	N/A	
Q92616	GCN1		326920000	217320000	235850000	308780000	324180000	348500000	260030000	327153333.3	0.133973067	1.258136882	0.331288892	
P50395	GDI2		87568000	66722000	0	211360000	317970000	280020000	51430000	269783333.3	0.00591772	5.245641325	2.391119165	
Q8TEQ6	GEMIN5		39821000	0	0	0	0	24787000	13273666.67	8262333.333	0.764621283	0.622460511	-0.68394578	
O43681	GET3		514560000	224510000	301120000	175500000	329280000	675100000	346730000	393293333.3	0.799249404	1.134292773	0.181793064	
Q9UJY4	GGA2		144580000	34172000	0	0	16894000	0	59584000	5631333.333	0.287277256	0.094510831	-3.403376523	
P38435	GGCX		0	0	0	0	123650000	133290000	0	85646666.67	0.116676112	N/A	N/A	
Q8N2G8	GHDC		0	30345000	0	0	22885000	0	10115000	7628333.333	0.853960489	0.754160488	-0.407056529	
Q6Y7W6	GIGYF2		61247000	139050000	0	0	0	206060000	66765666.67	68686666.67	0.981903112	1.028772273	0.040923666	
Q9Y2X7	GIT1		0	59153000	68448000	63406000	74498000	50022000	42533666.67	62642000	0.423371967	1.472762753	0.558525045	
P06280	GLA		215640000	257490000	301000000	0	244870000	341670000	258043333.3	195513333.3	0.582226747	0.757676359	-0.400346361	
P16278	GLB1		228450000	295740000	367600000	348020000	439870000	411100000	297263333.3	399663333.3	0.10221274	1.344475717	0.427043699	
Q92896	GLG1		394520000	438180000	381610000	826620000	742060000	578770000	404770000	715816666.7	0.014117834	1.768452866	0.822487768	
Q9H4G4	GLIPR2		213110000	233920000	331630000	242180000	205660000	149400000	259553333.3	199080000	0.25386352	0.767009992	-0.382682724	
O76003	GLRX3		251130000	269030000	0	951690000	1131600000	0	173386666.7	694430000	0.223102737	4.005094586	2.001836314	
P17900	GM2A		148010000	104290000	38530000	119730000	261420000	181250000	96943333.33	187466666.7	0.156153413	1.933775745	0.951420499	
P29992	GNA11		158890000	114790000	156490000	241000000	242240000	272320000	143390000	251853333.3	0.003520381	1.75642188	0.812639411	
Q03113	GNA12		0	0	50827000	0	68075000	55425000	16942333.33	41166666.67	0.418883384	2.429810927	1.280844057	
Q14344	GNA13		344880000	404080000	416090000	215510000	166760000	239560000	388350000	207276666.7	0.004137928	0.533736749	-0.905799746	
P63096	GNAI1		248000000	206570000	252860000	0	125040000	161470000	235810000	95503333.33	0.051470021	0.405001202	-1.304001907	
P04899	GNAI2		2924700000	2510800000	2491500000	2622700000	2975400000	2969500000	2642333333	2855866667	0.308525912	1.080812413	0.112116149	
P08754	GNAI3		1650300000	1659200000	1614500000	1376300000	1169000000	1283800000	1641333333	1276366667	0.004040571	0.77764013	-0.362825424	
P09471	GNAO1		234690000	258740000	251690000	136270000	183430000	0	248373333.3	106566666.7	0.062831581	0.429058407	-1.220754043	
P63092	GNAS		544260000	506670000	492080000	614850000	661980000	612740000	514336666.7	629856666.7	0.006667919	1.22459997	0.292310553	
P62873	GNB1		3016200000	3173400000	2269100000	2063300000	2138900000	2706600000	2819566667	2302933333	0.208619357	0.816768534	-0.292000807	
P62879	GNB2		4002000000	3404100000	4732000000	4805400000	4209900000	3711600000	4046033333	4242300000	0.713274877	1.048508416	0.068338441	
Q9HAV0	GNB4		233430000	175910000	181490000	0	0	0	196943333.3	0	0.000423952	0	N/A	
P50151	GNG10		0	0	0	0	116940000	97480000	0	71473333.33	0.119382445	N/A	N/A	

Q9UBI6	GNG12		316710000	677200000	397960000	519460000	464440000	173760000	463956666.7	385886666.7	0.636813091	0.831729975	-0.265812869	
P63218	GNG5		16108000	37589000	29592000	0	18251000	8489100	27763000	8913366.667	0.082823808	0.321052	-1.63912111	
P15586	GNS		795300000	848890000	774600000	1381400000	1788400000	1905400000	806263333.3	1691733333	0.005249785	2.098239202	1.069179157	
Q08379	GOLGA2		227300000	235150000	213280000	386140000	312530000	159710000	225243333.3	286126666.7	0.414844638	1.270300268	0.345169555	
Q13439	GOLGA4		79030000	72993000	73473000	296780000	229930000	44528000	75165333.33	190412666.7	0.20148909	2.533251144	1.340990112	
Q8TBA6	GOLGA5		0	0	101680000	107400000	88684000	0	33893333.33	65361333.33	0.543009353	1.928442172	0.947435884	
Q14789	GOLGB1		1930500000	1831600000	1942800000	1688500000	1804900000	1709500000	1901633333	1734300000	0.02904352	0.912005469	-0.132885619	
O00461	GOLIM4		543520000	720110000	651720000	1543400000	1915000000	561380000	638450000	1339926667	0.15992191	2.09871825	1.0695085	
O95249	GOSR1		0	382440000	399000000	491050000	503930000	635680000	260480000	543553333.3	0.11004921	2.086737305	1.061248994	
O14653	GOSR2		47976000	177110000	0	158590000	133350000	0	75028666.67	97313333.33	0.773091276	1.297015363	0.375195568	
O43292	GPAA1		0	0	50996000	0	0	43215000	16998666.67	14405000	0.912941638	0.847419405	-0.238851929	
P35052	GPC1		1950400000	1913300000	2487800000	1831900000	1950100000	1981600000	2117166667	1921200000	0.363188886	0.907439188	-0.140127131	
O75487	GPC4		142760000	199180000	249210000	248640000	150390000	65254000	197050000	154761333.3	0.527960051	0.785391187	-0.348516686	
Q9Y625	GPC6		85696000	0	0	107720000	123180000	80294000	28565333.33	103731333.3	0.073599807	3.631371359	1.860514474	
Q8NFJ5	GPRC5A		220650000	217540000	301150000	259400000	262650000	230320000	246446666.7	250790000	0.889076386	1.017623827	0.025204355	
Q8TD30	GPT2		127800000	169800000	178090000	163090000	166090000	148130000	158563333.3	159103333.3	0.975501639	1.003405579	0.004904865	
P07203	GPX1		190450000	219930000	152410000	661850000	581570000	444290000	187596666.7	562570000	0.004857771	2.998827271	1.584398427	
Q8TED1	GPX8		202130000	280140000	278310000	0	0	0	253526666.7	0	0.000592726	0	N/A	
Q9UBQ7	GRHPR		654690000	590040000	665930000	681160000	649630000	583050000	636886666.7	637946666.7	0.978721588	1.001664346	0.002399148	
P28799	GRN		196670000	0	0	423720000	348460000	0	65556666.67	257393333.3	0.25930654	3.926272436	1.973160282	
Q12849	GRSF1		2641200000	2451200000	2305700000	1250000000	774870000	1334000000	2466033333	1119623333	0.002505444	0.454017924	-1.139178842	
P49841	GSK3B		108340000	122580000	77936000	77775000	104190000	97463000	102952000	93142666.67	0.557981787	0.904719351	-0.144457765	
Q9Y2Q3	GSTK1		651360000	509020000	669270000	827340000	667490000	615950000	609883333.3	703593333.3	0.31357322	1.153652338	0.206208522	
P09211	GSTP1		178780000	218420000	154920000	992960000	858650000	630050000	184040000	827220000	0.003928517	4.494783743	2.168251705	
O43708	GSTZ1		291320000	247550000	291920000	110580000	69811000	0	276930000	60130333.33	0.003627954	0.217131887	-2.203356489	
Q4G148	GXYLT1		0	76115000	91737000	155760000	190570000	92718000	55950666.67	146349333.3	0.088240911	2.615685247	1.387188947	
P07305	H1		62797000	0	0	116720000	132420000	0	20932333.33	83046666.67	0.254448764	3.967386977	1.988189124	
Q02539	H1		296000000	189980000	503600000	366860000	373930000	194230000	329860000	311673333.3	0.875863929	0.944865498	-0.081819118	
Q92522	H1		176420000	290160000	346150000	0	0	0	270910000	0	0.005596763	0	N/A	
P16403	H1		74152000	0	266910000	540580000	276080000	211560000	113687333.3	342740000	0.148752195	3.01475978	1.592043051	
P10412	H1		364260000	530160000	554210000	1522900000	1011400000	584510000	482876666.7	1039603333	0.115533946	2.152937603	1.106306508	
Q99878	H2AC14		8009900000	6980600000	7842200000	15283000000	10428000000	7749300000	7610900000	11153433333	0.18700198	1.465455246	0.55134891	
Q16777	H2AC20		178270000	0	469450000	0	202780000	373630000	215906666.7	192136666.7	0.898114676	0.889906132	-0.168274927	
Q93077	H2AC6		161320000	221100000	111410000	148390000	0	0	164610000	49463333.33	0.121579284	0.300488022	-1.73462061	
P16104	H2AX		392400000	304200000	228130000	275600000	232450000	267150000	308243333.3	258400000	0.368896494	0.838298747	-0.254463622	
Q71UI9	H2AZ2		620000000	487870000	1002400000	1359900000	1012400000	1576800000	703423333.3	1316366667	0.053045243	1.87137191	0.904096303	
Q99879	H2BC14		4078600000	5418000000	6006700000	15245000000	12217000000	6230500000	5167766667	11230833333	0.088832024	2.173246986	1.119852144	
Q16778	H2BC21		0	216740000	325500000	714670000	605520000	401200000	180746666.7	573796666.7	0.041418907	3.174590587	1.666570546	
P57053	H2BS1		0	0	0	64296000	49088000	0	0	37794666.67	0.123222872	N/A	N/A	
P84243	H3		63926000	55263000	0	360010000	593040000	218330000	39729666.67	390460000	0.034244591	9.827920362	3.296886167	
P68431	H3C1		233470000	294680000	184830000	545410000	301510000	154460000	237660000	333793333.3	0.46221517	1.404499425	0.490056034	
Q71DI3	H3C15		0	0	0	207240000	170610000	252140000	0	209996666.7	0.000878099	N/A	N/A	
P62805	H4C1		2300200000	2448800000	2638300000	4382700000	4475600000	3044300000	2462433333	3967533333	0.03339112	1.611224669	0.688157678	
P12081	HARS1		0	0	0	75451000	72003000	0	0	49151333.33	0.116333964	N/A	N/A	
O00165	HAX1		335600000	264030000	327390000	162870000	224250000	161700000	309006666.7	182940000	0.014659757	0.592026062	-0.756267408	
Q9BXW7	HDHD5		0	232740000	206150000	208820000	273150000	274420000	146296666.7	252130000	0.239607696	1.72341589	0.785270891	
Q00341	HDLBP		512670000	423830000	453530000	999230000	1118900000	1254200000	463343333.3	1124110000	0.001071607	2.426084329	1.278629698	
Q9H583	HEATR1		52100000	0	0	0	31162000	22421000	17366666.67	17861000	0.981173747	1.028464491	0.040491984	
Q9NRV9	HEBP1		279200000	276430000	372760000	163520000	127190000	137280000	309463333.3	142663333.3	0.007569166	0.46100238	-1.117153895	
P06865	HEXA		133150000	0	132460000	84726000	232550000	368880000	88536666.67	228718666.7	0.20711752	2.583321411	1.369227152	
P07686	HEXB		283400000	199480000	255990000	205180000	250290000	362040000	246290000	272503333.3	0.645400437	1.106432796	0.145915825	
O14964	HGS		0	0	40903000	194440000	134860000	0	13634333.33	109766666.7	0.179202158	8.050754223	3.009123946	
O00291	HIP1		156470000	124070000	166590000	102720000	0	72684000	149043333.3	58468000	0.0520069	0.392288596	-1.350012698	
O75146	HIP1R		146090000	262620000	110290000	204090000	380820000	191780000	173000000	258896666.7	0.32403483	1.496512524	0.581604353	
P19367	HK1		2923100000	2781600000	2869100000	2395700000	2765900000	2219900000	2857933333	2460500000	0.074956274	0.860936807	-0.216020747	
P10321	HLA		0	0	0	17978000	7965300	0	0	8647766.667	0.171704695	N/A	N/A	
P17096	HMGA1		0	0	0	229440000	0	158070000	0	129170000	0.129430428	N/A	N/A	

P09429	HMGB1		729480000	737720000	645190000	1019100000	1075400000	474020000	704130000	856173333.3	0.477075461	1.21593077	0.282061091	
P26583	HMGB2		194910000	312970000	290180000	364350000	277950000	100640000	266020000	247646666.7	0.840597636	0.930932511	-0.103251513	
P30519	HMOX2		563890000	491420000	460390000	444720000	513630000	584880000	505233333.3	514410000	0.865352953	1.018163225	0.025968863	
Q13151	HNRNPA0		171240000	117560000	123160000	94482000	131270000	138950000	137320000	121567333.3	0.511308443	0.885284979	-0.175786152	
P09651	HNRNPA1		683670000	319200000	182830000	434390000	579830000	540130000	395233333.3	518116666.7	0.474041182	1.310913384	0.390572366	
P22626	HNRNPA2B1		1630700000	1832100000	1380000000	2686000000	1479600000	1520100000	1614266667	1895233333	0.537020337	1.174052201	0.231496556	
P51991	HNRNPA3		295790000	260360000	334100000	483610000	351060000	299150000	296750000	377940000	0.240157832	1.273597304	0.348909187	
Q99729	HNRNPAB		0	57520000	0	129700000	169440000	0	19173333.33	99713333.33	0.214448539	5.200625869	2.378685255	
P07910	HNRNPC		428980000	293540000	365020000	467700000	581290000	506960000	362513333.3	518650000	0.038439056	1.430705997	0.516727236	
Q14103	HNRNPD		1024200000	1040800000	1254700000	886900000	959590000	976360000	1106566667	940950000	0.104500553	0.850332861	-0.233900402	
O14979	HNRNPDL		286180000	261130000	361800000	512700000	553010000	531450000	303036666.7	532386666.7	0.002107534	1.756839106	0.812982073	
P52597	HNRNPF		308170000	95338000	197140000	121020000	0	356030000	200216000	159016666.7	0.751105451	0.79422557	-0.332379286	
P31943	HNRNPH1		771840000	420580000	546270000	524800000	387720000	580330000	579563333.3	497616666.7	0.52436538	0.858606192	-0.219931518	
P31942	HNRNPH3		0	169070000	132220000	192710000	140010000	169760000	100430000	167493333.3	0.27865562	1.667761957	0.737913385	
P61978	HNRNPK		2058000000	1712400000	1679000000	2773700000	2378100000	1987000000	1816466667	2379600000	0.093921791	1.310015782	0.389584192	
P14866	HNRNPL		269550000	288840000	335090000	374930000	379450000	403930000	297826666.7	386103333.3	0.014623568	1.296402829	0.374514074	
P52272	HNRNPM		994060000	955410000	824800000	1021900000	956880000	1021900000	924756666.7	1000226667	0.246240974	1.081610658	0.113181273	
O43390	HNRNPR		0	205440000	187790000	0	249840000	365890000	131076666.7	205243333.3	0.588857055	1.565826615	0.646924471	
Q00839	HNRNPU		1149900000	1009900000	1112600000	1336200000	1409600000	1269400000	1090800000	1338400000	0.013140109	1.226989366	0.295122745	
Q9BUJ2	HNRNPUL1		152920000	262370000	212300000	70228000	73688000	0	209196666.7	47972000	0.01535008	0.229315317	-2.124595371	
Q9Y251	HPSE		83044000	0	66318000	46617000	51019000	69221000	49787333.33	55619000	0.835284516	1.117131533	0.159799061	
P01112	HRAS		318710000	345230000	284940000	370870000	367190000	445300000	316293333.3	394453333.3	0.064431861	1.247112385	0.318591481	
Q7LGA3	HS2ST1		99232000	0	115880000	0	0	0	71704000	0	0.118493466	0	N/A	
Q53GQ0	HSD17B12		0	0	0	157620000	177300000	166500000	0	167140000	7.9978E-06	N/A	N/A	
P51659	HSD17B4		3149600000	2453700000	2829100000	1755900000	1895800000	2012700000	2810800000	1888133333	0.012602887	0.671742327	-0.574020157	
Q3SXM5	HSDL1		62992000	37203000	46521000	77971000	117210000	78745000	48905333.33	91308666.67	0.047377768	1.867049265	0.900759996	
Q6YN16	HSDL2		629580000	597490000	404870000	443440000	410000000	419520000	543980000	424320000	0.166599995	0.780028678	-0.35840093	
P07900	HSP90AA1		637180000	767500000	782020000	1131600000	1310200000	1211400000	728900000	1217733333	0.002118722	1.670645265	0.740405432	
P08238	HSP90AB1		4074400000	4272500000	4519400000	7117200000	7766100000	7473500000	4288766667	7452266667	0.00015517	1.737624647	0.797116472	
Q58FF8	HSP90AB2P		183710000	151990000	139320000	235790000	297440000	0	158340000	177743333.3	0.842585106	1.122542209	0.166769693	
P14625	HSP90B1		25351000000	24275000000	26088000000	18632000000	20451000000	21094000000	25238000000	20059000000	0.004631323	0.794793565	-0.331347902	
P48723	HSPA13		312320000	289240000	329790000	272310000	0	342310000	310450000	204873333.3	0.371831754	0.659923767	-0.599628719	
P0DMV9	HSPA1B		1916700000	1879200000	1900700000	2349400000	2585100000	2516100000	1898866667	2483533333	0.001173158	1.30790296	0.387255504	
P34932	HSPA4		76828000	203170000	332650000	641360000	774260000	1089000000	204216000	834873333.3	0.014236825	4.088187671	2.031461426	
P11021	HSPA5		31020000000	29863000000	31673000000	36355000000	36370000000	31847000000	30852000000	34857333333	0.066029248	1.129824106	0.176098188	
P17066	HSPA6		720650000	1368300000	827290000	962500000	481350000	214340000	972080000	552730000	0.23060885	0.568605465	-0.814500131	
P11142	HSPA8		12572000000	12891000000	12854000000	12778000000	13346000000	12861000000	12772333333	12995000000	0.335906299	1.017433515	0.024934524	
P04792	HSPB1		1609700000	1903500000	1383800000	3470300000	3631200000	3336700000	1632333333	3479400000	0.000434654	2.131549929	1.091902849	
Q92598	HSPH1		140520000	197590000	196010000	326000000	374710000	298360000	178040000	333023333.3	0.006025477	1.870497267	0.903421858	
Q9BUP3	HTATIP2		0	114990000	80903000	147280000	218670000	262350000	65297666.67	209433333.3	0.039409764	3.207363203	1.681387735	
Q7Z6Z7	HUWE1		37693000	150730000	76462000	121490000	31408000	109500000	88295000	87466000	0.985727347	0.99061102	-0.013609425	
Q9Y4L1	HYOU1		2382100000	2533700000	2637600000	2794000000	2699800000	3851900000	2517800000	3115233333	0.187942767	1.237283872	0.307176538	
P41252	IARS1		506540000	444130000	436920000	364250000	400200000	490630000	462530000	418360000	0.368441171	0.904503492	-0.144802023	
Q9Y6M1	IGF2BP2		649920000	725340000	596210000	1021700000	1032000000	862840000	657156666.7	972180000	0.008977507	1.47937326	0.564986104	
O00425	IGF2BP3		0	135220000	72919000	122610000	47893000	164440000	69379666.67	111647666.7	0.460743955	1.609227487	0.686368286	
P11717	IGF2R		160320000	165820000	154750000	194610000	190170000	182780000	160296666.7	189186666.7	0.003560522	1.180228327	0.23906599	
Q969P0	IGSF8		190580000	160160000	125660000	163330000	168620000	82506000	158800000	138152000	0.571968421	0.869974811	-0.200954465	
Q14116	IL18		0	0	0	51104000	60484000	24999000	0	45529000	0.01275968	N/A	N/A	
Q12905	ILF2		209980000	148470000	0	0	127500000	0	119483333.3	42500000	0.365185399	0.355698145	-1.491274645	
Q12906	ILF3		79724000	114900000	129110000	187930000	126350000	56777000	107911333.3	123685666.7	0.717602444	1.146178653	0.196831932	
Q13418	ILK		119810000	108240000	117500000	51435000	77917000	56870000	115183333.3	62074000	0.003824494	0.538914774	-0.891870958	
A1L0T0	ILVBL		386790000	197160000	311210000	291530000	339880000	386600000	298386666.7	339336666.7	0.54238213	1.137238036	0.185534257	
P12268	IMPDH2		1347800000	1570100000	1467300000	1561900000	1865800000	1510100000	1461733333	1645933333	0.224123847	1.126014777	0.17122576	
Q8TEX9	IPO4		0	0	56645000	84128000	115160000	129730000	18881666.67	109672666.7	0.017296039	5.808420867	2.538145992	
O00410	IPO5		662510000	718360000	847660000	773640000	1158900000	664500000	742843333.3	865680000	0.484609694	1.165360125	0.220775852	
Q96P70	IPO9		263440000	253470000	120050000	304490000	256370000	328400000	212320000	296420000	0.173470705	1.396100226	0.481402516	
P46940	IQGAP1		1211400000	1285300000	1208100000	362520000	116370000	117570000	1234933333	198820000	0.000267765	0.160996545	-2.634898366	

Q96AB3	ISOC2		0	0	0	49591000	106890000	0	0	52160333.33	0.16649698	N/A	N/A	
P17301	ITGA2		212870000	202700000	49129000	315810000	220200000	379750000	154899666.7	305253333.3	0.09953165	1.97065197	0.97867301	
P26006	ITGA3		178240000	220080000	295480000	524640000	466620000	662870000	231266666.7	551376666.7	0.009051492	2.3841597	1.253480876	
P08648	ITGA5		305660000	326500000	411510000	406900000	497320000	446030000	347890000	450083333.3	0.070105413	1.293751856	0.371560933	
P23229	ITGA6		264650000	337860000	261600000	546230000	542380000	522700000	288036666.7	537103333.3	0.000660644	1.864704725	0.898947199	
P06756	ITGAV		1385900000	1539900000	1329400000	1384200000	1476600000	1179400000	1418400000	1346733333	0.543319157	0.949473585	-0.07480023	
P05556	ITGB1		4702100000	4787400000	4391500000	4004000000	3939100000	3092900000	4627000000	3678666667	0.04034244	0.795043585	-0.330894143	
P05106	ITGB3		0	0	0	62849000	0	27503000	0	30117333.33	0.173122163	N/A	N/A	
P16144	ITGB4		138310000	118350000	113810000	0	0	0	123490000	0	8.07192E-05	0	N/A	
P18084	ITGB5		610530000	499960000	666510000	799620000	852430000	932520000	592333333.3	861523333.3	0.012469514	1.45445695	0.540480595	
Q9NZM3	ITSN2		0	0	31443000	0	0	96849000	10481000	32283000	0.555619875	3.080145024	1.62299828	
P14923	JUP		324950000	303250000	315050000	108820000	166480000	247580000	314416666.7	174293333.3	0.026291526	0.554338723	-0.851160306	
Q63ZY3	KANK2		50441000	37828000	32642000	0	0	0	40303666.67	0	0.00158774	0	N/A	
Q15046	KARS1		1000300000	1063200000	1018900000	1290300000	1142500000	1276600000	1027466667	1236466667	0.014591242	1.203412925	0.267131757	
Q06136	KDSR		0	31513000	0	0	58104000	0	10504333.33	19368000	0.708042758	1.843810491	0.882690381	
Q07666	KHDRBS1		0	0	79621000	67417000	0	61681000	26540333.33	43032666.67	0.654900851	1.621406413	0.697245754	
Q92945	KHSRP		136840000	116550000	0	120380000	164420000	166710000	84463333.33	150503333.3	0.21798658	1.781877738	0.833398351	
Q9P206	KIAA1522		62486000	0	63528000	0	0	0	42004666.67	0	0.116143719	0	N/A	
Q8IYS2	KIAA2013		141560000	150850000	165530000	173270000	204390000	148480000	152646666.7	175380000	0.266402791	1.148927807	0.200288149	
Q9ULH0	KIDINS220		354210000	157710000	302480000	242810000	161190000	314450000	271466666.7	239483333.3	0.686335771	0.882183202	-0.180849805	
P52732	KIF11		76781000	112610000	101680000	0	0	0	97023666.67	0	0.000791309	0	N/A	
Q02241	KIF23		121710000	166610000	119250000	99177000	0	0	135856666.7	33059000	0.047880573	0.243337341	-2.038970372	
O00139	KIF2A		0	54735000	0	0	0	44697000	18245000	14899000	0.893909722	0.81660729	-0.292285649	
O95239	KIF4A		0	59023000	0	0	43992000	0	19674333.33	14664000	0.848175588	0.745336564	-0.42403606	
P33176	KIF5B		402860000	391000000	290330000	487960000	415670000	363830000	361396666.7	422486666.7	0.294599978	1.169038637	0.225322612	
P52292	KPNA2		244320000	230780000	284920000	176730000	167460000	186310000	253340000	176833333.3	0.011157445	0.698007947	-0.518684633	
O60684	KPNA6		56420000	0	40215000	0	0	0	32211666.67	0	0.127179877	0	N/A	
Q14974	KPNB1		700410000	676080000	727520000	628380000	655700000	603760000	701336666.7	629280000	0.026953226	0.897258093	-0.156405064	
Q13601	KRR1		0	0	0	0	108000000	108100000	0	72033333.33	0.116116609	N/A	N/A	
Q86UP2	KTN1		6112300000	5782400000	5907500000	6820900000	6742000000	5441300000	5934066667	6334733333	0.430608483	1.067519745	0.094262754	
Q53H82	LACTB2		0	0	91079000	84754000	0	0	30359666.67	28251333.33	0.9618915	0.930554793	-0.103836993	
P55268	LAMB2		126030000	146440000	154890000	0	0	0	142453333.3	0	7.66017E-05	0	N/A	
Q13751	LAMB3		555750000	441340000	319990000	217430000	288130000	174890000	439026666.7	226816666.7	0.048567333	0.516635284	-0.952781918	
P11047	LAMC1		97529000	112190000	63170000	135050000	150240000	128110000	90963000	137800000	0.042372401	1.514901663	0.599224147	
Q13753	LAMC2		0	0	0	101660000	0	30234000	0	43964666.67	0.218397792	N/A	N/A	
P11279	LAMP1		881170000	683740000	883850000	378550000	736600000	832380000	816253333.3	649176666.7	0.336701139	0.79531273	-0.330405832	
Q6IAA8	LAMTOR1		976440000	900760000	945440000	1687700000	1818300000	2506400000	940880000	2004133333	0.01401614	2.130062636	1.090895855	
Q9Y2Q5	LAMTOR2		264900000	231750000	69135000	68719000	404860000	385580000	188595000	286386333.3	0.47655292	1.518525588	0.602671219	
Q9UHA4	LAMTOR3		740660000	506560000	0	1159300000	1335300000	1530200000	415740000	1341600000	0.019044044	3.227016886	1.690201127	
Q0VGL1	LAMTOR4		229120000	374320000	330970000	143920000	142680000	692400000	311470000	326333333.3	0.940789654	1.047719952	0.067253146	
O43504	LAMTOR5		641170000	804170000	618150000	1346800000	986320000	720950000	687830000	1018023333	0.158202375	1.480050788	0.565646683	
P28838	LAP3		995950000	927560000	884410000	899630000	1041400000	1239400000	935973333.3	1060143333	0.297430786	1.132664036	0.179720001	
Q6PKG0	LARP1		0	0	0	61530000	36666000	0	0	32732000	0.140966353	N/A	N/A	
Q9P2J5	LARS1		249920000	275730000	305370000	228280000	303110000	329750000	277006666.7	287046666.7	0.784534304	1.036244615	0.051364605	
Q14847	LASP1		60503000	47106000	45619000	76207000	35039000	16383000	51076000	42543000	0.665196053	0.832935234	-0.263723774	
Q9GZY6	LAT2		0	0	0	69732000	61547000	0	0	43759666.67	0.117660419	N/A	N/A	
Q6UX15	LAYN		132190000	106940000	0	0	45116000	0	79710000	15038666.67	0.208878931	0.188667252	-2.406084066	
Q14739	LBR		368250000	0	314640000	216360000	148860000	245620000	227630000	203613333.3	0.849134396	0.894492524	-0.16085867	
Q6UWP7	LCLAT1		67350000	64334000	51221000	86755000	0	89612000	60968333.33	58789000	0.945248548	0.964254668	-0.05251387	
P00338	LDHA		141290000	130080000	0	271960000	230210000	181330000	90456666.67	227833333.3	0.058583699	2.518701404	1.332680098	
P07195	LDHB		61391000	0	36481000	210970000	162060000	80650000	32624000	151226666.7	0.047578637	4.63544221	2.212706975	
P01130	LDLR		77860000	49478000	48395000	0	0	0	58577666.67	0	0.003714953	0	N/A	
P09382	LGALS1		7576900000	7532500000	7382800000	9115300000	9426000000	8565200000	7497400000	9035500000	0.003999347	1.205151119	0.269214064	
Q99538	LGMN		161430000	109840000	139440000	274670000	168610000	104340000	136903333.3	182540000	0.42856666	1.333349565	0.415055063	
Q9UHB6	LIMA1		1350600000	1183500000	1099200000	1254300000	1012000000	746890000	1211100000	1004396667	0.276280725	0.829325957	-0.269988847	
P48059	LIMS1		0	84311000	75813000	45477000	91114000	60748000	53374666.67	65779666.67	0.700156183	1.232413629	0.301486543	
Q9NUP9	LIN7C		498470000	737350000	962680000	496130000	504450000	449880000	732833333.3	483486666.7	0.138677832	0.659749829	-0.600009022	

Q15334	LLGL1		90625000	102140000	124240000	132600000	126780000	118890000	105668333.3	126090000	0.127182535	1.193261936	0.254910767	
P49257	LMAN1		951020000	999060000	756390000	635850000	665500000	450150000	902156666.7	583833333.3	0.033662622	0.647152934	-0.627821408	
Q9BU23	LMF2		65924000	50605000	38505000	53671000	56055000	0	51678000	36575333.33	0.491100383	0.707754428	-0.498679225	
P02545	LMNA		442060000	444120000	447550000	933370000	698300000	749260000	444576666.7	793643333.3	0.008113843	1.785166413	0.836058568	
P20700	LMNB1		335000000	301990000	564190000	695650000	491240000	372970000	400393333.3	519953333.3	0.393762398	1.29860637	0.376964192	
Q03252	LMNB2		62296000	79799000	63649000	65571000	0	93599000	68581333.33	53056666.67	0.612464536	0.773631309	-0.370281913	
Q8NF37	LPCAT1		56822000	129900000	42576000	47608000	68909000	77306000	76432666.67	64607666.67	0.699057916	0.845288664	-0.242483993	
Q96II8	LRCH3		69293000	37981000	76133000	0	0	0	61135666.67	0	0.00649175	0	N/A	
O75427	LRCH4		0	0	0	40209000	16552000	0	0	18920333.33	0.180204077	N/A	N/A	
Q07954	LRP1		487020000	465600000	493180000	657660000	551630000	500640000	481933333.3	569976666.7	0.134297878	1.182687785	0.242069271	
P30533	LRPAP1		607650000	633270000	658870000	802700000	504120000	337870000	633263333.3	548230000	0.567849488	0.865722001	-0.208024271	
Q9BTT6	LRRC1		142230000	74086000	52355000	172030000	216960000	144550000	89557000	177846666.7	0.061847676	1.985848863	0.989755828	
Q9H9A6	LRRC40		0	0	0	57598000	47281000	64720000	0	56533000	0.000365986	N/A	N/A	
Q8N1G4	LRRC47		0	45486000	105820000	40500000	26849000	74606000	50435333.33	47318333.33	0.930913561	0.938198089	-0.092035533	
Q96AG4	LRRC59		12521000000	12004000000	11810000000	8734100000	9338700000	10088000000	12111666667	9386933333	0.00361503	0.775032338	-0.367671587	
Q6NSJ5	LRRC8E		0	0	0	37029000	22792000	62951000	0	40924000	0.025321847	N/A	N/A	
Q9H089	LSG1		197300000	208390000	202830000	235460000	224570000	270690000	202840000	243573333.3	0.046293554	1.200815092	0.264014015	
Q9Y4Y9	LSM5		0	0	0	0	30618000	163510000	0	64709333.33	0.266759151	N/A	N/A	
Q86X29	LSR		184050000	139810000	198020000	272760000	263240000	228060000	173960000	254686666.7	0.022019863	1.464053039	0.54996782	
P48449	LSS		358580000	401700000	330190000	309680000	368840000	393070000	363490000	357196666.7	0.855168617	0.982686365	-0.025197057	
O94822	LTN1		0	0	0	33899000	59357000	0	0	31085333.33	0.144871965	N/A	N/A	
Q9NQ29	LUC7L		0	0	0	0	19455000	18160000	0	12538333.33	0.116587752	N/A	N/A	
Q9Y383	LUC7L2		48445000	49105000	46670000	64141000	53976000	46654000	48073333.33	54923666.67	0.252073929	1.142497573	0.192191101	
Q9HD34	LYRM4		194730000	222850000	0	121120000	113700000	0	139193333.3	78273333.33	0.490233969	0.562335361	-0.830497325	
Q5U5X0	LYRM7		356940000	435300000	383560000	550350000	480030000	212400000	391933333.3	414260000	0.842728845	1.05696547	0.079928247	
P20645	M6PR		115910000	88076000	71309000	192080000	99320000	132890000	91765000	141430000	0.173994966	1.541219419	0.624072269	
Q9UPN3	MACF1		898860000	896760000	858390000	644870000	642420000	504210000	884670000	597166666.7	0.004000089	0.675016296	-0.567005763	
O75367	MACROH2A1		139810000	0	125410000	119040000	132480000	0	88406666.67	83840000	0.944086723	0.94834477	-0.076516449	
P43358	MAGEA4		278440000	251970000	280490000	672030000	786570000	833670000	270300000	764090000	0.000539766	2.82682205	1.499181067	
O15479	MAGEB2		96078000	94646000	104640000	0	0	0	98454666.67	0	6.01198E-06	0	N/A	
Q9UNF1	MAGED2		145150000	164570000	198480000	339270000	325080000	246250000	169400000	303533333.3	0.015078435	1.791814246	0.841421084	
Q9H0U3	MAGT1		41419000	83291000	40846000	0	0	18876000	55185333.33	6292000	0.033686313	0.114015801	-3.132694315	
P33908	MAN1A1		0	64686000	0	8133800	5659900	0	21562000	4597900	0.47796652	0.213240887	-2.229444008	
Q9UKM7	MAN1B1		0	0	0	100150000	77819000	73359000	0	83776000	0.000538983	N/A	N/A	
Q16706	MAN2A1		272640000	301310000	216600000	126460000	196040000	94522000	263516666.7	139007333.3	0.033002001	0.527508696	-0.922733217	
O00754	MAN2B1		138020000	0	80376000	168790000	153370000	180850000	72798666.67	167670000	0.080694198	2.303201524	1.203640649	
Q9NQG1	MANBAL		0	0	57286000	41895000	0	0	19095333.33	13965000	0.838926652	0.731330517	-0.451404531	
P55145	MANF		320600000	383930000	641580000	564670000	726350000	382700000	448703333.3	557906666.7	0.477769313	1.243375356	0.314261889	
Q02750	MAP2K1		0	0	0	106480000	80951000	0	0	62477000	0.123442993	N/A	N/A	
P27816	MAP4		333800000	132080000	235790000	281910000	379250000	697540000	233890000	452900000	0.188612651	1.93638035	0.953362359	
O95819	MAP4K4		0	0	0	65887000	63611000	0	0	43166000	0.116239374	N/A	N/A	
Q15691	MAPRE1		171330000	175240000	141030000	141090000	205610000	314270000	162533333.3	220323333.3	0.326046656	1.355557834	0.438886666	
P49006	MARCKSL1		847020000	807110000	648070000	532950000	403420000	297880000	767400000	411416666.7	0.017478383	0.536117627	-0.899378526	
P56192	MARS1		79333000	0	89228000	154390000	209960000	238980000	56187000	201110000	0.018224754	3.579297702	1.839676543	
P43243	MATR3		271280000	277560000	240780000	306880000	343440000	365100000	263206666.7	338473333.3	0.02114954	1.285960335	0.362846144	
Q96N66	MBOAT7		231040000	205550000	252080000	347280000	244790000	258880000	229556666.7	283650000	0.194860886	1.235642615	0.305261533	
P43121	MCAM		467090000	446160000	464980000	447590000	482480000	368600000	459410000	432890000	0.483005248	0.942273786	-0.085781786	
P25205	MCM3		888360000	704960000	642700000	341370000	398630000	468320000	745340000	402773333.3	0.014155262	0.540388726	-0.887930517	
P33992	MCM5		184920000	135150000	214830000	0	0	118680000	178300000	39560000	0.039011961	0.221873247	-2.172192372	
P40925	MDH1		0	0	0	162070000	0	205110000	0	122393333.3	0.121552955	N/A	N/A	
Q6P9B6	MEAK7		27763000	0	72934000	69991000	23572000	81020000	33565666.67	58194333.33	0.422570805	1.733745792	0.793892381	
P08582	MELTF		379570000	350650000	415930000	338050000	329240000	359260000	382050000	342183333.3	0.128865094	0.895650657	-0.158991968	
Q14696	MESD		242680000	0	278560000	357490000	199100000	359860000	173746666.7	305483333.3	0.267649705	1.758211189	0.814108371	
P53582	METAP1		0	0	0	100290000	122550000	107900000	0	110246666.7	7.22467E-05	N/A	N/A	
P50579	METAP2		93934000	0	79594000	125240000	104010000	91891000	57842666.67	107047000	0.185371153	1.850658107	0.888038394	
Q08431	MFGE8		0	0	33718000	35927000	78248000	0	11239333.33	38058333.33	0.34808678	3.386173557	1.75965592	
P26572	MGAT1		0	0	0	339680000	638960000	618020000	0	532220000	0.005267228	N/A	N/A	

Q10469	MGAT2		0	0	0	54821000	66035000	0	0	40285333.33	0.119529813	N/A	N/A	
O14880	MGST3		510630000	510790000	668890000	181160000	220040000	351300000	563436666.7	250833333.3	0.013237775	0.445184611	-1.167524372	
Q5JRA6	MIA3		221640000	128350000	196290000	423480000	541240000	399380000	182093333.3	454700000	0.006297694	2.497071099	1.3202369	
O94851	MICAL2		0	39490000	0	0	38779000	0	13163333.33	12926333.33	0.990365632	0.981995442	-0.026211767	
L0R8F8	MIEF1		47069000	52681000	70713000	64034000	49973000	0	56821000	38002333.33	0.414664061	0.668807894	-0.58033622	
P14174	MIF		0	0	0	401020000	0	584900000	0	328640000	0.129764646	N/A	N/A	
Q9UNW1	MINPP1		192700000	273600000	152500000	623800000	461820000	586430000	206266666.7	557350000	0.004398836	2.70208468	1.434072888	
Q8IVT2	MISP		0	118880000	178920000	0	0	0	99266666.67	0	0.132026776	0	N/A	
Q14165	MLEC		24192000	44355000	0	0	83098000	45273000	22849000	42790333.33	0.504541047	1.872744248	0.905153891	
Q8N4V1	MMGT1		139380000	160110000	174420000	0	161390000	141030000	157970000	100806666.7	0.331338921	0.638138043	-0.648059551	
Q96T76	MMS19		33065000	34707000	0	0	57234000	22996000	22590666.67	26743333.33	0.846466019	1.183822227	0.24345245	
Q9H8S9	MOB1A		0	0	20464000	0	0	53598000	6821333.333	17866000	0.594530994	2.619136044	1.389090998	
Q13724	MOGS		1129400000	1127700000	1091400000	1261500000	1338700000	1303900000	1116166667	1301366667	0.001917619	1.165925041	0.221475039	
Q00013	MPP1		91856000	94973000	0	0	0	0	62276333.33	0	0.116227223	0	N/A	
Q14168	MPP2		0	22195000	217300000	0	30167000	61926000	79831666.67	30697666.67	0.528693905	0.384529948	-1.378832131	
Q9NZW5	MPP6		332970000	307310000	309430000	236350000	0	190720000	316570000	142356666.7	0.075060268	0.449684641	-1.153014487	
Q5T2T1	MPP7		59106000	68758000	51547000	0	0	0	59803666.67	0	0.000275774	0	N/A	
Q6WCQ1	MPRIP		29283000	42084000	49032000	64686000	48396000	38820000	40133000	50634000	0.331504451	1.261654997	0.335317456	
P25325	MPST		448490000	568170000	296290000	204500000	129740000	307040000	437650000	213760000	0.07577676	0.488426825	-1.033785658	
O95297	MPZL1		0	0	0	143970000	97873000	0	0	80614333.33	0.130362484	N/A	N/A	
Q9UBG0	MRC2		332560000	386250000	431720000	460200000	361640000	369510000	383510000	397116666.7	0.765809499	1.035479301	0.050298714	
P52701	MSH6		0	0	0	0	56836000	140300000	0	65712000	0.182079398	N/A	N/A	
P26038	MSN		3053100000	3212900000	3957000000	8208500000	7082000000	5133700000	3407666667	6808066667	0.022432175	1.997867554	0.998460944	
Q86UE4	MTDH		422800000	541180000	444650000	428360000	317990000	265420000	469543333.3	337256666.7	0.093040365	0.718265265	-0.477411346	
P11586	MTHFD1		1863800000	1814300000	1732600000	3682500000	3475100000	3062400000	1803566667	3406666667	0.001000731	1.888849872	0.917508039	
P42285	MTREX		43040000	24967000	44387000	0	0	47517000	37464666.67	15839000	0.273018241	0.422771678	-1.242049362	
Q14764	MVP		350010000	341810000	311040000	285270000	351200000	389350000	334286666.7	341940000	0.826087345	1.022894522	0.032657386	
Q96S97	MYADM		72523000	0	0	0	0	106610000	24174333.33	35536666.67	0.804561	1.470016409	0.555832259	
Q969H8	MYDGF		1144200000	1028400000	1054800000	1917000000	1397300000	1176400000	1075800000	1496900000	0.131093887	1.391429634	0.476567952	
P35580	MYH10		1093100000	961680000	1235900000	1128900000	1112400000	926120000	1096893333	1055806667	0.708906694	0.962542696	-0.055077558	
P35579	MYH9		17475000000	19843000000	17143000000	23593000000	21036000000	15050000000	18153666667	19893000000	0.550358011	1.095811682	0.131999888	
P05976	MYL1		0	209830000	272840000	0	163500000	0	160890000	54500000	0.342414358	0.338740755	-1.561746524	
P19105	MYL12A		1011400000	900190000	944340000	823190000	947580000	1388500000	951976666.7	1053090000	0.593410782	1.106214088	0.14563062	
P60660	MYL6		3373000000	3503900000	3701100000	2351600000	2684000000	2227900000	3526000000	2421166667	0.002661464	0.686660995	-0.542330081	
Q92614	MYO18A		41426000	43597000	85647000	127940000	73588000	88992000	56890000	96840000	0.138744814	1.702232378	0.767427998	
Q96H55	MYO19		0	0	88314000	0	36479000	0	29438000	12159666.67	0.616296534	0.413060217	-1.275575978	
O43795	MYO1B		873430000	1063300000	1208600000	1253500000	1360200000	1692000000	1048443333	1435233333	0.077612725	1.368918365	0.453036414	
O00159	MYO1C		4283400000	3713300000	3341600000	2482500000	2523100000	3032600000	3779433333	2679400000	0.02795518	0.708942258	-0.496259968	
O94832	MYO1D		106140000	99867000	86156000	126080000	121350000	0	97387666.67	82476666.67	0.73861382	0.846890263	-0.239753052	
Q9NZM1	MYOF		509430000	433140000	446230000	460920000	485560000	578240000	462933333.3	508240000	0.34931366	1.097868664	0.134705477	
Q13765	NACA		0	0	0	240720000	231280000	209090000	0	227030000	1.72483E-05	N/A	N/A	
P17050	NAGA		0	0	56971000	0	34116000	0	18990333.33	11372000	0.748045657	0.598830984	-0.739779225	
P43490	NAMPT		134390000	135160000	100830000	138880000	131770000	193350000	123460000	154666666.7	0.237790847	1.252767428	0.325118608	
P55209	NAP1L1		611060000	656470000	513240000	691660000	766190000	1215400000	593590000	891083333.3	0.153067217	1.501176457	0.58609357	
Q99733	NAP1L4		0	0	0	41951000	27203000	24889000	0	31347666.67	0.004215885	N/A	N/A	
P54920	NAPA		177590000	235290000	271740000	242550000	309150000	208460000	228206666.7	253386666.7	0.56612306	1.110338582	0.150999673	
Q99747	NAPG		163270000	122060000	0	125460000	151840000	0	95110000	92433333.33	0.970400892	0.971857148	-0.041183826	
Q69YL0	NCBP2AS2		215000000	212130000	241100000	345680000	209460000	360010000	222743333.3	305050000	0.167298849	1.369513491	0.453663478	
Q6PIU2	NCEH1		153100000	181480000	196600000	94688000	11466000	124560000	177060000	76904666.67	0.050339062	0.434342407	-1.203095276	
Q9Y2A7	NCKAP1		124370000	0	40338000	0	35948000	119780000	54902666.67	51909333.33	0.956014648	0.945479272	-0.080882264	
P19338	NCL		2742300000	2778300000	2917100000	2539400000	2567100000	2765700000	2812566667	2624066667	0.101538687	0.932979366	-0.10008292	
Q969V3	NCLN		367360000	327960000	310250000	434020000	390860000	467530000	335190000	430803333.3	0.026549536	1.285251151	0.362050304	
P62166	NCS1		218420000	43646000	93573000	0	0	0	118546333.3	0	0.08470911	0	N/A	
Q9UMX5	NENF		411400000	382170000	411590000	238920000	418990000	494660000	401720000	384190000	0.829939323	0.956362641	-0.064370321	
P48681	NES		962160000	1066400000	950320000	450660000	501070000	445120000	992960000	465616666.7	0.000209651	0.468917848	-1.092592902	
Q99519	NEU1		70710000	89745000	0	353860000	154030000	311290000	53485000	273060000	0.030049712	5.105356642	2.352011746	
Q0ZGT2	NEXN		77395000	77088000	48616000	69361000	79033000	49662000	67699666.67	66018666.67	0.902414991	0.975169745	-0.036274729	

P35240	NF2		475590000	225910000	375310000	268180000	228060000	276010000	358936666.7	257416666.7	0.242245304	0.717164588	-0.479623842	
Q6ZNB6	NFXL1		38626000	33264000	48004000	85700000	53489000	0	39964666.67	46396333.33	0.81230145	1.160933825	0.215285739	
Q8NEJ9	NGDN		0	0	133490000	0	183320000	0	44496666.67	61106666.67	0.836835344	1.373286388	0.45763252	
Q5JS37	NHLRC3		0	0	0	51268000	54461000	0	0	35243000	0.116479157	N/A	N/A	
Q96TA1	NIBAN2		105650000	163980000	103160000	106440000	0	95921000	124263333.3	67453666.67	0.221473749	0.542828402	-0.881431887	
P14543	NID1		0	0	0	117600000	72857000	107380000	0	99279000	0.001839673	N/A	N/A	
Q9BPW8	NIPSNAP1		797830000	1055300000	806820000	1242400000	1172700000	1490000000	886650000	1301700000	0.031604166	1.468110303	0.553960366	
O75323	NIPSNAP2		367470000	258500000	312490000	0	253550000	137940000	312820000	130496666.7	0.084221141	0.417162159	-1.261319798	
P15531	NME1		0	0	0	42771000	34191000	75765000	0	50909000	0.015899833	N/A	N/A	
P22392	NME2		1182400000	1362300000	1673000000	1046700000	1072900000	743550000	1405900000	954383333.3	0.064262217	0.678841549	-0.558853227	
Q13232	NME3		65943000	0	103040000	0	0	0	56327666.67	0	0.134921769	0	N/A	
P30419	NMT1		0	0	0	0	154550000	165930000	0	106826666.7	0.116617808	N/A	N/A	
P40261	NNMT		24758000	68996000	18439000	114240000	155730000	99060000	37397666.67	123010000	0.021113456	3.289242644	1.717755438	
Q8NC60	NOA1		475690000	537590000	429960000	119420000	45413000	120800000	481080000	95211000	0.000640213	0.19791095	-2.337076656	
Q14978	NOLC1		54078000	24397000	34467000	0	36060000	0	37647333.33	12020000	0.159401097	0.319278922	-1.647110782	
Q5JPE7	NOMO2		366980000	439310000	468930000	500520000	455940000	466430000	425073333.3	474296666.7	0.21158129	1.115799627	0.158077974	
Q15233	NONO		269780000	151050000	338960000	499830000	504070000	213370000	253263333.3	405756666.7	0.240573678	1.602113742	0.679976575	
P46087	NOP2		0	40229000	0	0	0	55871000	13409666.67	18623666.67	0.831408969	1.388823983	0.473863766	
Q8TAT6	NPLOC4		88367000	66780000	126370000	64942000	114030000	145830000	93839000	108267333.3	0.647928883	1.153756256	0.206338471	
P06748	NPM1		526980000	365710000	515730000	482280000	431520000	599690000	469473333.3	504496666.7	0.652059082	1.074601326	0.103801524	
O14786	NRP1		0	53020000	0	109230000	130160000	83153000	17673333.33	107514333.3	0.015746269	6.08342135	2.604882932	
Q15738	NSDHL		244680000	301690000	247420000	222510000	314320000	398740000	264596666.7	311856666.7	0.432196826	1.178611472	0.237088213	
P46459	NSF		752020000	867040000	806370000	876900000	851250000	1135500000	808476666.7	954550000	0.205283137	1.180677241	0.239614632	
Q08J23	NSUN2		0	0	0	121540000	83713000	0	0	68417666.67	0.129447345	N/A	N/A	
Q96CB9	NSUN4		0	0	71117000	74800000	101680000	0	23705666.67	58826666.67	0.413979499	2.481544497	1.311238324	
Q8TCD5	NT5C		46508000	0	15531000	0	0	0	20679666.67	0	0.204891442	0	N/A	
Q9BSD7	NTPCR		258760000	74341000	114270000	293940000	259020000	408060000	149123666.7	320340000	0.075759238	2.148149969	1.103094716	
Q02818	NUCB1		281470000	151140000	146750000	91814000	142570000	0	193120000	78128000	0.131436858	0.404556752	-1.305585993	
Q9Y266	NUDC		0	0	0	369740000	194230000	0	0	187990000	0.153122041	N/A	N/A	
O43809	NUDT21		0	0	0	63808000	54485000	67050000	0	61781000	8.08412E-05	N/A	N/A	
P49757	NUMB		148840000	177310000	163740000	217090000	203190000	102300000	163296666.7	174193333.3	0.783546402	1.066729266	0.093194069	
P35658	NUP214		0	21887000	0	48255000	88379000	76579000	7295666.667	71071000	0.010281264	9.741536072	3.284149278	
Q9NX40	OCIAD1		707970000	864310000	834470000	800640000	719780000	432430000	802250000	650950000	0.281233559	0.811405422	-0.301505151	
Q56VL3	OCIAD2		310550000	277370000	226100000	737640000	666920000	410020000	271340000	604860000	0.031282684	2.229158989	1.156499517	
Q16625	OCLN		0	0	136180000	0	0	64697000	45393333.33	21565666.67	0.660130069	0.475084447	-1.073744117	
Q5SWX8	ODR4		0	34467000	0	71730000	64967000	120720000	11489000	85805666.67	0.023999376	7.468506107	2.900819696	
Q9NTK5	OLA1		0	0	0	0	75815000	56267000	0	44027333.33	0.124755306	N/A	N/A	
Q9H6K4	OPA3		0	72989000	0	0	65389000	0	24329666.67	21796333.33	0.941906827	0.895874721	-0.158631095	
Q13438	OS9		0	0	0	0	47172000	68698000	0	38623333.33	0.12965968	N/A	N/A	
P22059	OSBP		0	0	0	74921000	67743000	105620000	0	82761333.33	0.002051308	N/A	N/A	
Q9BXB5	OSBPL10		127650000	0	0	54478000	82087000	0	42550000	45521666.67	0.954465588	1.069839405	0.097394248	
Q9BZF1	OSBPL8		62298000	78348000	247760000	81710000	113530000	84808000	129468666.7	93349333.33	0.580760305	0.721018728	-0.471891362	
Q96SU4	OSBPL9		0	0	70612000	58427000	43411000	60253000	23537333.33	54030333.33	0.275046797	2.295516343	1.198818703	
Q32P28	P3H1		463290000	424930000	239220000	362350000	296090000	618330000	375813333.3	425590000	0.699955487	1.132450507	0.179448	
Q8IVL5	P3H2		0	103710000	180140000	0	0	0	94616666.67	0	0.144116301	0	N/A	
Q8IVL6	P3H3		1069600000	916320000	1060800000	866870000	896510000	879080000	1015573333	880820000	0.055686212	0.867313045	-0.205375286	
Q92791	P3H4		0	0	73289000	86816000	96453000	77947000	24429666.67	87072000	0.066413475	3.564191079	1.833574683	
P13674	P4HA1		1317400000	1378000000	1668900000	2018000000	2401600000	2688300000	1454766667	2369300000	0.014711881	1.62864606	0.703673109	
O15460	P4HA2		1842400000	1955300000	2244300000	2952800000	3237900000	3209900000	2014000000	3133533333	0.001729963	1.555875538	0.637726657	
P07237	P4HB		11222000000	13184000000	12684000000	24548000000	23774000000	19267000000	12363333333	22529666667	0.004356418	1.822297115	0.865758201	
Q9UQ80	PA2G4		548220000	623530000	616640000	1440400000	1245600000	891640000	596130000	1192546667	0.02134712	2.000480879	1.00034684	
P11940	PABPC1		2855200000	2804700000	2248400000	3303000000	3108700000	3932200000	2636100000	3447966667	0.061778943	1.307980223	0.387340727	
Q13310	PABPC4		275420000	305270000	252950000	235670000	269560000	218800000	277880000	241343333.3	0.160963974	0.868516386	-0.203375027	
Q86U42	PABPN1		0	0	0	0	504100000	489080000	0	331060000	0.116207484	N/A	N/A	
Q9UNF0	PACSIN2		186270000	147120000	199560000	0	0	0	177650000	0	0.000351145	0	N/A	
Q9UKS6	PACSIN3		772380000	778700000	741270000	289770000	449510000	379420000	764116666.7	372900000	0.001199716	0.488014483	-1.035004131	
Q15102	PAFAH1B3		0	74575000	0	0	108780000	137740000	24858333.33	82173333.33	0.304862405	3.305665437	1.724940719	

P22234	PAICS		1021700000	1249100000	1376400000	1896500000	1810700000	1831000000	1215733333	1846066667	0.004141392	1.51847993	0.60262784	
Q13177	PAK2		0	0	71449000	97206000	94526000	66721000	23816333.33	86151000	0.072578845	3.61730745	1.854916224	
Q8WX93	PALLD		189110000	150310000	122480000	0	88233000	71317000	153966666.7	53183333.33	0.03867217	0.345421087	-1.533571938	
Q8IXS6	PALM2		19996000	20436000	0	0	0	0	13477333.33	0	0.116163626	0	N/A	
A6NDB9	PALM3		124420000	74273000	67248000	0	65780000	0	88647000	21926666.67	0.078356988	0.247348096	-2.015385301	
O95340	PAPSS2		0	14728000	0	76159000	62243000	153300000	4909333.333	97234000	0.032520348	19.80594785	4.307861841	
Q8TEW0	PARD3		252540000	236390000	0	75453000	94682000	129150000	162976666.7	99761666.67	0.489298446	0.61212239	-0.708107956	
Q99497	PARK7		76024000	78954000	84367000	76568000	69842000	0	79781666.67	48803333.33	0.276410704	0.611711128	-0.709077573	
P09874	PARP1		881140000	653700000	752070000	703170000	771710000	588620000	762303333.3	687833333.3	0.429376165	0.902309229	-0.148306152	
Q460N5	PARP14		0	0	24540000	0	23835000	38646000	8180000	20827000	0.414832338	2.54608802	1.348282295	
Q8NI35	PATJ		71791000	73821000	0	0	0	0	48537333.33	0	0.116193822	0	N/A	
Q96AQ6	PBXIP1		128230000	206870000	47568000	122830000	261900000	265990000	127556000	216906666.7	0.246005123	1.70048188	0.765943633	
Q15365	PCBP1		2226400000	2398400000	2802400000	2788200000	2990700000	3624900000	2475733333	3134600000	0.096403744	1.266129901	0.340425428	
Q15366	PCBP2		1069900000	1007900000	1711500000	1231900000	1659500000	2164200000	1263100000	1685200000	0.295430696	1.334177816	0.415950959	
Q9Y5E4	PCDHB5		0	0	94717000	0	68650000	86974000	31572333.33	51874666.67	0.648005691	1.643041904	0.716369275	
P12004	PCNA		73782000	79410000	65008000	0	109070000	78190000	72733333.33	62420000	0.768419827	0.858203483	-0.220608339	
Q9UHG3	PCYOX1		82089000	122830000	0	0	0	0	68306333.33	0	0.131595561	0	N/A	
Q13442	PDAP1		0	0	0	111690000	116510000	166270000	0	131490000	0.001659588	N/A	N/A	
Q9BUL8	PDCD10		109230000	0	133890000	173810000	184390000	0	81040000	119400000	0.625043725	1.473346496	0.559096757	
O14737	PDCD5		0	0	59457000	0	96770000	0	19819000	32256666.67	0.758991687	1.627562776	0.70271319	
O75340	PDCD6		0	0	0	42930000	230890000	278680000	0	184166666.7	0.062669151	N/A	N/A	
Q8WUM4	PDCD6IP		538200000	406950000	435380000	962920000	810060000	646100000	460176666.7	806360000	0.025600989	1.752283543	0.809236241	
P30101	PDIA3		21597000000	18101000000	24087000000	27458000000	25500000000	25220000000	21261666667	26059333333	0.062578154	1.225648663	0.293545485	
P13667	PDIA4		2305400000	3081900000	3348300000	3087400000	3029200000	1868500000	2911866667	2661700000	0.646571177	0.914087183	-0.129596322	
Q14554	PDIA5		205920000	156590000	120360000	237750000	283640000	278990000	160956666.7	266793333.3	0.021218664	1.65754758	0.729050284	
Q15084	PDIA6		19962000000	17826000000	17259000000	22397000000	21143000000	19699000000	18349000000	21079666667	0.073630958	1.148818283	0.200150614	
Q96JY6	PDLIM2		0	100710000	96821000	125080000	179330000	191040000	65843666.67	165150000	0.062251773	2.508213901	1.326660386	
P50479	PDLIM4		303480000	258070000	274000000	925530000	672740000	260910000	278516666.7	619726666.7	0.153652883	2.225097241	1.153868386	
Q9NR12	PDLIM7		487530000	564530000	676450000	248610000	225380000	359100000	576170000	277696666.7	0.012164755	0.48197002	-1.052984684	
Q15121	PEA15		0	0	75077000	104670000	0	150450000	25025666.67	85040000	0.305174642	3.398111272	1.764733095	
Q8IZL8	PELP1		0	0	0	0	117430000	162720000	0	93383333.33	0.126406793	N/A	N/A	
O96011	PEX11B		0	0	0	241280000	248420000	238290000	0	242663333.3	1.40944E-07	N/A	N/A	
O75381	PEX14		160080000	0	163470000	379140000	308510000	375440000	107850000	354363333.3	0.013633341	3.285705455	1.716203157	
Q9UHV9	PFDN2		139860000	0	99894000	186480000	0	0	79918000	62160000	0.823984758	0.777797242	-0.362533975	
P17858	PFKL		0	55150000	0	0	0	95021000	18383333.33	31673666.67	0.73503902	1.722955576	0.784885504	
P18669	PGAM1		354400000	338780000	434540000	684410000	738980000	574130000	375906666.7	665840000	0.006977813	1.771290746	0.824801041	
P52209	PGD		950410000	662840000	880310000	863240000	1125600000	1117400000	831186666.7	1035413333	0.169749614	1.245704936	0.316962385	
P00558	PGK1		1540500000	1324300000	1414100000	933200000	1008400000	1157700000	1426300000	1033100000	0.012448908	0.724321671	-0.465297554	
O00264	PGRMC1		1386600000	1515300000	1589100000	1198400000	1126700000	1044600000	1497000000	1123233333	0.007224451	0.750322868	-0.414416566	
O15173	PGRMC2		429480000	453370000	427490000	261750000	330510000	419810000	436780000	337356666.7	0.099324199	0.772372056	-0.372632125	
P35232	PHB		9313400000	8992100000	7043800000	10256000000	10447000000	15956000000	8449766667	12219666667	0.132372349	1.446154332	0.532221523	
Q99623	PHB2		2655500000	2478100000	2679400000	4452500000	4410300000	4083000000	2604333333	4315266667	0.000210017	1.656956355	0.728535602	
O43175	PHGDH		2512100000	2725500000	2727400000	2053400000	2083700000	1854800000	2655000000	1997300000	0.002900566	0.752278719	-0.410660815	
Q9BTU6	PI4K2A		488860000	427240000	410630000	513100000	479560000	528890000	442243333.3	507183333.3	0.080384234	1.146842236	0.197666943	
P42356	PI4KA		0	43195000	0	0	58792000	0	14398333.33	19597333.33	0.841164871	1.361083459	0.444755533	
Q13492	PICALM		321680000	442760000	381540000	287460000	295840000	213770000	381993333.3	265690000	0.055973961	0.695535699	-0.523803531	
Q92643	PIGK		105340000	0	0	53294000	69722000	0	35113333.33	41005333.33	0.892515637	1.167799506	0.223792606	
Q9H490	PIGU		0	218450000	0	72612000	46901000	77803000	72816666.67	65772000	0.92819521	0.903254749	-0.146795159	
P48739	PITPNB		0	0	0	129980000	133890000	182620000	0	148830000	0.000924087	N/A	N/A	
P14618	PKM		2048700000	2270000000	2177600000	5148200000	4849500000	5335300000	2165433333	5111000000	4.55631E-05	2.360266613	1.238949834	
Q16513	PKN2		257240000	229770000	207760000	165060000	261180000	172940000	231590000	199726666.7	0.401393372	0.8624149	-0.213545991	
Q99959	PKP2		111070000	96622000	340280000	0	0	203440000	182657333.3	67813333.33	0.331655377	0.371259845	-1.42949881	
Q01970	PLCB3		410620000	347230000	386010000	88918000	161050000	120520000	381286666.7	123496000	0.000758552	0.323892784	-1.626411768	
P51178	PLCD1		30217000	28785000	0	0	33922000	0	19667333.33	11307333.33	0.606803716	0.574929663	-0.798542627	
Q8N3E9	PLCD3		201080000	199230000	233900000	184860000	369340000	292160000	211403333.3	282120000	0.265424052	1.334510651	0.41631082	
Q8IV08	PLD3		0	204720000	172550000	315250000	374520000	402190000	125756666.7	363986666.7	0.025447822	2.89437273	1.533250721	
Q15149	PLEC		49503000000	50244000000	48949000000	51986000000	53915000000	50177000000	49565333333	52026000000	0.097588051	1.049644913	0.069901358	

Q9HAU0	PLEKHA5		0	0	179380000	0	77221000	73729000	59793333.33	50316666.67	0.890933063	0.841509644	-0.248948289	
Q99541	PLIN2		107210000	131140000	166330000	248060000	222790000	190020000	134893333.3	220290000	0.023694214	1.633068103	0.707584956	
O60664	PLIN3		762970000	678110000	933440000	1994500000	2164200000	2050900000	791506666.7	2069866667	0.000143564	2.615096946	1.386864431	
Q02809	PLOD1		2306200000	2998600000	2894600000	1428100000	1688500000	1953700000	2733133333	1690100000	0.016723817	0.618374515	-0.693447232	
O00469	PLOD2		648400000	563820000	610160000	1408000000	1515400000	2034100000	607460000	1652500000	0.005832654	2.720343726	1.443788953	
O60568	PLOD3		1119300000	817570000	994980000	812070000	862810000	1011500000	977283333.3	895460000	0.483415752	0.916274707	-0.126147899	
O94903	PLPBP		0	67272000	0	0	90646000	0	22424000	30215333.33	0.846071693	1.347455108	0.430237208	
Q14651	PLS1		71779000	68166000	126750000	64543000	88894000	0	88898333.33	51145666.67	0.311255811	0.575327621	-0.797544359	
P13797	PLS3		175870000	97346000	195490000	280310000	147900000	167360000	156235333.3	198523333.3	0.453769909	1.270668607	0.345587821	
Q9UIW2	PLXNA1		39499000	27247000	0	0	0	0	22248666.67	0	0.129342971	0	N/A	
O15031	PLXNB2		594680000	427840000	490050000	792110000	598200000	454320000	504190000	614876666.7	0.368509572	1.219533641	0.286329556	
Q8IY17	PNPLA6		61104000	0	0	30741000	6218900	66374000	20368000	34444633.33	0.627549643	1.691115148	0.757974896	
O00592	PODXL		275960000	0	169340000	468410000	288210000	269600000	148433333.3	342073333.3	0.131442308	2.304558724	1.204490531	
Q9H488	POFUT1		213180000	189390000	128630000	163170000	195150000	0	177066666.7	119440000	0.428411962	0.674548193	-0.568006575	
Q9Y2G5	POFUT2		0	0	0	62812000	50474000	0	0	37762000	0.120813096	N/A	N/A	
Q7Z4H8	POGLUT3		0	0	102110000	90100000	72630000	81067000	34036666.67	81265666.67	0.241806512	2.387591813	1.255556212	
Q9Y2S7	POLDIP2		285190000	354770000	359530000	358930000	346980000	219280000	333163333.3	308396666.7	0.651023297	0.925662088	-0.11144246	
Q15165	PON2		372270000	335230000	457370000	521160000	422420000	459200000	388290000	467593333.3	0.161430925	1.204237383	0.268119808	
P16435	POR		897100000	818220000	512920000	391420000	538380000	802260000	742746666.7	577353333.3	0.380196229	0.777322012	-0.363415724	
Q6S8J3	POTEE		439420000	0	359530000	393650000	498430000	728650000	266316666.7	540243333.3	0.177283981	2.028575005	1.020466646	
Q15181	PPA1		0	0	0	95460000	113770000	0	0	69743333.33	0.119153739	N/A	N/A	
Q86W92	PPFIBP1		96522000	118260000	130470000	171420000	81146000	0	115084000	84188666.67	0.573696149	0.731541019	-0.450989333	
P62937	PPIA		4855800000	3878300000	3694200000	3586000000	3930000000	4341500000	4142766667	3952500000	0.67505475	0.95407256	-0.067829103	
P23284	PPIB		3495600000	3771700000	3296800000	2102200000	2247900000	2256500000	3521366667	2202200000	0.000842501	0.625382191	-0.67718996	
P45877	PPIC		281360000	213110000	100610000	203880000	244340000	212660000	198360000	220293333.3	0.705967567	1.110573368	0.151304705	
Q9UNP9	PPIE		278530000	293260000	182290000	282690000	0	179960000	251360000	154216666.7	0.339465164	0.613529069	-0.704796396	
P50336	PPOX		60358000	0	40174000	23597000	0	0	33510666.67	7865666.667	0.256834396	0.234721283	-2.090979434	
P62136	PPP1CA		201870000	712410000	110790000	158030000	196840000	842010000	341690000	398960000	0.853206572	1.167608066	0.223556082	
P36873	PPP1CC		281560000	223060000	244330000	0	0	0	249650000	0	0.000127914	0	N/A	
O14974	PPP1R12A		181580000	81714000	94745000	80310000	25329000	67519000	119346333.3	57719333.33	0.157334023	0.483628878	-1.048027702	
P67775	PPP2CA		168200000	149610000	138160000	181840000	183570000	183710000	151990000	183040000	0.024035362	1.204289756	0.268182551	
P30153	PPP2R1A		697280000	520400000	783700000	678330000	580500000	772740000	667126666.7	677190000	0.920996847	1.015084592	0.021599959	
Q16537	PPP2R5E		42633000	0	0	68637000	0	0	14211000	22879000	0.763696363	1.609950039	0.687015918	
P50897	PPT1		1902800000	1629700000	1760800000	1715500000	1556000000	1859600000	1764433333	1710366667	0.670409163	0.96935749	-0.044899279	
O60831	PRAF2		647070000	265400000	343410000	341240000	252260000	436500000	418626666.7	343333333.3	0.587981654	0.820142052	-0.286054283	
P42785	PRCP		79112000	247390000	0	179690000	74056000	160400000	108834000	138048666.7	0.732985901	1.268433271	0.343047625	
Q06830	PRDX1		2553000000	2075800000	2273900000	5330400000	5323100000	5743300000	2300900000	5465600000	8.61818E-05	2.375418315	1.248181596	
P32119	PRDX2		0	0	35138000	68950000	63428000	0	11712666.67	44126000	0.265017174	3.767374353	1.913559397	
Q13162	PRDX4		151370000	207470000	263130000	269860000	257110000	196010000	207323333.3	240993333.3	0.442024624	1.162403331	0.217110743	
P30041	PRDX6		0	0	0	0	216350000	190790000	0	135713333.3	0.117681819	N/A	N/A	
Q9HCU5	PREB		576710000	514190000	467380000	549530000	620040000	554190000	519426666.7	574586666.7	0.230192214	1.106194009	0.145604433	
P49643	PRIM2		26101000	35760000	0	0	0	0	20620333.33	0	0.125722427	0	N/A	
P17612	PRKACA		0	0	51706000	0	0	33072000	17235333.33	11024000	0.776573625	0.639616292	-0.644721408	
P10644	PRKAR1A		0	0	0	40740000	46999000	0	0	29246333.33	0.118136737	N/A	N/A	
P13861	PRKAR2A		248920000	199080000	355140000	167630000	152810000	324820000	267713333.3	215086666.7	0.50388034	0.80342157	-0.315770899	
P14314	PRKCSH		2110300000	2254000000	2299800000	2822500000	2081600000	1171300000	2221366667	2025133333	0.704129075	0.911660989	-0.133430652	
P78527	PRKDC		599350000	497130000	546310000	205880000	272640000	84368000	547596666.7	187629333.3	0.004512606	0.342641482	-1.545228273	
O75569	PRKRA		195580000	109440000	152330000	197100000	104540000	253600000	152450000	185080000	0.550077727	1.214037389	0.279812854	
Q9UNN8	PROCR		88635000	91797000	102770000	90262000	91835000	94133000	94400666.67	92076666.67	0.627443309	0.97538153	-0.035961442	
Q9UMS4	PRPF19		234390000	246990000	324770000	295520000	357380000	287270000	268716666.7	313390000	0.281191619	1.166246976	0.221873341	
Q6P2Q9	PRPF8		0	82592000	54608000	47052000	0	0	45733333.33	15684000	0.356887483	0.342944606	-1.543952529	
P11908	PRPS2		108460000	0	0	118330000	137340000	0	36153333.33	85223333.33	0.431504375	2.357274571	1.237119811	
O60256	PRPSAP2		0	85796000	22864000	44475000	54213000	54564000	36220000	51084000	0.596269669	1.410381005	0.49608495	
Q96HE9	PRR11		0	149570000	159490000	0	0	0	103020000	0	0.11652614	0	N/A	
Q5THK1	PRR14L		0	0	1791000000	1034200000	959050000	709340000	597000000	900863333.3	0.641889535	1.508983808	0.593577325	
P07602	PSAP		439620000	363560000	289670000	548880000	342680000	559310000	364283333.3	483623333.3	0.222759192	1.327602141	0.408822861	
P28066	PSMA5		0	0	0	147020000	152520000	50681000	0	116740333.3	0.024223315	N/A	N/A	

P35998	PSMC2	0	0	0	0	165930000	158580000	0	108170000	0.116320529	N/A	N/A
P17980	PSMC3	141360000	86047000	47138000	70897000	91870000	104560000	91515000	89109000	0.93796155	0.973709228	-0.03843708
P43686	PSMC4	127270000	80201000	63180000	227730000	114870000	62979000	90217000	135193000	0.438086133	1.498531319	0.583549236
P62333	PSMC6	0	156610000	139140000	223540000	275920000	194880000	98583333.33	231446666.7	0.0728821	2.34772612	1.231264117
Q99460	PSMD1	0	0	0	0	38793000	23934000	0	20909000	0.137939526	N/A	N/A
O00231	PSMD11	296680000	327900000	224100000	213230000	361670000	425250000	282893333.3	333383333.3	0.510293374	1.178477165	0.236923804
Q13200	PSMD2	0	130030000	137970000	291060000	264090000	211270000	89333333.33	255473333.3	0.030203925	2.859776119	1.515902209
O43242	PSMD3	0	0	0	88303000	103210000	0	0	63837666.67	0.118520866	N/A	N/A
P51665	PSMD7	7407900	6125100	0	0	0	49357000	4511000	16452333.33	0.511955069	3.647158797	1.866773016
Q06323	PSME1	117970000	148530000	0	143310000	119290000	69666000	88833333.33	110755333.3	0.684918193	1.246776735	0.31820314
Q9UL46	PSME2	295660000	317290000	204710000	98173000	110230000	344430000	272553333.3	184277666.7	0.368923459	0.67611599	-0.564657329
P26599	PTBP1	298080000	324040000	356760000	244800000	272400000	462520000	326293333.3	326573333.3	0.997021831	1.000858124	0.00123748
Q9H7Z7	PTGES2	543450000	373680000	443370000	308750000	352760000	373900000	453500000	345136666.7	0.109759628	0.761051084	-0.3939348
Q9P2B2	PTGFRN	159920000	154550000	0	0	310580000	159480000	104823333.3	156686666.7	0.643785012	1.494768976	0.579922526
Q13308	PTK7	1134700000	1067600000	978250000	954900000	1007400000	1198800000	1060183333	1053700000	0.944091382	0.993884705	-0.008849592
P18031	PTPN1	215860000	281150000	206710000	326410000	366400000	321700000	234573333.3	338170000	0.019405734	1.44163872	0.527709665
P10586	PTPRF	499230000	449620000	414720000	306640000	317230000	449610000	454523333.3	357826666.7	0.137153338	0.78725698	-0.345093452
P53801	PTTG1IP	0	0	0	127130000	136550000	183410000	0	149030000	0.001021269	N/A	N/A
Q9Y606	PUS1	61648000	108650000	78758000	0	103030000	0	83018666.67	34343333.33	0.25853032	0.413682063	-1.273405689
Q92626	PXDN	26915000	24012000	26424000	33209000	33208000	0	25783666.67	22139000	0.759238039	0.858644361	-0.219867384
Q96C36	PYCR2	981110000	848540000	679020000	555900000	528650000	351020000	836223333.3	478523333.3	0.030011044	0.57224346	-0.805299026
P47897	QARS1	899070000	679860000	522150000	917730000	580850000	1095500000	700360000	864693333.3	0.427619352	1.234641232	0.304091878
P09417	QDPR	48831000	58417000	0	78705000	66492000	0	35749333.33	48399000	0.698846663	1.353843428	0.437060901
Q6ZRP7	QSOX2	96393000	417010000	423240000	101770000	175790000	411370000	312214333.3	229643333.3	0.5938318	0.73553104	-0.443141869
P61026	RAB10	352350000	435690000	192960000	279290000	270370000	241090000	327000000	263583333.3	0.428974625	0.80606524	-0.311031486
Q15907	RAB11B	2381200000	2541600000	3093300000	1966000000	2186500000	1667700000	2672033333	1940066667	0.049596386	0.726063797	-0.461831777
Q6WKZ4	RAB11FIP1	0	0	154150000	0	0	96118000	51383333.33	32039333.33	0.765372395	0.623535517	-0.681456355
Q9BXF6	RAB11FIP5	0	0	0	50617000	58925000	0	0	36514000	0.118399465	N/A	N/A
P51153	RAB13	0	0	16349000	0	68290000	43344000	5449666.667	37211333.33	0.199401284	6.82818521	2.771502191
P61106	RAB14	621850000	480100000	469310000	287130000	288390000	386970000	523753333.3	320830000	0.026644568	0.612559347	-0.70707847
Q9NP72	RAB18	362980000	304070000	447800000	399810000	476410000	610550000	371616666.7	495590000	0.170912003	1.333605418	0.41533187
P62820	RAB1A	2047200000	1838400000	2191200000	1787900000	2222100000	1766700000	2025600000	1925566667	0.60858805	0.950615456	-0.073066237
Q9H0U4	RAB1B	356050000	408850000	360980000	198470000	353710000	516840000	375293333.3	356340000	0.849167623	0.949497282	-0.074764224
Q9UL25	RAB21	100330000	206880000	161460000	124630000	128500000	156960000	156223333.3	136696666.7	0.580444769	0.875008001	-0.192631885
Q9ULC3	RAB23	102700000	133990000	132460000	0	61347000	0	123050000	20449000	0.010895709	0.166184478	-2.589142459
P61019	RAB2A	1385700000	1391800000	1407300000	1399100000	1831400000	2113100000	1394933333	1781200000	0.136493786	1.276906901	0.352653342
Q13636	RAB31	0	0	79364000	0	0	82932000	26454666.67	27644000	0.976692217	1.044957411	0.063444145
Q9BZG1	RAB34	237780000	277140000	345980000	176960000	201460000	185700000	286966666.7	188040000	0.037980073	0.655267743	-0.609843581
Q15286	RAB35	114640000	79841000	58985000	154720000	158550000	89084000	84488666.67	134118000	0.148559255	1.587408173	0.666673139
P51148	RAB5C	140600000	0	0	106010000	149290000	0	46866666.67	85100000	0.585349271	1.815789474	0.860596943
P51149	RAB7A	1672300000	1260000000	1737600000	1415900000	1417300000	1977700000	1556633333	1603633333	0.85395553	1.030193366	0.042915155
P61006	RAB8A	70916000	137280000	172820000	209820000	171210000	145960000	127005333.3	175663333.3	0.238650996	1.383117769	0.467924004
Q92930	RAB8B	68297000	45425000	62307000	185630000	130370000	69495000	58676333.33	128498333.3	0.110972039	2.189951656	1.130899022
Q9UI14	RABAC1	0	0	0	105410000	134440000	125410000	0	121753333.3	0.000143024	N/A	N/A
Q5HYI8	RABL3	0	0	0	39182000	44060000	0	0	27747333.33	0.117480617	N/A	N/A
P63000	RAC1	904900000	1270100000	1937400000	2768400000	1481000000	999250000	1370800000	1749550000	0.567360488	1.276298512	0.351965799
Q9H0H5	RACGAP1	109130000	166220000	0	141210000	122910000	103490000	91783333.33	122536666.7	0.571502549	1.335064463	0.416909404
P63244	RACK1	471170000	323500000	325730000	749370000	807470000	836520000	373466666.7	797786666.7	0.001536874	2.136165655	1.095023529
Q9P0K7	RAI14	83664000	88241000	76961000	140600000	99326000	0	82955333.33	79975333.33	0.946655601	0.964077054	-0.052779637
P11233	RALA	892870000	753700000	878650000	515880000	696060000	677570000	841740000	629836666.7	0.042803837	0.748255598	-0.418396929
P11234	RALB	0	0	145400000	268600000	0	265460000	48466666.67	178020000	0.270302508	3.67303989	1.876974564
Q9UKM9	RALY	87632000	88061000	91666000	126550000	119040000	124340000	89119666.67	123310000	0.000184353	1.383645211	0.46847406
P62826	RAN	91476000	80033000	90325000	293130000	199460000	130370000	87278000	207653333.3	0.063657945	2.379217367	1.250487083
P43487	RANBP1	0	0	0	367790000	474590000	441930000	0	428103333.3	0.000171744	N/A	N/A
P46060	RANGAP1	0	0	84583000	165540000	103720000	209140000	28194333.33	159466666.7	0.034320154	5.655982881	2.499777753
P61224	RAP1B	1224600000	1369100000	785270000	1234600000	1515600000	693920000	1126323333	1148040000	0.945450814	1.019281024	0.027551868
P61225	RAP2B	38160000	212510000	99499000	98488000	176120000	207430000	116723000	160679333.3	0.507503771	1.376586734	0.461095512

P54136	RARS1		486350000	633490000	750260000	546010000	570320000	717120000	623366666.7	611150000	0.902040688	0.980402118	-0.028554495	
Q96PK6	RBM14		51324000	89768000	0	0	0	0	47030666.67	0	0.144766897	0	N/A	
P98179	RBM3		331280000	281750000	169440000	98947000	201290000	238470000	260823333.3	179569000	0.269871153	0.688469845	-0.538534628	
Q14498	RBM39		204710000	208790000	180200000	0	0	152520000	197900000	50840000	0.046442381	0.256897423	-1.960735677	
P38159	RBMX		307610000	310960000	233360000	214930000	234260000	277070000	283976666.7	242086666.7	0.251562322	0.85248788	-0.230248771	
P18754	RCC1		59736000	56072000	39603000	0	0	0	51803666.67	0	0.001115798	0	N/A	
Q96I51	RCC1L		133900000	107770000	113450000	108300000	88931000	75964000	118373333.3	91065000	0.09054961	0.769303334	-0.378375534	
Q9P258	RCC2		0	52376000	71158000	93002000	63483000	50440000	41178000	68975000	0.32397729	1.675044927	0.744199791	
Q15293	RCN1		2192800000	2095500000	2710700000	3901600000	4382700000	3227000000	2333000000	3837100000	0.01755277	1.644706387	0.717830057	
Q14257	RCN2		1265700000	1486000000	856500000	734620000	857600000	2034700000	1202733333	1208973333	0.989682376	1.005188182	0.007465615	
Q96D15	RCN3		583660000	711630000	653750000	941930000	1342200000	1338000000	649680000	1207376667	0.015508047	1.858417477	0.894074627	
Q8TC12	RDH11		319270000	423380000	478940000	445130000	394200000	358300000	407196666.7	399210000	0.887820392	0.980386218	-0.028577892	
Q8NBN7	RDH13		37089000	0	13627000	0	0	0	16905333.33	0	0.193599109	0	N/A	
Q9HBH5	RDH14		128380000	202100000	109240000	202640000	285310000	204080000	146573333.3	230676666.7	0.099331844	1.573796962	0.654249428	
P35241	RDX		341900000	346510000	327880000	280460000	265410000	319310000	338763333.3	288393333.3	0.041479263	0.851312125	-0.232239916	
P46063	RECQL		0	0	224940000	0	0	142630000	74980000	47543333.33	0.7727249	0.634080199	-0.657262769	
Q00765	REEP5		119090000	131260000	164130000	546850000	319020000	191850000	138160000	352573333.3	0.109982365	2.551920479	1.351583373	
Q8IUW5	RELL1		13994000	29487000	28066000	59784000	36638000	7607700	23849000	34676566.67	0.532853562	1.45400506	0.54003229	
Q96D71	REPS1		0	0	0	44024000	68833000	0	0	37619000	0.134971476	N/A	N/A	
Q86VR2	RETREG3		0	0	0	95190000	85187000	0	0	60125666.67	0.117338331	N/A	N/A	
Q6NUM9	RETSAT		0	0	0	104490000	0	70599000	0	58363000	0.130801148	N/A	N/A	
P35250	RFC2		49912000	62316000	0	59614000	63630000	0	37409333.33	41081333.33	0.902114427	1.098157323	0.13508475	
P35249	RFC4		68686000	0	0	56586000	0	87126000	22895333.33	47904000	0.506180414	2.092304109	1.065092557	
Q14699	RFTN1		135150000	76258000	90844000	372440000	275140000	276810000	100750666.7	308130000	0.004837327	3.058342046	1.612749767	
Q96CC6	RHBDF1		0	7850300	0	0	6374000	0	2616766.667	2124666.667	0.890988715	0.811943493	-0.300548769	
P61586	RHOA		1416200000	1073600000	785490000	1249800000	1647700000	1999200000	1091763333	1632233333	0.128782512	1.495043187	0.58018716	
P08134	RHOC		0	0	0	0	250410000	93836000	0	114748666.7	0.19126538	N/A	N/A	
P84095	RHOG		119830000	229940000	365040000	259650000	255020000	159340000	238270000	224670000	0.870187196	0.942921895	-0.084789821	
P17081	RHOQ		0	0	32978000	0	0	39029000	10992666.67	13009666.67	0.911440614	1.18348596	0.243042592	
Q96DB5	RMDN1		432860000	396490000	452450000	221100000	247870000	438780000	427266666.7	302583333.3	0.151557053	0.708183804	-0.497804245	
Q96TC7	RMDN3		455530000	85778000	467630000	97973000	164720000	195590000	336312666.7	152761000	0.22661709	0.454223153	-1.138526847	
Q9NWS8	RMND1		0	68440000	0	0	92846000	38696000	22813333.33	43847333.33	0.58325226	1.922004676	0.942611846	
O00584	RNASET2		115120000	0	0	0	75453000	0	38373333.33	25151000	0.787520295	0.655429117	-0.609488329	
P13489	RNH1		113600000	128890000	206900000	274210000	292210000	322180000	149796666.7	296200000	0.010332335	1.97734707	0.98356612	
Q13464	ROCK1		0	0	0	96127000	0	74061000	0	56729333.33	0.122760012	N/A	N/A	
O75116	ROCK2		303240000	361630000	370930000	114780000	77838000	230810000	345266666.7	141142666.7	0.015805363	0.408793203	-1.290556885	
P35244	RPA3		0	0	0	42385000	41522000	39435000	0	41114000	1.23104E-06	N/A	N/A	
P27635	RPL10		528080000	803060000	731070000	1382700000	1141400000	597430000	687403333.3	1040510000	0.225116797	1.513681924	0.598062078	
P62906	RPL10A		2152700000	1967400000	1918900000	2535600000	2561400000	1665000000	2013000000	2254000000	0.471039481	1.119721808	0.163140343	
P62913	RPL11		0	0	1088200000	0	0	1148800000	362733333.3	382933333.3	0.971286182	1.055688293	0.078183921	
P30050	RPL12		2298600000	2149200000	2694700000	2604100000	2785100000	3014700000	2380833333	2801300000	0.105227025	1.17660483	0.234629864	
P26373	RPL13		931180000	1064400000	1105000000	2064800000	1629700000	703180000	1033526667	1465893333	0.345783035	1.418341085	0.504204516	
P40429	RPL13A		418770000	757470000	707490000	1683400000	1052700000	378870000	627910000	1038323333	0.353292761	1.653618088	0.725626074	
P50914	RPL14		817260000	618510000	617370000	1020900000	1185000000	971480000	684380000	1059126667	0.015527452	1.547571038	0.630005634	
P61313	RPL15		273370000	385270000	606510000	1535500000	658640000	385700000	421716666.7	859946666.7	0.290842204	2.039157412	1.027973148	
P18621	RPL17		317230000	284870000	392910000	776190000	586340000	497680000	331670000	620070000	0.030757328	1.869539	0.902682567	
Q07020	RPL18		1349200000	1766600000	1815100000	4795600000	3274100000	1506300000	1643633333	3192000000	0.182736591	1.942038979	0.957572157	
Q02543	RPL18A		162370000	210760000	322520000	539740000	641560000	299530000	231883333.3	493610000	0.07953581	2.128699777	1.089972493	
P84098	RPL19		598010000	1238100000	955340000	1921000000	1711000000	990640000	930483333.3	1540880000	0.144460987	1.65599957	0.727702298	
P46778	RPL21		794170000	670660000	523140000	1025400000	1287900000	492820000	662656666.7	935373333.3	0.330912661	1.411550476	0.49728072	
P35268	RPL22		1622600000	1845200000	1861500000	3083900000	2863700000	1707800000	1776433333	2551800000	0.148304207	1.436473833	0.522531713	
P62829	RPL23		1174500000	1137300000	1461700000	1295300000	1484200000	1995600000	1257833333	1591700000	0.225077492	1.265429972	0.339627672	
P62750	RPL23A		327790000	478000000	386970000	835500000	632990000	522870000	397586666.7	663786666.7	0.058542283	1.669539555	0.739450275	
P83731	RPL24		423300000	445000000	401540000	608070000	715310000	621140000	423280000	648173333.3	0.003358956	1.531311031	0.614767345	
P61254	RPL26		334860000	438540000	430170000	1052600000	604970000	314920000	401190000	657496666.7	0.303219869	1.63886604	0.712697934	
P61353	RPL27		1772700000	2120100000	2358800000	3446300000	3250300000	2356800000	2083866667	3017800000	0.067935513	1.448173268	0.534234225	
P46776	RPL27A		905480000	1141400000	1375400000	2368100000	1743800000	1575400000	1140760000	1895766667	0.052496115	1.661845319	0.732786106	

P46779	RPL28		365320000	457080000	237000000	395430000	342450000	225140000	353133333.3	321006666.7	0.712793223	0.909023976	-0.137609748	
P47914	RPL29		0	0	10184000	40161000	22320000	9094700	3394666.667	23858566.67	0.100517906	7.028250196	2.81316555	
P39023	RPL3		521010000	442630000	501330000	949270000	764360000	550070000	488323333.3	754566666.7	0.086523059	1.545219356	0.627811655	
P62888	RPL30		707320000	644660000	754480000	2742100000	1937800000	1880200000	702153333.3	2186700000	0.006079909	3.114277034	1.638897286	
P62899	RPL31		47898000	140560000	103290000	227390000	209030000	201640000	97249333.33	212686666.7	0.014555881	2.187024418	1.128969328	
P62910	RPL32		72762000	125210000	148820000	512410000	406480000	182240000	115597333.3	367043333.3	0.065552894	3.175188586	1.666842281	
P49207	RPL34		0	0	38297000	0	63540000	42144000	12765666.67	35228000	0.376777969	2.759589524	1.464453689	
P42766	RPL35		317540000	401400000	561170000	1616400000	940430000	299710000	426703333.3	952180000	0.245861953	2.231480107	1.158000946	
P18077	RPL35A		58849000	81223000	102700000	128290000	132030000	113120000	80924000	124480000	0.035198272	1.538233404	0.621274428	
Q9Y3U8	RPL36		374090000	296380000	320340000	300180000	237520000	208710000	330270000	248803333.3	0.083135536	0.753333131	-0.408640115	
P63173	RPL38		0	0	0	295320000	384720000	307100000	0	329046666.7	0.000301796	N/A	N/A	
Q59GN2	RPL39P5		0	0	0	58159000	44699000	0	0	34286000	0.122883076	N/A	N/A	
P36578	RPL4		5849500000	5846400000	6540800000	8745000000	8039000000	7589100000	6078900000	8124366667	0.007422887	1.336486316	0.418445066	
P46777	RPL5		478090000	632250000	996630000	1270800000	1181900000	1401300000	702323333.3	1284666667	0.024910999	1.829167003	0.871186799	
Q02878	RPL6		1594100000	1863100000	2017000000	4196100000	3159000000	2356700000	1824733333	3237266667	0.061049757	1.774103979	0.827090567	
P18124	RPL7		973940000	1731700000	1985300000	4860800000	3738400000	2177300000	1563646667	3592166667	0.072100341	2.297300754	1.199939741	
P62424	RPL7A		1700900000	2193900000	2091200000	2701600000	2742100000	1831200000	1995333333	2424966667	0.266405878	1.215319078	0.281335138	
P62917	RPL8		822370000	1059700000	1555600000	2735900000	2117900000	1094300000	1145890000	1982700000	0.186288114	1.730270794	0.790997843	
P32969	RPL9		405060000	420320000	460480000	612100000	636050000	603130000	428620000	617093333.3	0.000607205	1.439721276	0.525789539	
P05388	RPLP0		3578700000	3823900000	3822500000	3884100000	4528000000	5064400000	3741700000	4492166667	0.099160972	1.200568369	0.263717563	
P05386	RPLP1		9227800000	5554400000	4671400000	6519900000	6427500000	9807700000	6484533333	7585033333	0.570668307	1.169711519	0.226152768	
P05387	RPLP2		7884800000	8322200000	6567300000	5912400000	7028700000	7632300000	7591433333	6857800000	0.371391236	0.903360367	-0.146626475	
P04843	RPN1		6128900000	5853500000	6391300000	4156200000	4173800000	3899300000	6124566667	4076433333	0.000331618	0.665587225	-0.587300351	
P04844	RPN2		3571000000	1993100000	3487900000	2948100000	2558400000	3824600000	3017333333	3110366667	0.890580074	1.030832965	0.043810579	
P46783	RPS10		375210000	362980000	251160000	452490000	333780000	369890000	329783333.3	385386666.7	0.352045557	1.16860565	0.22478817	
P62280	RPS11		90920000	99071000	88034000	121190000	115360000	40254000	92675000	92268000	0.988380903	0.995608309	-0.006349825	
P25398	RPS12		1310500000	1520800000	1021100000	2028000000	2022700000	1980400000	1284133333	2010366667	0.007561134	1.565543557	0.646663648	
P62277	RPS13		575270000	450530000	558100000	783170000	769240000	743120000	527966666.7	765176666.7	0.004341187	1.449289728	0.535346033	
P62263	RPS14		77483000	0	120790000	537570000	373520000	271870000	66091000	394320000	0.018183627	5.966319166	2.576841156	
P62841	RPS15		47688000	0	0	0	118840000	123330000	15896000	80723333.33	0.209536792	5.078216742	2.344321972	
P62244	RPS15A		126360000	164110000	0	135510000	140520000	101370000	96823333.33	125800000	0.601152891	1.299273591	0.377705254	
P62249	RPS16		0	245550000	287870000	404780000	471810000	587260000	177806666.7	487950000	0.041080063	2.744272806	1.456423906	
P08708	RPS17		285630000	231160000	119620000	541400000	443480000	400330000	212136666.7	461736666.7	0.017773646	2.176599991	1.122076297	
P62269	RPS18		238720000	216990000	75957000	689810000	418210000	327830000	177222333.3	478616666.7	0.066154282	2.700656614	1.433310214	
P39019	RPS19		436830000	578170000	495860000	974720000	852340000	1086800000	503620000	971286666.7	0.0041057	1.928610196	0.94756158	
P15880	RPS2		174580000	271590000	0	321290000	443840000	458460000	148723333.3	407863333.3	0.04589371	2.742430015	1.455454805	
P60866	RPS20		526960000	621090000	429880000	867410000	771340000	741770000	525976666.7	793506666.7	0.016202336	1.50863473	0.593243544	
P63220	RPS21		238800000	227320000	195110000	359440000	280140000	445350000	220410000	361643333.3	0.046150761	1.640775524	0.714377876	
P62266	RPS23		0	0	0	58739000	139430000	205720000	0	134629666.7	0.033924828	N/A	N/A	
P62847	RPS24		102060000	103740000	56740000	0	75080000	206590000	87513333.33	93890000	0.923408979	1.072865087	0.101468669	
P62851	RPS25		369320000	407500000	488520000	721830000	666700000	833940000	421780000	740823333.3	0.006184733	1.756421199	0.812638852	
P62854	RPS26		0	80219000	0	406710000	350540000	98147000	26739666.67	285132333.3	0.058735273	10.66327179	3.414578261	
P42677	RPS27		0	111190000	0	0	0	52563000	37063333.33	17521000	0.658457028	0.472731361	-1.080907519	
P62979	RPS27A		0	0	53790000	137380000	137420000	85823000	17930000	120207666.7	0.014644179	6.704275888	2.745081519	
P62857	RPS28		429690000	433750000	478800000	339770000	433200000	500640000	447413333.3	424536666.7	0.666284871	0.948869055	-0.075719088	
P23396	RPS3		326840000	316330000	435200000	646400000	655100000	581120000	359456666.7	627540000	0.003854521	1.745801534	0.80388956	
P61247	RPS3A		772240000	1033600000	865860000	1400400000	1348200000	813290000	890566666.7	1187296667	0.216864115	1.333192349	0.414884944	
P62701	RPS4X		297180000	415920000	435040000	1149000000	672740000	446330000	382713333.3	756023333.3	0.152338287	1.975429826	0.982166598	
P46782	RPS5		1610400000	1758300000	1010500000	1671100000	2076900000	2164200000	1459733333	1970733333	0.136163846	1.350063939	0.433027734	
P62753	RPS6		0	315680000	293250000	518920000	490520000	435760000	202976666.7	481733333.3	0.056071894	2.373343406	1.246920863	
P62081	RPS7		941800000	987330000	681960000	1210900000	1017900000	1207600000	870363333.3	1145466667	0.074184815	1.316078726	0.396245791	
P62241	RPS8		2052400000	1823100000	1525600000	2206300000	2138200000	2515600000	1800366667	2286700000	0.064169967	1.270130159	0.344976347	
P46781	RPS9		119610000	100100000	97328000	220540000	231130000	197710000	105679333.3	216460000	0.000789881	2.048271816	1.034407181	
P08865	RPSA		1264100000	1231200000	1256100000	1295800000	1507500000	1835200000	1250466667	1546166667	0.13315636	1.236471717	0.30622924	
P10301	RRAS		35104000	106650000	76647000	164740000	147350000	38145000	72800333.33	116745000	0.381417756	1.603632767	0.681343802	
Q9P2E9	RRBP1		3140800000	3229400000	3355600000	4019300000	3683500000	2083200000	3241933333	3262000000	0.974945919	1.006189722	0.008902358	
O76021	RSL1D1		40299000	59362000	89118000	81221000	82022000	75444000	62926333.33	79562333.33	0.31098132	1.264372626	0.338421706	

Q96DX4	RSPRY1		80417000	0	0	0	71947000	58333000	26805666.67	43426666.67	0.657130835	1.620055461	0.696043203	
Q15404	RSU1		0	0	162420000	0	39539000	0	54140000	13179666.67	0.503053687	0.243436769	-2.038381005	
Q9Y3I0	RTCB		351170000	193800000	188880000	295530000	311180000	341790000	244616666.7	316166666.7	0.263167189	1.292498467	0.370162569	
Q9NQC3	RTN4		253080000	197670000	877880000	1080800000	1231300000	2085100000	442876666.7	1465733333	0.055063621	3.309574524	1.726645757	
Q9Y224	RTRAF		254340000	249670000	241440000	250320000	200740000	286130000	248483333.3	245730000	0.917745935	0.988919445	-0.016075088	
Q96GQ5	RUSF1		114430000	85732000	127690000	72695000	110680000	201450000	109284000	128275000	0.660885989	1.173776582	0.23115783	
Q9Y265	RUVBL1		127530000	200810000	201700000	288310000	370550000	402690000	176680000	353850000	0.013494982	2.002773376	1.001999182	
Q9Y230	RUVBL2		122170000	134330000	192720000	417200000	479860000	408760000	149740000	435273333.3	0.000796987	2.906860781	1.539461978	
P60903	S100A10		540140000	752390000	937700000	1905900000	1383500000	1027200000	743410000	1438866667	0.067821131	1.935495442	0.95270291	
P31949	S100A11		111780000	0	24452000	126920000	253640000	160740000	45410666.67	180433333.3	0.056680177	3.973368959	1.990362765	
Q96FQ6	S100A16		116020000	0	0	31953000	56486000	51195000	38673333.33	46544666.67	0.851342772	1.203533873	0.267276747	
Q9NTJ5	SACM1L		208390000	220410000	0	126490000	158770000	0	142933333.3	95086666.67	0.609267085	0.665251866	-0.588027444	
Q9Y512	SAMM50		138470000	209530000	169570000	241030000	371450000	50037000	172523333.3	220839000	0.639759284	1.28005294	0.356203478	
Q9NR31	SAR1A		0	0	109560000	183190000	232550000	0	36520000	138580000	0.26910632	3.794633078	1.923960391	
P49591	SARS1		377990000	360240000	297660000	736840000	583270000	707280000	345296666.7	675796666.7	0.003364274	1.95714796	0.968752827	
Q9Y3A5	SBDS		0	0	0	0	175690000	206500000	0	127396666.7	0.118695006	N/A	N/A	
O15126	SCAMP1		782880000	791620000	731130000	840260000	766620000	1198300000	768543333.3	935060000	0.283874961	1.216665293	0.282932335	
O15127	SCAMP2		232570000	151610000	158230000	265570000	277980000	237620000	180803333.3	260390000	0.049513752	1.440183625	0.526252769	
O14828	SCAMP3		495870000	501330000	543970000	567060000	729520000	618300000	513723333.3	638293333.3	0.068481848	1.242484606	0.313227977	
Q969E2	SCAMP4		0	0	150290000	0	103920000	216540000	50096666.67	106820000	0.518031861	2.132277597	1.092395272	
Q14108	SCARB2		0	54349000	137660000	674030000	531130000	342140000	64003000	515766666.7	0.012263339	8.058476426	3.010507102	
Q8NBX0	SCCPDH		452610000	489910000	569800000	955640000	764930000	607220000	504106666.7	775930000	0.063143202	1.53921789	0.622197473	
Q8WVM8	SCFD1		1177600000	891400000	1057400000	1081800000	1091600000	1102900000	1042133333	1092100000	0.580477465	1.04794652	0.067565093	
Q8WU76	SCFD2		72786000	63244000	47519000	41121000	45405000	0	61183000	28842000	0.117267894	0.471405456	-1.084959639	
P22307	SCP2		484370000	566010000	579610000	245180000	210690000	186810000	543330000	214226666.7	0.000654101	0.394284628	-1.342690632	
Q9HB40	SCPEP1		0	0	0	91183000	49172000	106950000	0	82435000	0.008770636	N/A	N/A	
Q14160	SCRIB		1564500000	1485000000	1497200000	1339800000	1466500000	1332600000	1515566667	1379633333	0.05311338	0.910308575	-0.135572423	
P18827	SDC1		90971000	0	148330000	0	0	0	79767000	0	0.138447756	0	N/A	
O00560	SDCBP		0	0	105880000	75935000	138090000	159830000	35293333.33	124618333.3	0.108269842	3.530931243	1.820048728	
Q99470	SDF2		77302000	0	0	101010000	123190000	180140000	25767333.33	134780000	0.035462291	5.230653799	2.386991286	
Q9HCN8	SDF2L1		532330000	506730000	462460000	328540000	425720000	501180000	500506666.7	418480000	0.203212709	0.836112739	-0.258230611	
Q9BRK5	SDF4		145980000	194440000	200310000	236720000	204310000	189870000	180243333.3	210300000	0.245358976	1.166756052	0.222502951	
Q9NRG7	SDR39U1		0	0	10416000	24534000	31533000	0	3472000	18689000	0.208969852	5.382776498	2.428350523	
Q96IW7	SEC22A		0	33827000	0	39981000	47349000	96754000	11275666.67	61361333.33	0.076424136	5.441925089	2.444117097	
O75396	SEC22B		1093200000	1105800000	1135700000	930450000	817930000	1076600000	1111566667	941660000	0.088864798	0.84714667	-0.239316324	
Q15436	SEC23A		825860000	876830000	704100000	526970000	759420000	963110000	802263333.3	749833333.3	0.719509008	0.934647393	-0.097505901	
Q15437	SEC23B		106640000	107140000	72274000	61099000	63497000	128490000	95351333.33	84362000	0.681881961	0.884749033	-0.176659814	
O95487	SEC24B		32041000	0	41933000	0	0	29075000	24658000	9691666.667	0.400935376	0.393043502	-1.347239097	
P53992	SEC24C		679990000	588420000	654570000	655980000	642260000	654550000	640993333.3	650930000	0.737366723	1.015501981	0.022193054	
O94979	SEC31A		0	0	0	150870000	166060000	176720000	0	164550000	2.55443E-05	N/A	N/A	
P61619	SEC61A1		58069000	35448000	209510000	279580000	358460000	394110000	101009000	344050000	0.019410626	3.406132127	1.7681344	
P60059	SEC61G		723220000	583910000	589130000	154010000	155350000	311640000	632086666.7	207000000	0.003598787	0.327486737	-1.610491615	
Q9UGP8	SEC63		0	101160000	87150000	238960000	181860000	235140000	62770000	218653333.3	0.013087183	3.483405024	1.800498228	
Q9UBV2	SEL1L		0	137800000	0	37733000	38402000	106370000	45933333.33	60835000	0.785751604	1.324419448	0.405360101	
O60613	SELENOF		55800000	74169000	250130000	212650000	160990000	284880000	126699666.7	219506666.7	0.264683841	1.732496008	0.792852028	
Q9C0D9	SELENOI		0	0	0	110200000	191840000	0	0	100680000	0.144324838	N/A	N/A	
O75326	SEMA7A		0	0	0	125490000	184260000	242700000	0	184150000	0.005533929	N/A	N/A	
Q9P0V9	SEPTIN10		304880000	260860000	258580000	119110000	154730000	451700000	274773333.3	241846666.7	0.772627867	0.880167896	-0.184149344	
Q9NVA2	SEPTIN11		742550000	1126100000	1023400000	2330400000	1502300000	831360000	964016666.7	1554686667	0.258157595	1.612717622	0.689493853	
Q15019	SEPTIN2		2686800000	3321000000	3392800000	3914000000	3494700000	4105200000	3133533333	3837966667	0.070621494	1.2248048	0.292551842	
Q99719	SEPTIN5		206870000	150500000	111730000	109440000	109450000	0	156366666.7	72963333.33	0.142430571	0.466616926	-1.099689454	
Q14141	SEPTIN6		55510000	74057000	0	0	155060000	0	43189000	51686666.67	0.887276275	1.196755347	0.259128251	
Q16181	SEPTIN7		2548100000	2408800000	3155000000	3476900000	3198300000	3705300000	2703966667	3460166667	0.049793368	1.279663211	0.355764163	
Q92599	SEPTIN8		213890000	196110000	195130000	653500000	495590000	279150000	201710000	476080000	0.06503328	2.360220118	1.238921414	
Q9UHD8	SEPTIN9		1348800000	1438600000	1380900000	1859900000	1894900000	1284400000	1389433333	1679733333	0.21963053	1.208934098	0.273735602	
Q8NC51	SERBP1		218090000	236220000	107050000	239550000	400170000	289220000	187120000	309646666.7	0.120730923	1.654802622	0.726659149	
O75635	SERPINB7		0	0	59618000	40020000	0	0	19872666.67	13340000	0.798414695	0.671273776	-0.575026811	

P50454	SERPINH1	18070000000	13389000000	14795000000	20970000000	22130000000	22297000000	15418000000	21799000000	0.011634003	1.413866909	0.499646322
O75533	SF3B1	78986000	72093000	91511000	132980000	96486000	0	80863333.33	76488666.67	0.91832886	0.945900491	-0.080239676
P31947	SFN	159650000	33708000	51715000	192910000	135850000	47037000	81691000	125265666.7	0.493279092	1.533408413	0.616742
P23246	SFPQ	581990000	756530000	715420000	564270000	509040000	593660000	684646666.7	555656666.7	0.091097928	0.811596249	-0.301165897
Q9H9B4	SFXN1	2145300000	1840400000	1836200000	1237200000	1334300000	1107100000	1940633333	1226200000	0.004202515	0.63185558	-0.662333249
Q96NB2	SFXN2	99541000	0	0	0	0	78160000	33180333.33	26053333.33	0.87404291	0.785204087	-0.348860413
Q9BWM7	SFXN3	861790000	1011100000	1091500000	853980000	962710000	871560000	988130000	896083333.3	0.28846014	0.906847615	-0.141067952
Q6P4A7	SFXN4	105810000	81163000	0	0	62391000	0	62324333.33	20797000	0.337377973	0.333689891	-1.583420113
O95470	SGPL1	351380000	600790000	429210000	488780000	570620000	418710000	460460000	492703333.3	0.726029081	1.070024179	0.097643397
Q9P0V3	SH3BP4	115220000	130060000	115530000	0	24740000	0	120270000	8246666.667	0.000307145	0.068567944	-3.866321919
Q9Y371	SH3GLB1	0	0	0	153200000	143570000	0	0	98923333.33	0.116535171	N/A	N/A
P29353	SHC1	125080000	137820000	0	168260000	173780000	187620000	87633333.33	176553333.3	0.11543807	2.014682389	1.010552418
Q96FS4	SIPA1	124580000	58211000	93485000	0	0	0	92092000	0	0.008625912	0	N/A
P63208	SKP1	0	0	0	46303000	19008000	0	0	21770333.33	0.180526857	N/A	N/A
P55011	SLC12A2	1355900000	1186200000	1146600000	1039400000	942070000	836680000	1229566667	939383333.3	0.028828525	0.763995446	-0.388364057
Q9Y666	SLC12A7	0	59249000	0	95324000	0	0	19749666.67	31774666.67	0.763987587	1.608871036	0.686048687
Q9BXP2	SLC12A9	46571000	0	66589000	163560000	208020000	303960000	37720000	225180000	0.015030788	5.969777306	2.577677115
P53985	SLC16A1	178700000	253870000	262850000	369630000	149810000	74045000	231806666.7	197828333.3	0.732170304	0.853419516	-0.228672992
P43007	SLC1A4	127950000	139640000	156900000	111050000	0	143670000	141496666.7	84906666.67	0.270471574	0.60006125	-0.736818327
Q15758	SLC1A5	554220000	1418900000	1965300000	730810000	958390000	1143400000	1312806667	944200000	0.437439963	0.719222429	-0.475490082
P12235	SLC25A4	229440000	106510000	237940000	37185000	32722000	76091000	191296666.7	48666000	0.033058441	0.254400669	-1.97482563
P05141	SLC25A5	4874000000	5326300000	4951700000	8754300000	8448000000	8343800000	5050666667	8515366667	4.911E-05	1.685988648	0.753594823
P12236	SLC25A6	1065200000	702070000	679990000	461190000	717340000	1244300000	815753333.3	807610000	0.976709694	0.990017407	-0.014474203
P11166	SLC2A1	603420000	505910000	614500000	1109600000	1183000000	605560000	574610000	966053333.3	0.101465925	1.681233068	0.749519738
P11169	SLC2A3	0	0	73365000	0	178640000	101570000	24455000	93403333.33	0.294624908	3.81939617	1.933344572
Q8NEW0	SLC30A7	0	0	0	0	154670000	104580000	0	86416666.67	0.13074842	N/A	N/A
Q6PML9	SLC30A9	0	0	77068000	0	128490000	0	25689333.33	42830000	0.748726048	1.667228941	0.737452226
L0R6Q1	SLC35A4	0	163010000	259130000	149690000	178290000	115710000	140713333.3	147896666.7	0.930842188	1.051049415	0.071830499
Q8TB61	SLC35B2	92080000	125830000	101930000	93370000	55950000	109220000	106613333.3	86180000	0.335994913	0.808341671	-0.306962873
Q96K37	SLC35E1	52985000	52893000	58289000	202450000	130820000	0	54722333.33	111090000	0.395628998	2.030066944	1.021527303
Q9HBR0	SLC38A10	52628000	0	52041000	0	0	0	34889666.67	0	0.116129033	0	N/A
Q96QD8	SLC38A2	0	90288000	119030000	0	0	0	69772666.67	0	0.123561769	0	N/A
Q8NBW4	SLC38A9	0	0	0	120290000	125220000	0	0	81836666.67	0.116276884	N/A	N/A
Q9ULF5	SLC39A10	75112000	72712000	0	68408000	46508000	0	49274666.67	38305333.33	0.747867921	0.777383916	-0.363300836
Q15043	SLC39A14	1153700000	957490000	1133000000	352150000	476520000	476660000	1081396667	435110000	0.000986637	0.402359295	-1.313443735
P08195	SLC3A2	5692700000	5796900000	6180700000	8641500000	8452000000	7203500000	5890100000	8099000000	0.009649093	1.3750191	0.459451659
Q9Y6M7	SLC4A7	68315000	24340000	0	0	0	0	30885000	0	0.197238976	0	N/A
P53794	SLC5A3	81998000	63834000	69269000	0	33648000	0	71700333.33	11216000	0.008268433	0.15642884	-2.676421572
Q9Y289	SLC5A6	306730000	307880000	205610000	67935000	69112000	0	273406666.7	45682333.33	0.005087574	0.16708566	-2.581340175
Q01650	SLC7A5	52149000	46242000	36862000	123240000	92439000	35039000	45084333.33	83572666.67	0.21611305	1.853696406	0.890404982
O14745	SLC9A3R1	374270000	338930000	470180000	516550000	458630000	478590000	394460000	484590000	0.102583368	1.228489581	0.296885622
Q15599	SLC9A3R2	276390000	291120000	242930000	574390000	308100000	90959000	270146666.7	324483000	0.718682412	1.20113642	0.264400015
Q9NWH9	SLTM	0	0	81572000	88623000	0	81304000	27190666.67	56642333.33	0.495462624	2.083153533	1.058769173
O95347	SMC2	308880000	285640000	210480000	290830000	211030000	216370000	268333333.3	239410000	0.502664553	0.89221118	-0.164542869
Q9NTJ3	SMC4	179160000	135730000	101590000	96484000	106530000	84063000	138826666.7	95692333.33	0.13864578	0.689293604	-0.536809467
O00161	SNAP23	243790000	313780000	371150000	233880000	158610000	0	309573333.3	130830000	0.084117235	0.42261392	-1.242587807
O95295	SNAPIN	111640000	0	146330000	149090000	91549000	118830000	85990000	119823000	0.512870764	1.393452727	0.47866406
Q7KZF4	SND1	2784100000	2646100000	2415500000	2848600000	3453300000	3979100000	2615233333	3427000000	0.077596313	1.310399327	0.390006521
O75643	SNRNP200	100530000	109460000	78565000	0	0	0	96185000	0	0.000468878	0	N/A
P08621	SNRNP70	175060000	98020000	0	122450000	152950000	47898000	91026666.67	107766000	0.792390725	1.183894829	0.243540926
P62314	SNRPD1	0	0	0	172620000	195900000	0	0	122840000	0.117701409	N/A	N/A
P63162	SNRPN	23959000	26321000	28513000	27413000	31735000	32725000	26264333.33	30624333.33	0.105881666	1.166004594	0.221573473
Q13425	SNTB2	173550000	104050000	184830000	0	218150000	0	154143333.3	72716666.67	0.349802201	0.471747075	-1.083914522
Q9UMY4	SNX12	0	0	0	55002000	67848000	0	0	40950000	0.120447494	N/A	N/A
O60749	SNX2	0	199480000	172360000	208480000	134070000	177570000	123946666.7	173373333.3	0.496106205	1.398773666	0.484162541
O60493	SNX3	0	0	36365000	0	0	70764000	12121666.67	23588000	0.687768543	1.945937027	0.960465024
Q9Y5X3	SNX5	164500000	154100000	179970000	0	190010000	257020000	166190000	149010000	0.835091471	0.896624346	-0.157424422

Q9UNH7	SNX6		101040000	157080000	97726000	150150000	120360000	232170000	118615333.3	167560000	0.273361641	1.412633555	0.498387271	
Q9Y5X2	SNX8		0	51250000	0	58789000	0	0	17083333.33	19596333.33	0.927642707	1.147102439	0.197994233	
Q9Y5X1	SNX9		0	0	74446000	70295000	49022000	0	24815333.33	39772333.33	0.668217091	1.602732182	0.68053337	
P35610	SOAT1		0	0	112430000	0	88244000	117260000	37476666.67	68501333.33	0.579071231	1.827839545	0.87013943	
Q99523	SORT1		0	0	0	73852000	70103000	61620000	0	68525000	4.57796E-05	N/A	N/A	
Q07617	SPAG1		0	0	89470000	77424000	0	0	29823333.33	25808000	0.923807224	0.865362691	-0.208623172	
P09486	SPARC		892110000	1040200000	862010000	2637900000	3071300000	2984500000	931440000	2897900000	0.000163772	3.111204157	1.637473068	
Q8TB22	SPATA20		148110000	117900000	118100000	164170000	0	0	128036666.7	54723333.33	0.257997774	0.427403608	-1.226329007	
Q15005	SPCS2		59191000	95970000	55331000	79737000	49633000	15100000	70164000	48156666.67	0.387678426	0.686344374	-0.542995463	
Q9H2V7	SPNS1		0	0	0	210990000	194890000	0	0	135293333.3	0.116741994	N/A	N/A	
P35270	SPR		329700000	368430000	298940000	253550000	390540000	315750000	332356666.7	319946666.7	0.793768968	0.962660596	-0.054900857	
Q5W111	SPRYD7		51595000	52489000	40390000	0	0	0	48158000	0	0.000245323	0	N/A	
Q13813	SPTAN1		7088000000	7392500000	7295600000	4873700000	4833200000	4027000000	7258700000	4577966667	0.000761258	0.630686854	-0.665004233	
Q01082	SPTBN1		9029100000	9145600000	8674100000	5918500000	5729100000	5410500000	8949600000	5686033333	9.12178E-05	0.635339382	-0.654400646	
O15269	SPTLC1		321820000	179550000	230620000	301300000	297260000	310390000	243996666.7	302983333.3	0.230953604	1.241751937	0.312376997	
O15270	SPTLC2		104650000	0	288140000	83135000	59197000	76052000	130930000	72794666.67	0.5293199	0.555981568	-0.84689104	
Q14534	SQLE		0	0	0	84479000	96360000	0	0	60279666.67	0.117830568	N/A	N/A	
Q13501	SQSTM1		192940000	146800000	163690000	98729000	103500000	120830000	167810000	107686333.3	0.016226295	0.641715829	-0.639993524	
O75044	SRGAP2		50863000	33632000	0	0	0	0	28165000	0	0.132388336	0	N/A	
P37108	SRP14		0	182060000	0	485780000	422010000	383510000	60686666.67	430433333.3	0.005440867	7.092716687	2.826338321	
P61011	SRP54		0	0	0	198520000	0	142420000	0	113646666.7	0.126772903	N/A	N/A	
Q9UHB9	SRP68		208450000	175670000	120040000	197570000	247830000	379330000	168053333.3	274910000	0.149616748	1.63584973	0.710040228	
O76094	SRP72		150920000	124780000	259520000	368320000	387650000	337290000	178406666.7	364420000	0.013173299	2.042636673	1.030432612	
P49458	SRP9		606780000	661310000	454450000	486040000	540330000	692040000	574180000	572803333.3	0.98818155	0.997602378	-0.003463191	
P08240	SRPRA		559700000	550980000	759500000	805320000	921760000	1127900000	623393333.3	951660000	0.047737538	1.526580329	0.610303507	
Q9Y5M8	SRPRB		249080000	221490000	348780000	1261600000	1168100000	782810000	273116666.7	1070836667	0.006236485	3.920803076	1.971149184	
Q07955	SRSF1		36370000	66230000	72995000	106530000	76171000	99807000	58531666.67	94169333.33	0.070341859	1.6088613	0.686039957	
P84103	SRSF3		267280000	260360000	261880000	400150000	237090000	151110000	263173333.3	262783333.3	0.9959965	0.998518087	-0.002139534	
P43307	SSR1		325750000	396340000	309600000	305180000	494080000	596990000	343896666.7	465416666.7	0.246096462	1.35336196	0.436547743	
Q9UNL2	SSR3		701640000	586170000	540360000	481460000	778090000	754360000	609390000	671303333.3	0.592461773	1.101598867	0.13959898	
P51571	SSR4		1008000000	1059300000	959990000	1490000000	1676200000	1814200000	1009096667	1660133333	0.002686614	1.64516779	0.718234732	
Q08945	SSRP1		167230000	212410000	0	0	0	172260000	126546666.7	57420000	0.468645584	0.453745654	-1.140044271	
P50502	ST13		175590000	0	0	316670000	273680000	288490000	58530000	292946666.7	0.017316885	5.005068626	2.32338985	
Q14849	STARD3		0	0	0	34325000	38953000	40722000	0	38000000	3.74361E-05	N/A	N/A	
P42224	STAT1		0	99974000	152050000	87354000	108410000	0	84008000	65254666.67	0.752871655	0.776767292	-0.364445642	
O95793	STAU1		127130000	121710000	171450000	163700000	134710000	141350000	140096666.7	146586666.7	0.737087059	1.046325156	0.065331254	
Q658P3	STEAP3		225230000	249160000	0	476550000	457660000	0	158130000	311403333.3	0.430189786	1.969286874	0.97767329	
Q13586	STIM1		33436000	28037000	35974000	39746000	37045000	0	32482333.33	25597000	0.625270526	0.788028364	-0.343680536	
P31948	STIP1		185000000	73268000	171310000	357100000	214660000	167960000	143192666.7	246573333.3	0.197090369	1.721969002	0.784059172	
Q9Y6E0	STK24		0	0	0	127420000	214980000	174490000	0	172296666.7	0.002429696	N/A	N/A	
P16949	STMN1		0	0	0	107110000	124750000	149990000	0	127283333.3	0.000514748	N/A	N/A	
Q9Y3F4	STRAP		91422000	0	0	261940000	347080000	269360000	30474000	292793333.3	0.003027455	9.607971823	3.26423192	
P46977	STT3A		512470000	380820000	316120000	526410000	551010000	573620000	403136666.7	550346666.7	0.068202164	1.365161525	0.44907166	
Q8TCJ2	STT3B		139480000	181690000	176850000	172560000	146900000	188000000	166006666.7	169153333.3	0.869225271	1.018955062	0.027090427	
O60499	STX10		58673000	54092000	0	0	28156000	0	37588333.33	9385333.333	0.251336513	0.249687403	-2.001805057	
Q86Y82	STX12		230130000	262100000	0	284900000	257310000	272880000	164076666.7	271696666.7	0.264215452	1.655912886	0.727626778	
O14662	STX16		71070000	114090000	80334000	81487000	64981000	188100000	88498000	111522666.7	0.602142404	1.260171605	0.333620207	
Q9P2W9	STX18		30438000	0	0	19236000	52726000	36525000	10146000	36162333.33	0.136997045	3.564196071	1.833576703	
Q13277	STX3		69072000	177510000	95077000	84502000	87281000	0	113886333.3	57261000	0.262534626	0.502790794	-0.991969859	
Q12846	STX4		62820000	66715000	87234000	190790000	149430000	152090000	72256333.33	164103333.3	0.003932809	2.271127329	1.183408592	
Q13190	STX5		90400000	0	0	195440000	296700000	0	30133333.33	164046666.7	0.219797788	5.444026549	2.444674102	
O15400	STX7		332590000	357060000	505530000	526080000	336920000	491960000	398393333.3	451653333.3	0.5391956	1.133686976	0.181022351	
Q9UNK0	STX8		269790000	232920000	296930000	376790000	282660000	281040000	266546666.7	313496666.7	0.269798837	1.176141764	0.234061963	
P61764	STXBP1		0	0	0	114500000	75023000	82685000	0	90736000	0.001684532	N/A	N/A	
Q15833	STXBP2		81064000	0	149150000	89928000	97689000	0	76738000	62539000	0.803115147	0.814967813	-0.295185014	
O00186	STXBP3		217660000	147190000	203830000	366770000	267900000	622720000	189560000	419130000	0.100468107	2.211067736	1.144743223	
P53999	SUB1		126810000	0	0	199330000	135640000	308420000	42270000	214463333.3	0.059029238	5.073653497	2.343024995	

Q9Y2Z0	SUGT1		0	0	0	11773000	56283000	17737000	0	28597666.67	0.109676364	N/A	N/A	
Q8NBJ7	SUMF2		0	89081000	114650000	180380000	124960000	92056000	67910333.33	132465333.3	0.209930145	1.950591712	0.963911832	
O15260	SURF4		0	49942000	26443000	13524000	0	0	25461666.67	4508000	0.237884828	0.177050468	-2.497767437	
O60506	SYNCRIP		426040000	408790000	434210000	885860000	678760000	530280000	423013333.3	698300000	0.05622674	1.650775389	0.723143835	
Q8N3V7	SYNPO		273490000	221860000	317110000	287700000	165170000	166370000	270820000	206413333.3	0.25974437	0.762179061	-0.39179812	
P37802	TAGLN2		539070000	798000000	458830000	795900000	966310000	1144500000	598633333.3	968903333.3	0.061348177	1.61852553	0.694680123	
O15533	TAPBP		0	72812000	0	124360000	126900000	0	24270666.67	83753333.33	0.286496168	3.450804812	1.786932873	
Q13148	TARDBP		87215000	173360000	199060000	174760000	222150000	326010000	153211666.7	240973333.3	0.192305616	1.57281321	0.653347344	
P26639	TARS1		93597000	0	128490000	95846000	121170000	118240000	74029000	111752000	0.390224265	1.509570574	0.594138206	
Q9NUY8	TBC1D23		0	0	0	40161000	49617000	0	0	29926000	0.120510402	N/A	N/A	
Q9Y4P3	TBL2		98948000	98878000	134640000	349930000	262130000	155690000	110822000	255916666.7	0.06483901	2.309258691	1.207429798	
Q969Z0	TBRG4		180110000	460760000	0	79517000	0	214940000	213623333.3	98152333.33	0.478904825	0.459464478	-1.121974766	
Q13488	TCIRG1		96364000	158960000	0	0	72603000	90549000	85108000	54384000	0.59905766	0.638999859	-0.646112482	
P17987	TCP1		9648200000	8982700000	8774700000	6486300000	5652500000	5702400000	9135200000	5947066667	0.001073791	0.651005634	-0.619258066	
Q9Y2W6	TDRKH		0	58733000	73360000	0	33066000	79820000	44031000	37628666.67	0.852219878	0.854594869	-0.22668744	
Q9NZ01	TECR		527450000	382110000	391310000	289380000	295220000	197320000	433623333.3	260640000	0.037965209	0.601074665	-0.734383881	
Q9P273	TENM3		448240000	538700000	571760000	605240000	794130000	795630000	519566666.7	731666666.7	0.044236801	1.408224803	0.493877658	
P02786	TFRC		3777200000	3560100000	3981000000	4704800000	4999900000	4745100000	3772766667	4816600000	0.002392957	1.276675826	0.352392242	
P61812	TGFB2		97914000	110160000	108810000	142240000	108730000	93988000	105628000	114986000	0.561355722	1.088593933	0.122465901	
P21980	TGM2		0	0	0	156540000	93276000	202590000	0	150802000	0.008910401	N/A	N/A	
Q8IYQ7	THNSL1		86750000	94090000	0	88150000	98687000	0	60280000	62279000	0.965545818	1.033161911	0.047066363	
P04216	THY1		857750000	782190000	709150000	1323400000	1110200000	851810000	783030000	1095136667	0.09436726	1.39858839	0.483971434	
P31483	TIA1		38572000	0	36844000	0	0	0	25138666.67	0	0.1163253	0	N/A	
Q9NQ88	TIGAR		0	0	0	76006000	113690000	0	0	63232000	0.131570991	N/A	N/A	
P01033	TIMP1		0	0	0	278320000	329710000	269990000	0	292673333.3	9.67953E-05	N/A	N/A	
Q07157	TJP1		677220000	547790000	668250000	660430000	574220000	322340000	631086666.7	518996666.7	0.364559932	0.822385726	-0.28211287	
Q9UDY2	TJP2		215900000	157550000	128910000	70425000	146480000	129350000	167453333.3	115418333.3	0.205274016	0.689256708	-0.536886691	
P29401	TKT		0	0	0	85386000	102850000	118600000	0	102278666.7	0.000438185	N/A	N/A	
Q9Y490	TLN1		5676900000	5131100000	4746400000	5865800000	5801800000	6260900000	5184800000	5976166667	0.060794016	1.152632053	0.204932044	
Q9Y4G6	TLN2		110730000	105560000	0	0	0	0	72096666.67	0	0.116343731	0	N/A	
Q99805	TM9SF2		185620000	0	0	232220000	222160000	324320000	61873333.33	259566666.7	0.047415917	4.195129835	2.068715461	
Q9HD45	TM9SF3		654430000	295900000	240130000	374110000	404210000	997680000	396820000	592000000	0.463397046	1.491860289	0.577112435	
Q92544	TM9SF4		528800000	430720000	561860000	318340000	332750000	199460000	507126666.7	283516666.7	0.017955276	0.559064796	-0.838912592	
P49755	TMED10		952150000	1172000000	1252500000	1276800000	861010000	1049100000	1125550000	1062303333	0.695003474	0.943808212	-0.08343437	
Q7Z7H5	TMED4		0	0	110950000	0	0	59226000	36983333.33	19742000	0.701958644	0.533808022	-0.90560711	
Q9Y3B3	TMED7		103780000	89649000	121130000	97448000	98822000	0	104853000	65423333.33	0.310148076	0.623952899	-0.680490968	
Q9BVK6	TMED9		621450000	760120000	828610000	699880000	721220000	466270000	736726666.7	629123333.3	0.350491934	0.853944023	-0.227786593	
Q9NUM4	TMEM106B		0	0	0	0	75492000	78015000	0	51169000	0.116223957	N/A	N/A	
Q9BVC6	TMEM109		1069600000	1178700000	1162800000	0	782150000	0	1137033333	260716666.7	0.029026631	0.229295535	-2.124719832	
Q9H061	TMEM126A		242430000	291050000	222270000	149700000	190860000	0	251916666.7	113520000	0.08753448	0.450625207	-1.150000079	
Q9HC07	TMEM165		188770000	173370000	194050000	406300000	295460000	197940000	185396666.7	299900000	0.131391742	1.617612686	0.693866217	
Q6NUQ4	TMEM214		147130000	442350000	162350000	370530000	456970000	455840000	250610000	427780000	0.151605546	1.70695503	0.771425051	
Q6PI78	TMEM65		240770000	189710000	265790000	215590000	238880000	113420000	232090000	189296666.7	0.39127321	0.815617505	-0.294035356	
P82094	TMF1		101320000	90043000	0	76451000	0	105520000	63787666.67	60657000	0.94778526	0.950920502	-0.07260336	
Q9NYL9	TMOD3		394960000	325870000	311250000	190120000	208220000	212530000	344026666.7	203623333.3	0.00627018	0.591882412	-0.756617507	
P62328	TMSB4X		0	0	0	67230000	0	43782000	0	37004000	0.13355086	N/A	N/A	
Q71RG4	TMUB2		118500000	0	0	0	71818000	0	39500000	23939333.33	0.753128673	0.606059072	-0.722469677	
Q9H3N1	TMX1		607000000	644090000	540020000	658840000	654030000	690560000	597036666.7	667810000	0.09526099	1.118541016	0.16161816	
Q9Y320	TMX2		98494000	134480000	195080000	0	196120000	0	142684666.7	65373333.33	0.338547838	0.458166493	-1.126056141	
Q96JJ7	TMX3		59858000	0	135550000	175360000	0	121660000	65136000	99006666.67	0.629980722	1.519999181	0.604070547	
Q9C0C2	TNKS1BP1		0	0	0	202700000	138160000	146530000	0	162463333.3	0.00131277	N/A	N/A	
Q92973	TNPO1		0	25572000	0	81976000	47794000	144320000	8524000	91363333.33	0.048479647	10.71836384	3.422012789	
O60784	TOM1		92597000	43999000	82609000	109020000	0	84494000	73068333.33	64504666.67	0.824579945	0.882799206	-0.179842762	
Q5JTV8	TOR1AIP1		80665000	62814000	105940000	83444000	113810000	143940000	83139666.67	113731333.3	0.227545276	1.367955128	0.452020907	
Q8NFQ8	TOR1AIP2		0	57990000	70576000	64044000	11215000	32737000	42855333.33	35998666.67	0.809305919	0.840004356	-0.251531286	
Q13641	TPBG		0	0	0	135640000	34582000	0	0	56740666.67	0.235668694	N/A	N/A	
P55327	TPD52		120270000	0	0	289500000	321820000	219050000	40090000	276790000	0.009253666	6.904215515	2.787477499	

O43399	TPD52L2		156260000	76821000	136040000	238100000	302040000	341400000	123040333.3	293846666.7	0.011258026	2.388214163	1.255932216	
P60174	TPI1		86945000	52065000	109420000	357750000	410620000	406750000	82810000	391706666.7	0.000204377	4.730185565	2.241896782	
P09493	TPM1		2574100000	3171200000	3027500000	2141200000	1982900000	1187600000	2924266667	1770566667	0.028877613	0.605473737	-0.723863712	
P06753	TPM3		228940000	275430000	426010000	524170000	337420000	0	310126666.7	287196666.7	0.895884363	0.926062469	-0.110818579	
P67936	TPM4		460160000	383810000	414080000	812400000	398180000	357420000	419350000	522666666.7	0.520987119	1.246373356	0.317736298	
O14773	TPP1		957080000	837650000	676700000	1138500000	1782400000	1223900000	823810000	1381600000	0.062445422	1.677085736	0.745956444	
P29144	TPP2		70746000	102000000	54210000	56955000	0	137140000	75652000	64698333.33	0.807893979	0.85520982	-0.225649675	
P12270	TPR		0	101340000	176930000	63989000	36062000	182100000	92756666.67	94050333.33	0.985742375	1.013946886	0.019982081	
Q14258	TRIM25		151990000	70242000	83910000	170610000	154250000	249900000	102047333.3	191586666.7	0.082661706	1.877429428	0.908758678	
Q13263	TRIM28		167890000	92212000	136030000	246220000	163130000	188310000	132044000	199220000	0.111177898	1.508739511	0.593343741	
O75962	TRIO		0	0	37082000	48965000	43887000	0	12360666.67	30950666.67	0.402240753	2.503964187	1.324213929	
Q15642	TRIP10		57928000	33280000	0	0	23941000	0	30402666.67	7980333.333	0.294102686	0.26248794	-1.929676957	
Q15643	TRIP11		1006900000	564510000	984430000	561200000	0	0	851946666.7	187066666.7	0.047953916	0.21957556	-2.18721061	
Q9UI30	TRMT112		103010000	128330000	0	0	165910000	230820000	77113333.33	132243333.3	0.52440791	1.71492176	0.778142758	
Q8TD43	TRPM4		114630000	45052000	0	0	22968000	0	53227333.33	7656000	0.253656057	0.143835874	-2.797504555	
Q99816	TSG101		0	0	60876000	61110000	0	0	20292000	20370000	0.997965392	1.003843879	0.005534915	
O75954	TSPAN9		0	0	56794000	0	104520000	0	18931333.33	34840000	0.708771471	1.840335247	0.8799686	
Q16762	TST		296740000	314980000	0	0	0	0	203906666.7	0	0.116470038	0	N/A	
Q9C0H2	TTYH3		451800000	258070000	207880000	554880000	698840000	755020000	305916666.7	669580000	0.018844287	2.188766004	1.130117727	
Q71U36	TUBA1A		136210000	0	233450000	107730000	188800000	0	123220000	98843333.33	0.793289509	0.802169561	-0.318020872	
P68363	TUBA1B		3245500000	3888100000	3711500000	4626100000	4969500000	7106300000	3615033333	5567300000	0.07099186	1.540041125	0.622968877	
P07437	TUBB		2050900000	1687600000	1744200000	1702000000	2220800000	2544700000	1827566667	2155833333	0.291104346	1.179619531	0.238321614	
P68371	TUBB4B		4203700000	3517400000	3991400000	4697800000	5385900000	5599600000	3904166667	5227766667	0.017539696	1.339022412	0.421180108	
Q3ZCM7	TUBB8		203120000	0	0	119160000	155330000	0	67706666.67	91496666.67	0.787089548	1.351368649	0.434421291	
Q6IBS0	TWF2		0	0	0	95513000	82825000	0	0	59446000	0.118125967	N/A	N/A	
P40222	TXLNA		79789000	68455000	70235000	175520000	109490000	70687000	72826333.33	118565666.7	0.211741318	1.628060363	0.703154191	
P10599	TXN		0	0	0	40710000	0	92972000	0	44560666.67	0.173051589	N/A	N/A	
O95881	TXNDC12		520410000	799740000	1019300000	856490000	1067700000	726710000	779816666.7	883633333.3	0.585461065	1.133129582	0.180312853	
Q9BRA2	TXNDC17		0	0	0	53467000	0	49816000	0	34427666.67	0.11661331	N/A	N/A	
Q8NBS9	TXNDC5		2452200000	2764200000	3072000000	3884800000	2834100000	3249100000	2762800000	3322666667	0.188980394	1.20264466	0.266210439	
P04818	TYMS		135050000	156050000	87836000	0	0	55344000	126312000	18448000	0.016873647	0.146051048	-2.775455383	
Q01081	U2AF1		122360000	231670000	47357000	91217000	42108000	147680000	133795666.7	93668333.33	0.550276351	0.700084955	-0.514398091	
P26368	U2AF2		129380000	59152000	75477000	76916000	0	116110000	88003000	64342000	0.58745752	0.731134166	-0.451791924	
P22314	UBA1		327890000	372210000	297840000	425620000	542820000	872640000	332646666.7	613693333.3	0.106825606	1.844880454	0.883527334	
Q14157	UBAP2L		0	0	0	122100000	133280000	96212000	0	117197333.3	0.00043607	N/A	N/A	
P62837	UBE2D2		112090000	0	13266000	165220000	48950000	73502000	41785333.33	95890666.67	0.340270486	2.294840295	1.198393755	
P63279	UBE2I		0	0	0	0	79269000	92418000	0	57229000	0.118444373	N/A	N/A	
P61088	UBE2N		131460000	58420000	78974000	152650000	145580000	177940000	89618000	158723333.3	0.044291703	1.77110997	0.824653794	
Q92575	UBXN4		142630000	155940000	188770000	123380000	162820000	159020000	162446666.7	148406666.7	0.492246865	0.913571634	-0.130410239	
Q9Y3C8	UFC1		0	0	0	55712000	76232000	0	0	43981333.33	0.125647043	N/A	N/A	
O94874	UFL1		313090000	223390000	433150000	382520000	423460000	466210000	323210000	424063333.3	0.197860948	1.31203655	0.39180791	
P61960	UFM1		0	69905000	0	0	0	165290000	23301666.67	55096666.67	0.623219096	2.364494671	1.241531891	
Q9NYU2	UGGT1		500970000	461820000	381960000	310860000	555600000	658020000	448250000	508160000	0.611109738	1.133653095	0.180979234	
P11172	UMPS		0	0	0	0	22072000	29732000	0	17268000	0.124739812	N/A	N/A	
Q92900	UPF1		0	0	0	0	76754000	29483000	0	35412333.33	0.188338346	N/A	N/A	
O60763	USO1		47186000	48077000	58312000	135640000	74637000	35874000	51191666.67	82050333.33	0.351022596	1.602806446	0.680600217	
Q14694	USP10		0	92070000	90828000	177570000	147600000	119110000	60966000	148093333.3	0.066738109	2.429113495	1.280429898	
P45974	USP5		325060000	297000000	302870000	306610000	639020000	925580000	308310000	623736666.7	0.152916343	2.023082828	1.016555387	
P46939	UTRN		916470000	954260000	769300000	55044000	54291000	95043000	880010000	68126000	0.000151128	0.077415029	-3.691242511	
Q8NBZ7	UXS1		0	0	18253000	16499000	91159000	189760000	6084333.333	99139333.33	0.139417247	16.29419821	4.026286458	
Q15836	VAMP3		231110000	292900000	239280000	449390000	331790000	299220000	254430000	360133333.3	0.099861692	1.415451532	0.501262349	
Q9P0L0	VAPA		1631200000	1901100000	1813200000	1132900000	1316000000	1592400000	1781833333	1347100000	0.048947473	0.756019081	-0.403505447	
O95292	VAPB		1049300000	1209200000	984760000	916690000	1028000000	803030000	1081086667	915906666.7	0.150705727	0.847209289	-0.239209687	
P26640	VARS1		0	246140000	185280000	453290000	610710000	451700000	143806666.7	505233333.3	0.016446414	3.513281721	1.812819267	
P50552	VASP		135560000	222860000	220490000	142600000	128930000	51450000	192970000	107660000	0.102133893	0.557910556	-0.841894246	
Q99536	VAT1		373960000	259220000	471850000	678380000	730340000	588310000	368343333.3	665676666.7	0.015991652	1.807217904	0.853770468	
P18206	VCL		302230000	183270000	337190000	269050000	301330000	284810000	274230000	285063333.3	0.830808327	1.039504552	0.055896076	

P55072	VCP		4868700000	4425200000	4439500000	4700900000	5400000000	5423700000	4577800000	5174866667	0.098380448	1.130426551	0.176867257	
O15240	VGF		0	0	0	490560000	482370000	758350000	0	577093333.3	0.003122747	N/A	N/A	
P08670	VIM		22944000000	22719000000	24194000000	31044000000	32387000000	25610000000	23285666667	29680333333	0.039411531	1.274618149	0.350065108	
Q96RL7	VPS13A		115810000	100520000	0	0	0	0	72110000	0	0.118099738	0	N/A	
Q9P253	VPS18		0	175390000	165710000	67002000	127260000	131000000	113700000	108420666.7	0.934741904	0.953567869	-0.068592471	
Q96QK1	VPS35		243980000	340250000	298530000	237610000	181250000	283410000	294253333.3	234090000	0.21266134	0.795538991	-0.329995453	
P49754	VPS41		0	0	0	0	74001000	64823000	0	46274666.67	0.117852158	N/A	N/A	
Q9NRW7	VPS45		14520000	27025000	14233000	0	52667000	27816000	18592666.67	26827666.67	0.629443174	1.442916562	0.528987877	
Q86Y07	VRK2		0	116800000	0	29394000	0	0	38933333.33	9798000	0.508200415	0.251660959	-1.990446672	
Q9NP79	VTA1		397730000	293850000	259460000	0	0	0	317013333.3	0	0.00158638	0	N/A	
Q96AJ9	VTI1A		224100000	0	98417000	0	0	120950000	107505666.7	40316666.67	0.428614077	0.375018991	-1.414964438	
Q9UEU0	VTI1B		216030000	164360000	176840000	193760000	246600000	189800000	185743333.3	210053333.3	0.368957393	1.130879529	0.177445249	
A3KMH1	VWA8		0	0	87762000	0	56984000	0	29254000	18994666.67	0.783286804	0.64930152	-0.623039508	
P23381	WARS1		0	0	0	51587000	67158000	56238000	0	58327666.67	0.000225607	N/A	N/A	
Q9Y6W5	WASF2		512780000	374170000	291830000	200300000	280730000	182750000	392926666.7	221260000	0.073406834	0.56310762	-0.828517422	
Q641Q2	WASHC2A		73833000	0	67642000	50337000	60557000	0	47158333.33	36964666.67	0.752347551	0.783841668	-0.351365828	
Q9BZH6	WDR11		0	0	0	95593000	33567000	0	0	43053333.33	0.198960647	N/A	N/A	
Q8NI36	WDR36		0	0	0	0	32804000	52201000	0	28335000	0.136401	N/A	N/A	
O14980	XPO1		0	0	8602600	0	0	54259000	2867533.333	18086333.33	0.452662332	6.307279195	2.657017796	
Q9UBH6	XPR1		0	2774100	0	5423600	0	0	924700	1807866.667	0.686054929	1.955084532	0.967230987	
P13010	XRCC5		1107200000	976650000	853680000	827790000	873370000	799170000	979176666.7	833443333.3	0.128811752	0.851167477	-0.232485067	
P12956	XRCC6		1251700000	1159700000	1210700000	1516000000	1390300000	801880000	1207366667	1236060000	0.903271098	1.023765219	0.033884899	
P54577	YARS1		164030000	269250000	81256000	109270000	189380000	54109000	171512000	117586333.3	0.466583596	0.685586626	-0.544589129	
P67809	YBX1		28170000	47918000	104310000	279540000	156500000	79277000	60132666.67	171772333.3	0.149177278	2.856556059	1.514276843	
P16989	YBX3		526270000	495020000	461390000	1218900000	1060200000	527840000	494226666.7	935646666.7	0.103214572	1.893152939	0.920790965	
P07947	YES1		348280000	362050000	707950000	537280000	570110000	496800000	472760000	534730000	0.631579849	1.13108131	0.177702644	
Q9BWQ6	YIPF2		28799000	85666000	23627000	34567000	268440000	146330000	46030666.67	149779000	0.214560556	3.253895953	1.70216812	
Q969M3	YIPF5		216600000	0	0	0	0	235870000	72200000	78623333.33	0.954903041	1.088965836	0.122958693	
Q96EC8	YIPF6		55705000	43036000	0	76090000	79427000	0	32913666.67	51839000	0.573744611	1.57499924	0.655351133	
O15498	YKT6		0	0	0	109930000	100110000	0	0	70013333.33	0.116985223	N/A	N/A	
Q96TA2	YME1L1		406590000	365500000	367060000	227960000	196930000	379130000	379716666.7	268006666.7	0.125727592	0.705806961	-0.502654435	
P31946	YWHAB		137340000	74651000	124830000	234800000	335560000	218780000	112273666.7	263046666.7	0.021699908	2.342906173	1.228299179	
P62258	YWHAE		88504000	57106000	0	143580000	164710000	98117000	48536666.67	135469000	0.05558807	2.791065174	1.480815813	
P27348	YWHAQ		112660000	0	104720000	88800000	172350000	192830000	72460000	151326666.7	0.177641829	2.088416598	1.06240953	
P63104	YWHAZ		444210000	698290000	722750000	1505900000	1333000000	1006500000	621750000	1281800000	0.018278557	2.061600322	1.043764667	
Q8N4Q0	ZADH2		257070000	0	281600000	192260000	275970000	318220000	179556666.7	262150000	0.444064851	1.459984777	0.545953327	
Q8WU90	ZC3H15		0	0	147900000	183870000	0	201380000	49300000	128416666.7	0.384579308	2.604800541	1.381172904	
Q7Z2W4	ZC3HAV1		150770000	138140000	178550000	171620000	200130000	253220000	155820000	208323333.3	0.120898125	1.336948616	0.418944018	
O95159	ZFPL1		163180000	244730000	223040000	391160000	259780000	205350000	210316666.7	285430000	0.280880155	1.357143989	0.440573795	
Q68DK2	ZFYVE26		0	0	0	35195000	33449000	0	0	22881333.33	0.116373791	N/A	N/A	
O75844	ZMPSTE24		154560000	31371000	177180000	127480000	249600000	187290000	121037000	188123333.3	0.307457988	1.554263022	0.636230666	
O15231	ZNF185		132430000	161030000	158680000	293840000	203150000	146130000	150713333.3	214373333.3	0.221270181	1.422391295	0.5083184	
O43264	ZW10		352650000	378830000	279030000	205850000	241230000	296500000	336836666.7	247860000	0.089352659	0.735846256	-0.442523727	
Q15942	ZYX		67846000	0	0	0	94707000	0	22615333.33	31569000	0.828965447	1.395911329	0.481207301	
P01892	HLA-A		305760000	332900000	441930000	573710000	467090000	356700000	360196666.7	465833333.3	0.232834922	1.29327497	0.371029047	
P10319	HLA-B1		0	0	6795200	0	8962800	0	2265066.667	2987600	0.856568949	1.318989875	0.39943349	
P30481	HLA-B2		117730000	125370000	0	221900000	237040000	124460000	81033333.33	194466666.7	0.102518953	2.399835459	1.262935493	
